# Novel [(3-indolylmethylene)hydrazono]indolin-2-ones as apoptotic anti-proliferative agents: design, synthesis and *in vitro* biological evaluation

**DOI:** 10.1080/14756366.2017.1421181

**Published:** 2018-03-21

**Authors:** Wagdy M. Eldehna, Mahmoud F. Abo-Ashour, Hany S. Ibrahim, Ghada H. Al-Ansary, Hazem A. Ghabbour, Mahmoud M. Elaasser, Hanaa Y. A. Ahmed, Nesreen A. Safwat

**Affiliations:** a Department of Pharmaceutical Chemistry, Faculty of Pharmacy, Kafrelsheikh University, Kafrelsheikh, Egypt;; b Department of Pharmaceutical Chemistry, Faculty of Pharmacy, Egyptian Russian University, Badr City, Egypt;; c Department of Pharmaceutical Chemistry, Faculty of Pharmacy, Ain Shams University, Cairo, Egypt;; d Department of Pharmaceutical Chemistry, College of Pharmacy, King Saud University, Riyadh, Saudi Arabia;; e Department of Medicinal Chemistry, Faculty of Pharmacy, Mansoura University, Mansoura, Egypt;; f The Regional Center for Mycology and Biotechnology, Al-Azhar University, Cairo, Egypt

**Keywords:** Indole, apoptosis, anticancer, oxidative stress

## Abstract

On account of their significance as apoptosis inducing agents, merging indole and 3-hydrazinoindolin-2-one scaffolds is a logic tactic for designing pro-apoptotic agents. Consequently, 27 hybrids (**6a–r**, **9a–f** and **11a–c**) were synthesised and evaluated for their cytotoxicity against MCF-7, HepG-2 and HCT-116 cancer cell lines. SAR studies unravelled that *N*-propylindole derivatives were the most active compounds such as **6n** (MCF-7; IC_50_=1.04 µM), which displayed a significant decrease of cell population in the G2/M phase and significant increase in the early and late apoptosis by 19-folds in Annexin-V-FTIC assay. Also, **6n** increased the expression of caspase-3, caspase-9, cytochrome C and Bax and decreased the expression of Bcl-2. Moreover, compounds **6i**, **6j**, **6n** and **6q** generated ROS by significant increase in the level of SOD and depletion of the levels of CAT and GSH-Px in MCF-7.

## Introduction

Apoptosis, programmed cell death, is considered as an essential mechanism by the body to get rid of unwanted cells. Therefore, triggering apoptosis in cancer cells will lead to automatic death and increase the relief from cancer proliferation. Thoroughly understanding the mechanism of apoptosis reveals that it is affected by the expression of caspases, Bcl-2 family proteins, including either anti-apoptotic or pro-apoptotic members. Induction of apoptosis is considered as one of the most successful strategies to target cancer^1^.

**Figure 1.**
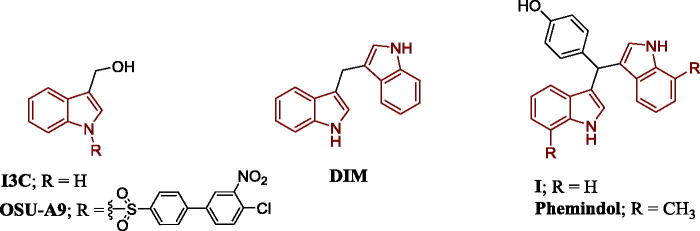
Reported indole derivatives with apoptotic activity.

Indole is a well-known interesting scaffold to generate anticancer agents through induction of apoptosis. Indole-3-carbinol (I3C) (Figure 1), a naturally occurring compound found in family Cruciferae, displayed a remarkable activity against different types of cancer cells including breast, colon, leukaemia and prostate. This activity was justified by its ability to induce apoptosis through arresting G1/S phase of cell cycle^2^^,^^3^. Interestingly, under acidic conditions, indole-3-carbinol is dimerised into 3,3′-diindolylmethane (DIM) which exhibited superior activity as apoptosis inducing agent over indole-3-carbinol^2^^,^^3^. Moreover, Phemindole (Figure 1) overcame the poor bioavailability of DIM and illustrated apoptotic activity against triple negative breast cancer cells (MDA-MB-231) through reactive oxygen species (ROS) mediated mitochondrial-dependent apoptosis^4^. Modifications for both I3C and DIM took place to get more active compounds. For example, OSU-A9, *N*-substituted derivative of I3C, was 100 times more potent than the parent compound (the range of IC_50_ of OSU-A9 against MDA-MB-231, MCF-7 and SKBR3 is 1.2–1.8 µM and IC_50_ of IC3** **=** **200 µM) with more acid stability against both breast and prostate cancer cells (Figure 1)^5^^,^^6^. In addition, 1,1-Bis(3'-indolyl)-1-(*p*-hydroxyphenyl)methane (**I**), a derivative of Phemindole, was found to promote apoptosis in both pancreatic and colon cancer cells *in vivo* as well as *in vitro*^7^^,^^8^.

**Figure 2.**
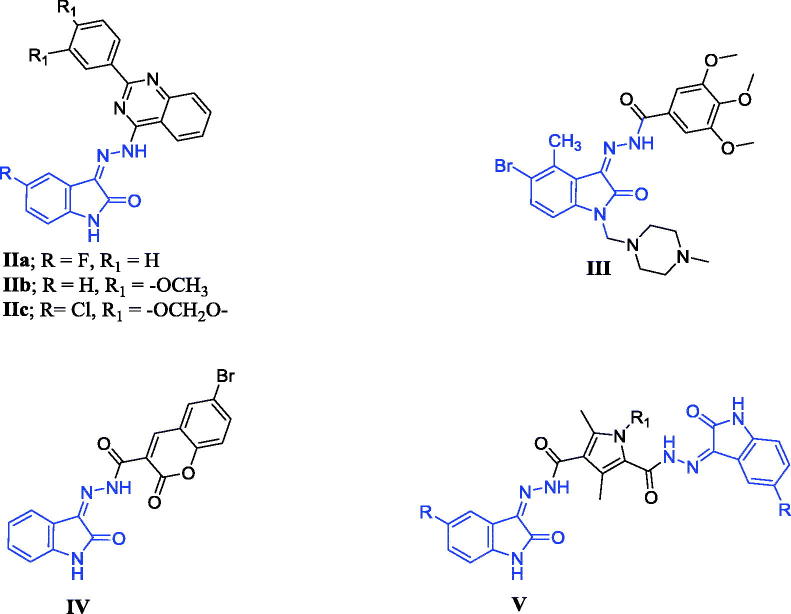
Reported 3-hydrazinoindolin-2-one derivatives (**II–V**) with apoptotic activity.

On the other hand, several 3-hydrazinoindolin-2-one derivatives proved their anticancer activity through induction of apoptosis in various cancer cells^9^. For instance, compounds **IIa–c** (Figure 2) showed cytotoxic activity against HepG2 cancer cells with IC_50_ range (1.0–2.4 µM). The apoptosis in HepG2 cells by compounds **IIa–c** was investigated through increasing the expression of Bax, a pro-apoptotic protein, and decreasing the expression of Bcl-2, an anti-apototic protein, accompanied by high levels of caspase-3^10^. Moreover, compound **III**, a water**-**soluble oxindole derivative (Figure 2), possessed EC_50_** **= 0.14 µM against human colorectal cells; HCT-116. It had an inhibitory activity against tubulin polymerisation (IC_50_** **= 0.19 µM) through induction of apoptosis^11^. Furthermore, compound **IV** had the ability to activate caspase 3/7 and promote apoptosis in Panc1 cells^12^. The use of 3-hydrazinoindolin-2-one as apoptosis inducer was augmented by designing bis-isatin hydrazones connected with linker as in compound **V** (Figure 2). It showed cytotoxic activity against two cancer cell lines, namely MCF-7 and HCT-116 with an IC_50_ of 1.84 µM and 3.31 µM, respectively. The pro-apoptotic activity of this compound was assured by cell cycle disruption and Annexin-V FTIC analysis^13^.

**Figure 3.**
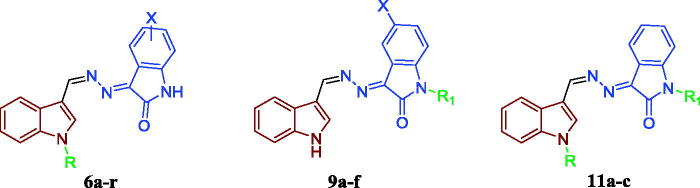
The designed compounds (**6a–r**, **9a–f** and **11a–c**) as apoptosis-inducing agents.

Inspired by these findings and as a part of our ongoing efforts towards developing novel and potent anticancer agents based on the indoline-2-one moiety^14^, we herein report the design and synthesis of new three different sets of indole-indolin-2-one hybrids **6a–r**, **9a–f** and **11a–c** (Figure 3), with the prime goal of developing potent anticancer agents with potential pro-apoptotic activity. First, methylenehydrazono (HC=N–N=) linker was selected to conjugate the indole and indolin-2-one moieties. Then, three strategies were applied to develop the three hybrids series. For the first series **6a–r**, indole-3-carboxaldehyde or a variety of *N*-alkylated indole derivatives (*N*-methyl, *N*-propyl or *N*-benzyl) was conjugated with 5-/7-substituted indolin-2-one moieties. In the second series **9a–f**, indole-3-carboxaldehyde was hybridised with a variety of *N*-alkylated indolin-2-one derivatives (*N*-methyl, *N*-propyl or *N*-benzyl). Regarding the third series, it was designed so as to maintain the pharmacophoric *N*-propyl-indole moiety while exploring the chemical variation at the *N*-position of the indolin-2-one moiety, in an attempt to optimise the obtained cytotoxicity results from the first and the second series and to carry out further elaboration of the indole-indolin-2-one hybrid scaffolds to explore a valuable SAR.

All compounds will be subjected to cytotoxic activity. According to the results, the most active hybrid will be further investigated regarding its effects on cell cycle progression and potential apoptotic effect in the MCF-7 cells, to acquire perception of the mechanism of the anti-proliferative activity of the synthesised hybrids. As inhibition of apoptotic markers (as Bcl-2, Bax, Caspase 3 and 9 and cytochrome C) leads to the accumulation of ROS and results in oxidative stress^15^, some oxidative stress parameters will be taken into consideration in our biological study.

## Materials and methods

### Chemistry

Melting points were measured with a Stuart melting point apparatus and were uncorrected. Infrared (IR) Spectra were recorded as KBr disks using Schimadzu FT-IR 8400S spectrophotometer (Shimadzu, Kyoto, Japan). Mass spectral data are given by GCMS-QP1000 EX spectrometer at 70 eV. NMR spectra were recorded on a Varian Mercury NMR spectrometer. ^1^H spectrum was run at 400 MHz and ^13^C spectrum was run at 100 MHz in deuterated dimethylsulphoxide (DMSO-*d_6_*). Chemical shifts are expressed in values (*ppm*) using the solvent peak as internal standard. All coupling constant (*J*) values are given in Hertz. The abbreviations used are as follows: s, singlet; d, doublet; m, multiplet. Elemental analyses were carried out at the Regional Center for Microbiology and Biotechnology, Al-Azhar University, Cairo, Egypt. Analytical thin layer chromatography (TLC) on silica gel plates containing UV indicator was employed routinely to follow the course of reactions and to check the purity of products.

#### Synthesis of 1H-indole-3-carbaldehyde

Compound **2** was prepared according to the literature procedure^16^.

##### Synthesis of 3-hydrazonoindolin-2-ones 4a–f

To a stirred solution of isatins **3a–f** (5 mmol) in methyl alcohol (10 ml), 99% hydrazine hydrate (1.25 ml, 25 mmol) was added. The reaction mixture was refluxed for 1 h, and then the formed precipitate was filtered off, washed with cold methanol, dried and recrystallised from glacial acetic acid to furnish hydrazones **4a–f**^17^.

##### Synthesis of N-substituted-1H-indole-3-carbaldehyde derivatives 5a–c

Compounds **5a–c** were prepared according to the literature procedure^18^.

##### General procedure for the synthesis of target compounds 6a–r

A mixture of 3-hydrazonoindolin-2-ones **4a–f** (1 mmol) and 1*H*-indole-3-carbaldehyde **2** or the appropriate *N*-substituted-1*H*-indole-3-carbaldehyde **5a–c** (1 mmol), was refluxed in absolute ethyl alcohol in the presence of a catalytic amount of glacial acetic acid for 3 h, and then cooled to room temperature. The solid formed was collected by filtration, dried and crystallised from dioxane to produce compounds **6a–r**, respectively.

##### 3-(((1H-indol-3-yl)methylene)hydrazono)indolin-2-one (6a)

Yield 80%, m.p. 262–263 °C; IR (KBr, *ν* cm^−1^): 3409 (NH), 1697 (C=O); ^1^H NMR (DMSO-*d_6_*) *δ* (ppm): 6.89 (d, 1H, Ar-H, *J* = 8.0 Hz), 7.05 (t, 1H, Ar-H, *J* = 7.6 Hz), 7.27–7.38 (m, 3H, Ar-H), 7.53–7.55 (m, 1H, Ar-H), 8.21 (s, 1H, -CH=N-), 8.28–8.30 (m, 1H, Ar-H), 8.37 (d, 1H, Ar-H, *J* = 7.6 Hz), 8.94 (s, 1H, H-2 indole), 10.74 (s, 1H, NH isatin, D_2_O exchangeable), 12.14 (s, 1H, NH indole, D_2_O exchangeable); ^13^C NMR (DMSO-*d*_6_) *δ ppm*: 111.04, 112.66, 113.13, 117.60, 121.70, 122.36, 122.47, 123.89, 124.96, 128.11, 133.16, 136.84, 138.03, 144.70, 149.58, 162.28, 165.82 (C=O); MS *m/z* [%]: 288 [M^+^, 31.53] 116 [100]; Anal. calcd. For C_17_H_12_N_4_O: C, 70.82; H, 4.20; N, 19.43; Found C, 71.04; H, 4.27; N, 19.70.

##### 3-(((1H-indol-3-yl)methylene)hydrazono)-5-chloroindolin-2-one (6b)

Yield 75%, m.p. > 300 °C; IR (KBr, *ν* cm^−1^): 3427 (NH), 1703 (C=O); ^1^H NMR (DMSO-*d_6_*) *δ* (ppm): 6.90 (d, 1H, Ar-H, *J* = 8.0 Hz), 7.26–7.33 (m, 2H, Ar-H), 7.41 (dd, 1H, Ar-H, *J* = 8.4, 2.4 Hz), 7.55 (d, 1H, Ar-H, *J* = 7.6 Hz), 8.26–8.29 (m, 2H, –CH=N– and Ar-H), 8.50 (d, 1H, Ar-H, *J* = 2.4 Hz), 9.00 (s, 1H, H-2 indole), 10.87 (s, 1H, NH isatin, D_2_O exchangeable), 12.23 (s, 1H, NH indole, D_2_O exchangeable); ^13^C NMR (DMSO-*d*_6_) *δ ppm*: 111.17, 112.76, 113.37, 117.99, 121.60, 122.35, 124.15, 124.82, 126.66, 127.31, 132.18, 138.10, 138.17, 144.32, 148.07, 164.08, 164.15 (C=O); Anal. calcd. For C_17_H_11_ClN_4_O: C, 63.26; H, 3.44, N, 17.36; Found C, 63.43; H, 3.49; N, 17.61.

##### 3-(((1H-indol-3-yl)methylene)hydrazono)-5-bromoindolin-2-one (6c)

Yield 75%, m.p. > 300 °C; IR (KBr, *ν* cm^−1^): 3381 (NH), 1695 (C=O); ^1^H NMR (DMSO-*d_6_*) *δ* (ppm): 6.87 (d, 1H, Ar-H, *J* = 8.0 Hz), 7.30–7.55 (m, 4H, Ar-H), 8.25–8.28 (m, 2H, –CH=N– and Ar-H), 8.68 (s, 1H, Ar-H), 9.00 (s, 1H, H-2 indole), 10.88 (s, 1H, NH isatin, D_2_O exchangeable), 12.24 (s, 1H, NH indole, D_2_O exchangeable); ^13^C NMR (DMSO-*d*_6_) *δ ppm*: 112.82, 112.95, 113.34, 113.71, 119.25, 121.67, 122.34, 124.13, 124.81, 130.47, 135.09, 137.99, 138.15, 143.59, 148.78, 163.88, 165.47 (C=O); MS *m/z* [%]: 369 [(M + 2)^+^, 1.21], 367 [M^+^, 1.66], 116 [100]; Anal. calcd. For C_17_H_11_BrN_4_O: C, 55.61; H, 3.02; N, 15.26; Found C, 55.89; H, 2.98; N, 15.44.

##### 3-(((1H-indol-3-yl)methylene)hydrazono)-5-methoxyindolin-2-one (6d)

Yield 83%, m.p. 291–293 °C; IR (KBr, *ν* cm^−1^): 3395 (NH), 1711 (C=O); ^1^H NMR (DMSO-*d_6_*) *δ* (ppm): 3.78 (s, 3H, OCH_3_), 6.80 (d, 1H, Ar-H, *J* = 8.4 Hz), 6.95 (dd, 1H, Ar-H, *J* = 8.4, 2.4 Hz), 7.19 (t, 1H, Ar-H, *J* = 8.0 Hz), 7.27 (t, 1H, Ar-H, *J* = 8.4 Hz), 7.53 (d, 1H, Ar-H, *J* = 8.4 Hz), 8.08 (d, 1H, Ar-H, *J* = 2.8 Hz), 8.21 (s, 1H, -CH=N-), 8.35 (d, 1H, Ar-H, *J* = 8.4 Hz), 8.95 (s, 1H, H-2 indole), 10.53 (s, 1H, NH isatin, D_2_O exchangeable), 12.16 (s, 1H, NH indole, D_2_O exchangeable); ^13^C NMR (DMSO-*d*_6_) *δ ppm*: 55.98 (OCH_3_), 111.76, 112.76, 112.77, 113.23, 117.92, 119.83, 121.86, 122.06, 124.02, 124.91, 137.23, 138.09, 138.50, 150.07, 155.07, 162.78, 166.01 (C=O); MS *m/z* [%]: 318 [M^+^, 1.32]; Anal. calcd. For C_18_H_14_N_4_O_2_: C, 67.92; H, 4.43; N, 17.60; Found C, 68.17; H, 4.52; N, 17.89.

##### 3-(((1-Methyl-1H-indol-3-yl)methylene)hydrazono)indolin-2-one (6e)

Yield 78%, m.p. 255–257 °C; IR (KBr, *ν* cm^−1^): 3386 (NH), 1701 (C=O); ^1^H NMR (DMSO-*d_6_*) *δ* (ppm): 3.90 (s, 3H, CH_3_), 6.88 (d, 1H, Ar-H, *J* = 8.0 Hz), 7.04 (t, 1H, Ar-H, *J* = 7.6 Hz), 7.33–7.38 (m, 3H, Ar-H), 7.60–7.62 (m, 1H, Ar-H), 8.21 (s, 1H, -CH=N-), 8.27–8.29 (m, 1H, Ar-H), 8.36 (d, 1H, Ar-H, *J* = 7.6 Hz), 8.92 (s, 1H, H-2 indole), 10.73 (s, 1H, NH isatin, D_2_O exchangeable), ^13^C NMR (DMSO-*d*_6_) *δ ppm*: 33.77 (CH_3_), 111.05, 111.59 (2C), 117.63, 121.77, 122.49, 122.68, 123.94, 125.49, 128.16, 133.17, 138.63, 139.85, 144.74, 149.58, 161.76, 165.83 (C=O); Anal. calcd. For C_18_H_14_N_4_O: C, 71.51; H, 4.67; N, 18.53; Found C, 71.68; H, 4.52; N, 18.81.

##### 5-Chloro-3-(((1-methyl-1H-indol-3-yl)methylene)hydrazono)indolin-2-one (6f)

Yield 80%, m.p. 285–287 °C; IR (KBr, *ν* cm^−1^): 3414 (NH), 1708 (C=O); ^1^H NMR (DMSO-*d_6_*) *δ* (ppm): 3.92 (s, 3H, CH_3_), 6.88 (d, 1H, Ar-H, *J* = 8.0 Hz), 7.31–7.54 (m, 4H, Ar-H), 8.23–8.28 (m, 2H, -CH=N- and Ar-H), 8.63 (s, 1H, Ar-H), 8.99 (s, 1H, H-2 indole), 10.73 (s, 1H, NH isatin, D_2_O exchangeable); ^13^C NMR (DMSO-*d*_6_) *δ ppm*: 33.87 (CH_3_), 111.68, 111.86, 112.51, 118.75, 121.70, 122.65, 124.19, 125.31, 126.08, 127.59, 132.40, 138.79, 140.96, 143.29, 148.78, 163.23, 165.58 (C=O); Anal. calcd. For C_18_H_13_ClN_4_O: C, 64.20; H, 3.89; N, 16.64; Found C, 64.43; H, 3.93; N, 16.87.

##### 5-Bromo-3-(((Z)-(1-methyl-1H-indol-3-yl)methylene)hydrazono)indolin-2-one (6g)

Yield 77%, m.p. 295–297 °C; IR (KBr, *ν* cm^−1^): 3390 (NH), 1695 (C=O); ^1^H NMR (DMSO-*d_6_*) *δ* (ppm): 3.91 (s, 3H, CH_3_), 6.86 (d, 1H, Ar-H, *J* = 7.6 Hz), 7.33–7.38 (m, 2H, Ar-H), 7.54 (d, 1H, Ar-H, *J* = 7.2 Hz), 7.63 (d, 1H, Ar-H, *J* = 7.2 Hz), 8.24–8.29 (m, 2H, -CH=N- and Ar-H), 8.66 (s, 1H, Ar-H), 8.97 (s, 1H, H-2 indole), 10.87 (s, 1H, NH isatin, D_2_O exchangeable); ^13^C NMR (DMSO-*d*_6_) *δ ppm*: 33.84 (N-CH_3_), 111.71, 111.79, 112.92, 113.69, 119.22, 121.75, 122.64, 124.16, 125.23, 130.43, 135.06, 138.75, 141.07, 143.56, 148.70, 163.38, 165.45 (C=O); Anal. calcd. For C_18_H_13_BrN_4_O: C, 56.71; H, 3.44, N, 14.70; Found C, 57.02; H, 3.48; N, 14.96.

##### 5-Methoxy-3-(((1-methyl-1H-indol-3-yl)methylene)hydrazono)indolin-2-one (6h)

Yield 82%, m.p. 281–283 °C; IR (KBr, *ν* cm^−1^): 3427 (NH), 1705 (C=O); ^1^H NMR (DMSO-*d_6_*) *δ* (ppm): 3.79 (s, 3H, OCH_3_), 3.92 (s, 3H, CH_3_), 6.79 (d, 1H, Ar-H, *J* = 8.0 Hz), 6.93 (dd, 1H, Ar-H, *J* = 8.0, 2.4 Hz), 7.21 (t, 1H, Ar-H, *J* = 8.0 Hz), 7.32 (t, 1H, Ar-H, *J* = 8.0 Hz), 7.55 (d, 1H, Ar-H, *J* = 8.4 Hz), 8.06 (d, 1H, Ar-H, *J* = 2.4 Hz), 8.19 (s, 1H, -CH=N-), 8.34 (d, 1H, Ar-H, *J* = 8.4 Hz), 8.96 (s, 1H, H-2 indole), 10.49 (s, 1H, NH isatin, D_2_O exchangeable); ^13^C NMR (DMSO-*d*_6_) *δ ppm*: 33.74 (N-CH_3_), 55.92 (OCH_3_), 111.64 (2 C), 111.71, 112.68, 117.87, 119.77, 121.94, 122.43, 124.01, 125.29, 138.46, 138.65, 140.35, 149.99, 155.02, 162.30, 165.97 (C=O); MS *m/z* [%]: 332 [M^+^, 67.77], 289 [100]; Anal. calcd. For C_19_H_16_N_4_O_2_: C, 68.66; H, 4.85, N, 16.86; Found C, 68.91; H, 4.92; N, 17.09.

##### 3-(((1-Propyl-1H-indol-3-yl)methylene)hydrazono)indolin-2-one (6i)

Yield 75%, m.p. 118–120 °C; IR (KBr, *ν* cm^−1^): 3415 (NH), 1698 (C=O); ^1^H NMR (DMSO-*d_6_*) *δ* (ppm): 0.85 (t, 3H, –CH_2_–CH_2_–C*H*_3_, *J* = 7.2 Hz), 1.81 (sextet, 2H, –CH_2_–C*H*_2_–CH_3_, *J* = 7.2 Hz), 4.24 (t, 2H, –C*H*_2_–CH_2_–CH_3_, *J* = 7.2 Hz), 6.88 (d, 1H, Ar-H, *J* = 8.0 Hz), 7.04 (t, 1H, Ar-H, *J* = 8.0 Hz), 7.33–7.38 (m, 3H, Ar-H), 7.66–7.68 (m, 1H, Ar-H), 8.26 (s, 1H, -CH=N-), 8.28–8.30 (m, 1H, Ar-H), 8.36 (d, 1H, Ar-H, *J* = 7.6 Hz), 8.92 (s, 1H, H-2 indole), 10.73 (s, 1H, NH isatin, D_2_O exchangeable); ^13^C NMR (DMSO-*d*_6_) *δ ppm*: 11.53 (–CH_2_–CH_2_–*C*H_3_), 23.28 (–CH_2_–*C*H_2_–CH_3_), 48.26 (–*C*H_2_–CH_2_–CH_3_), 111.05, 111.70, 111.74, 117.64, 121.93, 122.49, 122.62, 123.92, 125.56, 128.15, 133.18, 138.01, 139.08, 144.70, 149.61, 161.80, 165.83 (C=O); Anal. calcd. For C_20_H_18_N_4_O: C, 72.71; H, 5.49, N, 16.96; Found C, 72.88; H, 5.56; N, 17.24.

##### 5-Chloro-3-(((1-propyl-1H-indol-3-yl)methylene)hydrazono)indolin-2-one (6j)

Yield 80%, m.p. 141–142 °C; IR (KBr, *ν* cm^−1^): 3406 (NH), 1712 (C=O); ^1^H NMR (DMSO-*d_6_*) *δ* (ppm): 0.85, 1.02 (2t, 3H, –CH_2_–CH_2_–C*H*_3_, *J* = 7.6 Hz), 1.80, 3.40 (2sextet, 2H, –CH_2_–C*H*_2_–CH_3_, *J* = 7.2 Hz), 4.25, 4.31 (2t, 2H, –C*H*_2_–CH_2_–CH_3_, *J* = 7.2 Hz), 6.83, 6.90 (2d, 1H, Ar-H, *J* = 8.4 Hz), 7.14, 7.41 (2dd, 1H, Ar-H, *J* = 8.4, 2.0 Hz), 7.29–7.38 (m, 3H, Ar-H), 7.60, 7.69 (2d, 1H, Ar-H, *J* = 8.0 Hz), 8.29, 8.30 (2 s, 1H, -CH=N-), 8.48 (d, 1H, Ar-H, *J* = 2.4 Hz), 8.97, 8.99 (2 s, 1H, H-2 indole), 10.78, 10.87 (2 s, 1H, NH isatin, D_2_O exchangeable); ^13^C NMR (DMSO-*d*_6_) *δ ppm*: 11.45, 11.51 (–CH_2_–CH_2_–*C*H_3_), 23.15, 23.24 (–CH_2_–*C*H_2_–CH_3_), 48.28, 48.33 (–*C*H_2_–CH_2_–CH_3_), 111.82, 111.84, 111.95, 112.49, 117.40, 117.52, 118.74, 121.51, 121.64, 121.89, 122.58, 122.85, 123.91, 124.15, 124.53, 125.15, 125.40, 125.51, 126.10, 126.80, 127.60, 132.38, 137.52, 137.62, 138.15, 140.11, 143.27, 148.84, 163.08, 163.22, 165.60, 184.89; MS *m/z* [%]: 366 [(M + 2)^+^, 24.47], 364 [M^+^, 69.06], 143 [100]; Anal. calcd. For C_20_H_17_ClN_4_O: C, 65.84; H, 4.70; N, 15.36; Found C, 66.09; H, 4.74; N, 15.53.

##### 5-Bromo-3-(((1-propyl-1H-indol-3-yl)methylene)hydrazono)indolin-2-one (6k)

Yield 83%, m.p. 126–128 °C; IR (KBr, *ν* cm^−1^): 3381 (NH), 1697 (C=O); ^1^H NMR (DMSO-*d_6_*) *δ* (ppm): 0.88 (t, 3H, –CH_2_–CH_2_–C*H*_3_, *J* = 7.2 Hz), 1.84 (sextet, 2H, –CH_2_–C*H*_2_–CH_3_, *J* = 7.2 Hz), 4.27 (t, 2H, –C*H*_2_–CH_2_–CH_3_, *J* = 7.2 Hz), 6.86 (d, 1H, Ar-H, *J* = 8.0 Hz), 7.31–7.36 (m, 2H, Ar-H), 7.54 (d, 1H, Ar-H, *J* = 8.0 Hz), 7.69 (d, 1H, Ar-H, *J* = 8.0 Hz), 8.29–8.31 (m, 2H, -CH=N- and Ar-H), 8.66 (s, 1H, Ar-H), 8.97 (s, 1H, H-2 indole), 10.87 (s, 1H, NH isatin, D_2_O exchangeable); ^13^C NMR (DMSO-*d*_6_) *δ ppm*: 11.05 (–CH_2_–CH_2_–*C*H_3_), 23.22 (–CH_2_–*C*H_2_–CH_3_), 48.32 (–*C*H_2_–CH_2_–CH_3_), 111.82, 111.90, 112.98, 113.69, 119.22, 121.89, 122.61, 124.17, 125.32, 130.42, 135.12, 138.15, 140.32, 143.59, 148.77, 163.41, 165.44 (C=O); Anal. calcd. For C_20_H_17_BrN_4_O: C, 58.69; H, 4.19; N, 13.69; Found C, 58.95; H, 4.26; N, 14.02.

##### 5-Methoxy-3-(((1-propyl-1H-indol-3-yl)methylene)hydrazono)indolin-2-one (6l)

Yield 80%, m.p. 122–124 °C; IR (KBr, *ν* cm^−1^): 3361 (NH), 1696 (C=O); ^1^H NMR (DMSO-*d_6_*) *δ* (ppm): 0.86 (t, 3H, –CH_2_–CH_2_–C*H*_3_, *J* = 7.2 Hz), 1.81 (sextet, 2H, –CH_2_–C*H*_2_–CH_3_, *J* = 7.2 Hz), 3.78 (s, 3H, OCH_3_), 4.24 (t, 2H, –C*H*_2_–CH_2_–CH_3_, *J* = 7.2 Hz), 6.81 (d, 1H, Ar-H, *J* = 8.0 Hz), 6.94 (d, 1H, Ar-H, *J* = 8.0 Hz), 7.22 (t, 1H, Ar-H, *J* = 8.0 Hz), 7.35 (t, 1H, Ar-H, *J* = 8.0 Hz), 7.55 (d, 1H, Ar-H, *J* = 8.4 Hz), 8.09 (s, 1H, Ar-H), 8.22 (s, 1H, -CH=N-), 8.33 (d, 1H, Ar-H, *J* = 8.4 Hz), 8.97 (s, 1H, H-2 indole), 10.81 (s, 1H, NH isatin, D_2_O exchangeable); ^13^C NMR (DMSO-*d*_6_) *δ ppm*: 11.51 (–CH_2_–CH_2_–*C*H_3_), 23.25 (–CH_2_–*C*H_2_–CH_3_), 48.27 (–*C*H_2_–CH_2_–CH_3_), 55.96 (OCH_3_), 111.76, 111.80 (2C), 112.74, 117.90, 119.83, 122.11, 122.32, 124.03, 125.43, 138.06, 138.51, 139.53, 150.07, 155.07, 162.32, 166.00; Anal. calcd. For C_21_H_20_N_4_O_2_: C, 69.98; H, 5.59; N, 15.55; Found C, 76.21; H, 5.66; N, 15.80.

##### 5-Fluoro-3-(((1-propyl-1H-indol-3-yl)methylene)hydrazono)indolin-2-one (6m)

Yield 75%, m.p. 135–137 °C; IR (KBr, *ν* cm^−1^): 3420 (NH), 1702 (C=O); ^1^H NMR (DMSO-*d_6_*) *δ* (ppm): 0.86 (t, 3H, –CH_2_–CH_2_–C*H*_3_, *J* = 7.6 Hz), 1.83 (sextet, 2H, –CH_2_–C*H*_2_–CH_3_, *J* = 7.2 Hz), 4.23 (t, 2H, –C*H*_2_–CH_2_–CH_3_, *J* = 7.2 Hz), 6.98–7.02 (m, 1H, Ar-H), 7.31–7.36 (m, 2H, Ar-H), 7.42 (dd, 1H, Ar-H, *J* = 8.0, 2.4 Hz), 7.59 (d, 1H, Ar-H, *J* = 8.0 Hz), 8.27–8.30 (m, 2H, -CH=N- and Ar-H), 8.45 (d, 1H, Ar-H, *J* = 2.4 Hz), 8.96 (s, 1H, H-2 indole), 10.83 (s, 1H, NH isatin, D_2_O exchangeable); MS *m/z* [%]: 348 [M^+^, 25.60], 143 [100]; ^13^C NMR (DMSO-*d*_6_) *δ ppm*: 11.49 (–CH_2_–CH_2_–*C*H_3_), 23.26 (–CH_2_–*C*H_2_–CH_3_), 48.25 (–*C*H_2_–CH_2_–CH_3_), 111.13, 111.41, 112.55, 113.96, 117.90, 119.08, 121.47, 122.35, 124.03, 125.52, 132.78, 136.62, 138.97, 143.07, 149.21, 161.83, 165.90; Anal. calcd. For C_20_H_17_FN_4_O: C, 68.95; H, 4.92; N, 16.08; Found C, 69.18; H, 4.97; N, 16.24.

##### 7-Fluoro-3-(((1-propyl-1H-indol-3-yl)methylene)hydrazono)indolin-2-one (6n)

Yield 79%, m.p. 129–131 °C; IR (KBr, *ν* cm^−1^): 3418 (NH), 1705 (C=O); ^1^H NMR (DMSO-*d_6_*) *δ* (ppm): 0.85 (t, 3H, –CH_2_–CH_2_–C*H*_3_, *J* = 7.6 Hz), 1.81 (sextet, 2H, –CH_2_–C*H*_2_–CH_3_, *J* = 7.2 Hz), 4.24 (t, 2H, –C*H*_2_–CH_2_–CH_3_, *J* = 7.2 Hz), 7.09–7.12 (m, 1H, Ar-H), 7.29–7.376 (m, 3H, Ar-H), 7.67–7.69 (m, 1H, Ar-H), 8.23–8.30 (m, 3H, –CH=N– and 2 Ar-H), 8.95 (s, 1H, H-2 indole), 11.27 (s, 1H, NH isatin, D_2_O exchangeable); ^13^C NMR (DMSO-*d*_6_) *δ ppm*: 11.52 (–CH_2_–CH_2_–*C*H_3_), 23.29 (–CH_2_–*C*H_2_–CH_3_), 48.23 (–*C*H_2_–CH_2_–CH_3_), 111.13, 111.41, 112.98, 116.87, 119.16, 121.23, 123.85, 125.48, 127.04, 131.23, 135.47, 138.56, 142.61, 147.08, 152.32, 162.72, 166.04; Anal. calcd. For C_20_H_17_FN_4_O: C, 68.95; H, 4.92; N, 16.08; Found C, 69.12; H, 4.87; N, 16.31.

##### 3-(((1-Benzyl-1H-indol-3-yl)methylene)hydrazono)indolin-2-one (6o)

Yield 75%, m.p. 261–263 °C; IR (KBr, *ν* cm^−1^): 3422 (NH), 1697 (C=O); ^1^H NMR (DMSO-*d_6_*) *δ* (ppm): 5.55 (s, 2H, benzylic CH_2_), 6.88 (d, 1H, Ar-H, *J* = 8.0 Hz), 7.04 (t, 1H, Ar-H, *J* = 7.2 Hz), 7.27–7.38 (m, 8H, Ar-H), 7.61 (dd, 1H, Ar-H, *J* = 6.8, 2.0 Hz), 8.30 (dd, 1H, Ar-H, *J* = 6.8, 2.0 Hz), 8.34 (d, 1H, Ar-H, *J* = 8.0 Hz), 8.39 (s, 1H, -CH=N-), 8.94 (s, 1H, H-2 indole), 10.74 (s, 1H, NH isatin, D_2_O exchangeable); ^13^C NMR (DMSO-*d*_6_) *δ ppm*: 50.21 (benzylic CH_2_), 110.41, 111.07, 112.04, 112.15, 117.53, 117.90, 121.80, 122.50, 122.75, 124.06, 127.48, 127.77, 128.16, 129.19, 133.26, 137.40, 137.88, 139.15, 144.77, 149.75, 161.51, 163.22, 165.77 (C=O); Anal. calcd. For C_24_H_18_N_4_O: C, 76.17; H, 4.79; N, 14.81; Found C, 76.29; H, 4.87; N, 14.04.

##### 3-(((1-Benzyl-1H-indol-3-yl)methylene)hydrazono)-5-chloroindolin-2-one (6p)

Yield 80%, m.p. 218–220 °C; IR (KBr, *ν* cm^−1^): 3395 (NH), 1703 (C=O); ^1^H NMR (DMSO-*d_6_*) *δ* (ppm): 5.57 (s, 2H, benzylic CH_2_), 6.90 (d, 1H, Ar-H, *J* = 8.4 Hz), 7.14 (dd, 1H, Ar-H, *J* = 8.4, 2.0 Hz), 7.27–7.36 (m, 6H, Ar-H), 7.42 (dd, 1H, Ar-H, *J* = 8.4, 2.0 Hz), 7.64–7.66 (m, 1H, Ar-H), 8.29–8.31 (m, 1H, Ar-H), 8.42 (s, 1H, -CH=N-), 8.46 (d, 1H, Ar-H, *J* = 2.0 Hz), 8.99 (s, 1H, H-2 indole), 10.88 (s, 1H, NH isatin, D_2_O exchangeable); ^13^C NMR (DMSO-*d*_6_) *δ ppm*: 49.61, 50.31, 111.19, 111.29, 111.85, 112.25, 112.30, 112.53, 117.41, 118.03, 118.70, 119.67, 120.19, 121.92, 122.47, 122.71, 123.71, 124.30, 124.54, 125.52, 125.57, 126.10, 126.13, 126.34, 126.38, 126.81, 127.59, 127.65, 127.71, 127.82, 128.27, 128.50, 129.00, 129.22, 132.48, 136.74, 137.27, 137.62, 138.05, 138.36, 138.42, 140.14, 143.35, 149.06, 163.02, 163.08, 163.19, 165.57; Anal. calcd. For C_24_H_17_ClN_4_O: C, 69.82; H, 4.15; N, 13.57; Found C, 70.01; H, 4.21; N, 13.74.

##### 3-(((1-Benzyl-1H-indol-3-yl)methylene)hydrazono)-5-bromoindolin-2-one (6q)

Yield 78%, m.p. 210–213 °C; IR (KBr, *ν* cm^−1^): 3428 (NH), 1705 (C=O); ^1^H NMR (DMSO-*d_6_*) *δ* (ppm): 5.56 (s, 2H, benzylic CH_2_), 6.81 (d, 1H, Ar-H, *J* = 8.0 Hz), 7.31–7.42 (m, 8H, Ar-H), 7.55 (d, 1H, Ar-H, *J* = 8.0 Hz), 7.65 (d, 1H, Ar-H, *J* = 7.6 Hz), 8.29–8.31 (m, 1H, Ar-H), 8.42 (s, 1H, -CH=N-), 8.64 (s, 1H, Ar-H), 9.00 (s, 1H, H-2 indole), 10.88 (s, 1H, NH isatin, D_2_O exchangeable); MS *m/z* [%]: 459 [(M + 2)^+^, 5.93], 457 [M^+^, 6.83], 116 [100]; ^13^C NMR (DMSO-*d*_6_) *δ ppm*: 50.32, 112.36, 113.03, 113.82, 120.17, 121.99, 122.77, 124.33, 125.00, 125.36, 127.84, 128.30, 129.24, 129.61, 130.52, 135.23, 137.28, 138.01, 140.32, 143.71, 149.03, 162.94, 163.22, 165.46; Anal. calcd. For C_24_H_17_BrN_4_O: C, 63.03; H, 3.75; N, 12.25; Found C, 63.26; H, 3.78; N, 12.51.

##### 3-(((1-Benzyl-1H-indol-3-yl)methylene)hydrazono)-5-methoxyindolin-2-one (6r)

Yield 76%, m.p. 222–224 °C; IR (KBr, *ν* cm^−1^): 3408 (NH), 1705 (C=O); ^1^H NMR (DMSO-*d_6_*) *δ* (ppm): 3.75 (s, 3H, OCH_3_), 5.55 (s, 2H, benzylic CH_2_), 6.80 (d, 1H, Ar-H, *J* = 8.4 Hz), 6.95 (dd, 1H, Ar-H, *J* = 8.4, 2.4 Hz), 7.24–7.34 (m, 7H, Ar-H), 7.63 (d, 1H, Ar-H, *J* = 8.0 Hz), 8.03 (d, 1H, Ar-H, *J* = 2.8 Hz), 8.37–8.39 (m, 2H, –CH=N– and Ar-H), 8.95 (s, 1H, H-2 indole), 10.54 (s, 1H, NH isatin, D_2_O exchangeable); ^13^C NMR (DMSO-*d*_6_) *δ ppm*: 50.21 (benzylic CH_2_), 55.94 (–OCH_3_), 111.97, 112.12, 112.22, 112.73, 117.81, 119.92, 122.10, 122.46, 124.17, 125.55, 127.81 (2C), 128.24, 129.20 (2C), 137.36, 137.94, 138.54, 139.61, 150.21, 155.04, 162.12, 165.91 (C=O); Anal. calcd. For C_25_H_20_N_4_O_2_: C, 73.51; H, 4.94; N, 13.72; Found C, 73.84; H, 4.98; N, 13.96.

##### Synthesis of N-substituted-indoline-2,3-dione 7a–f

Compounds **7a–f** were prepared according to the literature procedure^19^^,^^20^.

##### Synthesis of N-substituted-3-hydrazonoindolin-2-ones 8a–f

Following the same procedures descried above for the preparation of hydrazones **4a–f**.

Compounds **8a**, **e**, **f**^20^ and **8c**^21^ are previously reported.

##### 5-Chloro-3-hydrazono-1-methylindolin-2-on (8b)

Yield 65%, m.p. 159–161 °C; IR (KBr, *ν* cm^−1^): 3304 (NH_2_), 1698 (C=O); ^1^H NMR (DMSO-*d_6_*) *δ* (ppm): 3.12, 3.16 (2 s, 3H, N-CH_3_), 6.97, 7.01 (2d, 1H, H-7 isatin, *J* = 8.4 Hz), 7.21 (dd, 1H, H-6 isatin, *J* = 8.4, 2.4 Hz), 7.31, 8.06 (2d, 1H, H-4 isatin, *J* = 2.4 Hz), 9.87, 10.57 (2d, 2H, NH_2_, D_2_O exchangeable, *J* = 14.4 Hz); ^13^C NMR (DMSO-*d*_6_) *δ ppm*: 25.69 (N-CH_3_), 26.13 (N-CH_3_), 109.87, 110.58, 117.13, 117.64, 122.31, 123.56, 124.61, 126.25, 126.44, 126.63, 126.67, 128.16, 138.94, 140.67, 161.09, 164.68 (C=O); Anal. calcd. For C_9_H_8_ClN_3_O: C, 51.57; H, 3.85; N, 20.05; Found C, 51.69; H, 3.87; N, 19.93.

##### 5-Chloro-3-hydrazono-1-propylindolin-2-one (8d)

Yield 72%, m.p. 133–135 °C; IR (KBr, *ν* cm^−1^): 3310 (NH_2_), 1703 (C=O); ^1^H NMR (DMSO-*d_6_*) *δ* (ppm): 0.80 (t, 3H, -CH_2_-CH_2_-C*H*_3_, *J* = 7.2 Hz), 1.51 (sextet, 2H, -CH_2_-C*H*_2_-CH_3_, *J* = 7.2 Hz), 3.60 (t, 2H, -C*H*_2_-CH_2_-CH_3_, *J* = 7.2 Hz), 7.05 (d, 1H, H-7 isatin, *J* = 8.4 Hz), 7.18 (dd, 1H, H-6 isatin, *J* = 8.4, 2.0 Hz), 7.32 (d, 1H, H-4 isatin, *J* = 2.0 Hz), 9.89, 10.61 (2d, 2H, NH_2_, D_2_O exchangeable, *J* = 14.4 Hz); ^13^C NMR (DMSO-*d*_6_) *δ ppm*: 11.55, 11.57 (–CH_2_–CH_2_–*C*H_3_), 21.00, 21.09 (–CH_2_–*C*H_2_–CH_3_), 40.65, 40.91 (–*C*H_2_–CH_2_–CH_3_), 110.03, 110.74, 117.24, 117.74, 122.45, 123.66, 124.53, 126.12, 126.34, 126.52, 126.64, 128.11, 138.25, 140.01, 161.07 (C=O), 164.66 (C=O); Anal. calcd. For C_11_H_12_ClN_3_O: C, 55.59; H, 5.09; N, 17.68; Found C, 55.43; H, 5.14; N, 17.80.

##### General procedure for the synthesis of target compounds 9a–f

Following the same procedures descried above for the preparation of compounds **6a–r**.

##### 3-(((1H-indol-3-yl)methylene)hydrazono)-1-methylindolin-2-one (9a)

Yield 70%, m.p. 283–285 °C; IR (KBr, *ν* cm^−1^): 3410 (NH), 1698 (C=O); ^1^H NMR (DMSO-*d_6_*) *δ* (ppm): 3.19 (s, 3H, N-CH_3_), 7.08 (d, 1H, Ar-H, *J* = 8.0 Hz), 7.13 (t, 1H, Ar-H, *J* = 7.6 Hz), 7.28–7.33 (m, 2H, Ar-H), 7.44 (t, 1H, Ar-H, *J* = 8.0 Hz), 7.52–7.55 (m, 1H, Ar-H), 8.22 (s, 1H, -CH=N-), 8.28–8.30 (m, 1H, Ar-H), 8.42 (d, 1H, Ar-H, *J* = 7.2 Hz), 8.97 (s, 1H, H-2 indole), 12.17 (s, 1H, NH indole, D_2_O exchangeable); ^13^C NMR (DMSO-*d*_6_) *δ ppm*: 26.41 (N-CH_3_), 109.68, 112.66, 113.15, 116.88, 121.70, 122.42, 123.00, 123.93, 124.95, 127.79, 133.07, 137.06, 138.04, 145.75, 148.89, 162.72, 164.41 (C=O); MS *m/z* [%]: 302 [M^+^, 20.95], 273 [100]; Anal. calcd. For C_18_H_14_N_4_O: C, 71.51; H, 4.67; N, 18.53; Found C, 71.67; H, 4.72; N, 18.79.

##### 3-(((1H-indol-3-yl)methylene)hydrazono)-5-chloro-1-methylindolin-2-one (9b)

Yield 65%, m.p. > 300 °C; IR (KBr, *ν* cm^−1^): 3387 (NH), 1697 (C=O); ^1^H NMR (DMSO-*d_6_*) *δ* (ppm): 3.19 (s, 3H, N-CH_3_), 7.11 (d, 1H, Ar-H, *J* = 8.4 Hz), 7.26–7.33 (m, 2H, Ar-H), 7.51 (dd, 1H, Ar-H, *J* = 8.4, 2.4 Hz), 7.55 (d, 1H, Ar-H, *J* = 7.6 Hz), 8.27–8.28 (m, 2H, -CH=N- and Ar-H), 8.53 (d, 1H, Ar-H, *J* = 2.0 Hz), 9.01 (s, 1H, H-2 indole), 12.25 (s, 1H, NH indole, D_2_O exchangeable); MS *m/z* [%]:438 [(M + 2)^+^, 8.1], 336 [M^+^, 21.32], 116 [100]; ^13^C NMR (DMSO-*d*_6_) *δ ppm*: 26.63, 111.28, 112.85, 113.47, 118.68, 121.85, 122.43, 124.30, 124.91, 125.13, 127.44, 132.24, 138.26 (2C), 144.57, 147.93, 164.13, 164.15; Anal. calcd. For C_18_H_13_ClN_4_O: C, 64.20; H, 3.89; N, 16.64; Found C, 64.43; H, 3.94; N, 16.87.

##### 3-(((1H-indol-3-yl)methylene)hydrazono)-1-propylindolin-2-one (9c)

Yield 64%, m.p. 274–275 °C; IR (KBr, *ν* cm^−1^): 3438 (NH), 1704 (C=O); ^1^H NMR (DMSO-*d_6_*) *δ* (ppm): 0.87 (t, 3H, –CH_2_–CH_2_–C*H*_3_, *J* = 7.2 Hz), 1.59 (sextet, 2H, –CH_2_–C*H*_2_–CH_3_, *J* = 7.2 Hz), 3.68 (t, 2H, –C*H*_2_–CH_2_–CH_3_, *J* = 7.2 Hz), 7.12–7.15 (m, 2H, Ar-H), 7.28–7.33 (m, 2H, Ar-H), 7.42 (t, 1H, Ar-H, *J* = 7.6 Hz), 7.52–7.55 (m, 1H, Ar-H), 8.23 (s, 1H, -CH=N-), 8.28–8.30 (m, 1H, Ar-H), 8.43 (d, 1H, Ar-H, *J* = 7.2 Hz), 8.96 (s, 1H, H-2 indole), 12.17 (s, 1H, NH indole, D_2_O exchangeable); ^13^C NMR (DMSO-*d*_6_) *δ ppm*: 11.61 (–CH_2_–CH_2_–*C*H_3_), 20.86 (–CH_2_–*C*H_2_–CH_3_), 41.25 (–*C*H_2_–CH_2_–CH_3_), 109.87, 112.68, 113.16, 116.93, 121.70, 122.43, 122.88, 123.94, 124.95, 127.97, 133.09, 137.08, 138.05, 145.13, 148.77, 162.66, 164.41 (C=O); MS *m/z* [%]: 330 [M^+^, 12.07], 273 [100]; Anal. calcd. For C_20_H_18_N_4_O: C, 72.71; H, 5.49; N, 16.96; Found C, 72.98; H, 5.53; N, 17.23.

##### 3-(((1H-indol-3-yl)methylene)hydrazono)-5-chloro-1-propylindolin-2-one (9d)

Yield 75%, m.p. 295–297 °C; IR (KBr, *ν* cm^−1^): 3415 (NH), 1702 (C=O); ^1^H NMR (DMSO-*d_6_*) *δ* (ppm): 0.86 (t, 3H, –CH_2_–CH_2_–C*H*_3_, *J* = 7.2 Hz), 1.57 (sextet, 2H, –CH_2_–C*H*_2_–CH_3_, *J* = 7.2 Hz), 3.68 (t, 2H, –C*H*_2_–CH_2_–CH_3_, *J* = 7.2 Hz), 7.18 (d, 1H, Ar-H, *J* = 8.4 Hz), 7.26–7.34 (m, 2H, Ar-H), 7.50 (dd, 1H, Ar-H, *J* = 8.4, 2.0 Hz), 7.56 (d, 1H, Ar-H, *J* = 8.4 Hz), 8.27–8.29 (m, 2H, -CH=N- and Ar-H), 8.55 (d, 1H, Ar-H, *J* = 2.4 Hz), 9.02 (s, 1H, H-2 indole), 12.26 (s, 1H, NH indole, D_2_O exchangeable); ^13^C NMR (DMSO-*d*_6_) *δ ppm*: 11.61, 20.83, 41.47, 111.46, 112.82, 113.43, 118.12, 121.63, 122.40, 124.20, 124.87, 126.61, 126.93, 127.52, 132.25, 138.23, 143.76, 148.31, 164.07, 164.08; Anal. calcd. For C_20_H_17_ClN_4_O: C, 65.84; H, 4.70; N, 15.36; Found C, 66.01; H, 4.78; N, 15.49.

##### 3-(((1H-indol-3-yl)methylene)hydrazono)-1-benzylindolin-2-one (9e)

Yield 70%, m.p. 243–245 °C; IR (KBr, *ν* cm^−1^): 3419 (NH), 1702 (C=O); ^1^H NMR (DMSO-*d_6_*) *δ* (ppm): 4.98 (s, 2H, benzylic CH_2_), 6.99 (d, 1H, Ar-H, *J* = 8.0 Hz), 7.11 (t, 1H, Ar-H, *J* = 7.6 Hz), 7.24–7.39 (m, 8H, Ar-H), 7.54–7.56 (m, 1H, Ar-H), 8.23 (s, 1H, -CH=N-), 8.28–8.30 (m, 1H, Ar-H), 8.45 (d, 1H, Ar-H, *J* = 7.2 Hz), 9.00 (s, 1H, H-2 indole), 12.19 (s, 1H, NH indole, D_2_O exchangeable); ^13^C NMR (DMSO-*d*_6_) *δ ppm*: 43.12 (benzylic CH_2_), 110.27, 112.71, 113.19, 117.10, 121.72, 122.48, 123.19, 123.97, 124.97, 127.69 (2C), 127.92, 127.98, 129.17 (2C), 132.97, 136.73, 137.29, 138.08, 144.69, 148.59, 162.94, 164.58 (C=O); MS *m/z* [%]: 378 [M^+^, 5.01] 91 [100]; Anal. calcd. For C_24_H_18_N_4_O: C, 76.17; H, 4.79; N, 14.81; Found C, 76.44; H, 4.85; N, 15.06.

##### 3-(((1H-indol-3-yl)methylene)hydrazono)-1-benzyl-5-chloroindolin-2-one (9f)

Yield 73%, m.p. 264–266 °C; IR (KBr, *ν* cm^−1^): 3380 (NH), 1698 (C=O); ^1^H NMR (DMSO-*d_6_*) *δ* (ppm): 4.98 (s, 2H, benzylic CH_2_), 7.01 (d, 1H, Ar-H, *J* = 8.4 Hz), 7.24–7.34 (m, 7H, Ar-H), 7.44 (dd, 1H, Ar-H, *J* = 8.4, 2.0 Hz), 7.56 (d, 1H, Ar-H, *J* = 7.6 Hz), 8.28–8.29 (m, 2H, -CH=N- and Ar-H), 8.57 (d, 1H, Ar-H, *J* = 2.0 Hz), 9.05 (s, 1H, H-2 indole), 12.28 (s, 1H, NH indole, D_2_O exchangeable); ^13^C NMR (DMSO-*d*_6_) *δ ppm*: 43.24 (benzylic CH_2_), 111.73, 112.83, 113.42, 118.26, 121.61, 122.42, 124.21, 124.83, 126.93, 127.51 (2C), 127.66, 127.98, 129.19 (2C), 132.14, 136.43, 138.20, 138.34, 143.23, 147.79, 164.31, 164.32 (C=O); MS *m/z* [%]: 414 [(M + 2)^+^, 1.41], 412 [M^+^, 3.85], 91 [100]; Anal. calcd. For C_24_H_17_ClN_4_O: C, 69.82; H, 4.15; N, 13.57; Found C, 70.04; H, 4.18; N, 13.81

##### Synthesis of 3-(hydrazonomethyl)-1-propyl-1H-indole 10

To a stirred solution of 1-propyl-1*H*-indole-3-carbaldehyde **5b** (1.9 g, 10 mmol) in ethanol (15 ml), 99% hydrazine hydrate (2.5 ml, 50 mmol) was added. The reaction mixture was refluxed for 2 h, and then the formed precipitate upon cooling was filtered off, washed with water, dried and recrystallised from methanol to afford hydrazone **10**. Yield 68%, m.p. 127–129 °C; IR (KBr, *ν* cm^−1^): 3294 (NH_2_); ^1^H NMR (DMSO-*d_6_*) *δ* (ppm): 0.83 (t, 3H, –CH_2_–CH_2_–C*H*_3_, *J* = 7.6 Hz), 1.75 (sextet, 2H, –CH_2_–C*H*_2_–CH_3_, *J* = 7.6 Hz), 4.13 (t, 2H, –C*H*_2_–CH_2_–CH_3_, *J* = 7.2 Hz), 7.17–7.27 (m, 2H, Ar-H), 7.54 (d, 1H, Ar-H, *J* = 8.4 Hz), 7.92 (s, 1H, -CH=N-), 8.33 (d, 1H, Ar-H, *J* = 8.0 Hz), 8.85 (s, 1H, H-2 indole); ^13^C NMR (DMSO-*d*_6_) *δ ppm*: 11.56 (–CH_2_–CH_2_–*C*H_3_), 23.40 (–CH_2_–*C*H_2_–CH_3_), 47.86 (-*C*H_2_-CH_2_-CH_3_), 110.90, 111.60, 121.18, 122.75, 123.10, 125.70, 134.89, 137.55, 155.08; Anal. calcd. For C_12_H_15_N_3_: C, 71.61; H, 7.51; N, 20.88; Found C, 71.82; H, 7.43; N, 20.72.

#### 5.1.10. General procedure for the synthesis of target compounds 11a–c

Following the same procedures descried above for the preparation of compounds **6a–r**.

##### 1-Methyl-3-(((1-propyl-1H-indol-3-yl)methylene)hydrazono)indolin-2-one (11a)

Yield 70%, m.p. 124–126 °C; IR (KBr, *ν* cm^−1^): 1711 (C=O); ^1^H NMR (DMSO-*d_6_*) *δ* (ppm): 0.84 (t, 3H, –CH_2_–CH_2_–C*H*_3_, *J* = 6.8 Hz), 1.80 (sextet, 2H, –CH_2_–C*H*_2_–CH_3_, *J* = 6.8 Hz), 3.19 (s, 3H, N-CH_3_), 4.24 (t, 2H, –C*H*_2_–CH_2_–CH_3_, *J* = 6.8 Hz), 7.08 (d, 1H, Ar-H, *J* = 8.0 Hz), 7.12 (t, 1H, Ar-H, *J* = 8.0 Hz), 7.32–7.36 (m, 1H, Ar-H), 7.44 (dt, 1H, Ar-H, *J* = 8.0, 1.0 Hz), 7.54 (d, 1H, Ar-H, *J* = 8.0 Hz), 7.66–7.68 (m, 1H, Ar-H), 8.27–8.34 (m, 2H, -CH=N- and Ar-H), 8.40 (d, 1H, Ar-H, *J* = 7.2 Hz), 8.95 (s, 1H, H-2 indole); MS *m/z* [%]: 344 [M^+^, 29.71], 143 [100]; ^13^C NMR (DMSO-*d*_6_) *δ ppm*: 11.52, 23.28, 26.43, 48.28, 109.69, 111.70, 111.77, 116.90, 121.94, 122.69, 123.01, 123.95, 125.58, 127.83, 133.09, 138.03, 139.27, 145.79, 148.92, 162.24, 164.43; Anal. calcd. For C_21_H_20_N_4_O: C, 73.23; H, 5.85; N, 16.27; Found C, 72.98; H, 5.94; N, 16.23.

##### 1-Propyl-3-(((1-propyl-1H-indol-3-yl)methylene)hydrazono)indolin-2-one (11b)

Yield 58%, m.p. 96–98 °C; IR (KBr, *ν* cm^−1^): 1706 (C=O); ^1^H NMR (DMSO-*d_6_*) *δ* (ppm): 0.84 (t, 3H, –CH_2_–CH_2_–C*H*_3_, *J* = 7.2 Hz), 0.88 (t, 3H, –CH_2_–CH_2_–C*H*_3_, *J* = 7.2 Hz), 1.61 (sextet, 2H, –CH_2_–C*H*_2_–CH_3_, *J* = 7.2 Hz), 1.83 (sextet, 2H, –CH_2_–C*H*_2_–CH_3_, *J* = 7.2 Hz), 3.72 (t, 2H, –C*H*_2_–CH_2_–CH_3_, *J* = 7.2 Hz), 4.20 (t, 2H, –C*H*_2_–CH_2_–CH_3_, *J* = 7.2 Hz), 6.91 (d, 1H, Ar-H, *J* = 8.0 Hz), 7.15–7.19 (m, 2H, Ar-H), 7.37–7.52 (m, 3H, Ar-H), 8.25 (s, 1H, -CH=N-), 8.29–8.31 (m, 1H, Ar-H), 8.39 (d, 1H, Ar-H, *J* = 7.6 Hz), 8.94 (s, 1H, H-2 indole); ^13^C NMR (DMSO-*d*_6_) *δ ppm*: 11.63, 11.65, 19.02, 20.68, 41.47, 41.66, 116.94, 117.97, 118.01, 119.38, 119.76, 123.22, 126.24, 127.31, 133.87, 137.40, 137.92, 143.26, 144.35, 146.45, 152.76, 153.09, 162.52; MS *m/z* [%]: 372 [M^+^, 24.94], 315 [81.21], 143 [100]; Anal. calcd. For C_23_H_24_N_4_O: C, 74.17; H, 6.49; N, 15.04; Found C, 74.38; H, 6.56; N, 15.29.

##### 1-Benzyl-3-(((1-propyl-1H-indol-3-yl)methylene)hydrazono)indolin-2-one (11c)

Yield 65%, m.p. 143–145 °C; IR (KBr, *ν* cm^−1^): 1710 (C=O); ^1^H NMR (DMSO-*d_6_*) *δ* (ppm): 0.86 (t, 3H, –CH_2_–CH_2_–C*H*_3_, *J* = 7.2 Hz), 1.82 (sextet, 2H, –CH_2_–C*H*_2_–CH_3_, *J* = 7.2 Hz), 4.25 (t, 2H, –C*H*_2_–CH_2_–CH_3_, *J* = 7.2 Hz), 4.98 (s, 2H, benzylic CH_2_), 6.99 (d, 1H, Ar-H, *J* = 7.6 Hz), 7.11 (t, 1H, Ar-H, *J* = 7.6 Hz), 7.24–7.39 (m, 8H, Ar-H), 7.67–7.69 (m, 1H, Ar-H), 8.29–8.31 (m, 2H, -CH=N- and Ar-H), 8.44 (d, 1H, Ar-H, *J* = 7.2 Hz), 8.98 (s, 1H, H-2 indole); ^13^C NMR (DMSO-*d*_6_) *δ ppm*: 11.54, 26.72, 43.17, 48.32, 112.65, 114.00, 114.97, 117.33, 118.01, 118.43, 119.41, 119.72, 123.16, 124.65, 127.31, 129.19, 135.95, 136.65, 137.39, 138.52, 143.26, 144.70, 147.38, 152.74, 153.07, 162.43, 164.61; MS *m/z* [%]: 420 [M^+^, 8.91], 91 [100]; Anal. calcd. For C_27_H_24_N_4_O: C, 77.12; H, 5.75; N, 13.32; Found C, 77.40; H, 5.79; N, 13.48.

### Biological evaluation

#### Assessment of in vitro cytotoxic activity

HepG-2, HCT-116 and MCF-7 cancer cell lines were obtained from VACSERA Tissue Culture Unit. The cells were propagated in DMEM supplemented with 10% heat-inactivated foetal bovine serum, 1% L-glutamine, HEPES buffer and 50 µg/ml gentamycin. All cells were maintained at 37 °C in a humidified atmosphere with 5% CO_2_ and were subcultured two times a week. Cytotoxicity was determined following a reported procedure^22^. The relation between surviving cells and drug concentration is plotted to get the survival curve of each tumour cell line after treatment with the specified compound. The 50% inhibitory concentration (IC_50_) was estimated from graphic plots of the dose response curve for each conc. using Graphpad Prism software (San Diego, CA). The data presented are the mean of at least three separate experiments.

#### In vitro anti-proliferative activity by NCI-USA

The anticancer assays were performed in accordance with the protocol of the Drug Evaluation Branch, NCI, Bethesda, MD^23–25^. A 48 h drug exposure protocol was used and sulphorhodamine B (SRB) protein assay^26^ was applied to estimate the cell viability and growth, as reported earlier^27^^,^^28^.

#### DNA-flow cytometry analysis

To determine the effect of compound **6n** on the cell cycle distribution MCF-7 cell line; cell cycle analysis was performed using the CycleTEST™ PLUS DNA Reagent Kit (Becton Dickinson Immunocytometry Systems, San Jose, CA). Control cells with known DNA content (PBMCs) were used as a reference point for determining the DI (DNA Index) for the test samples. The cells were stained with propodium iodide stain following the procedure provided by the kit and then run on the DNA cytometer. Cell-cycle distribution was calculated using CELLQUEST software (Becton Dickinson Immunocytometry Systems, San Jose, CA).

#### Annexin V-FITC apoptosis assay

Apoptotic cells were further analysed by Annexin V-FITC/DAPI assay (Cayman Chemical, Ann Arbor, MI). Briefly, MCF-7 cells were cultured to a monolayer then treated with compound **6n** at the IC_50_ concentration (1.04 µM) as described earlier. Cells were then harvested through trypsinisation, and rinsed twice in PBS followed by binding buffer. Moreover, cells were re-suspended in 100 lL of binding buffer with the addition of 1 lL of FITC-Annexin V (Becton Dickinson BD PharmingenTM, Heidelberg, Germany) followed by an incubation period of 30 min at 4 °C. Cells were then rinsed in binding buffer and re-suspended in 150 lL of binding buffer with the addition of 1 lL of DAPI (1 lg/lL in PBS) (Invitrogen, Life Technologies, Darmstadt, Germany). Cells were then analysed using the flow cytometer BD FACS Canto II (BD Biosciences, San Jose, CA) and the results were interpreted with FlowJo7.6.4 software (Tree Star, FlowJo LLC, Ashland, OR)

#### RNA extraction, real-time PCR analysis and quantification of gene expression

The gene expression of caspase3, caspase8, caspase 9, Bax, Bcl-2 and cytochrome-C was assessed by total mRNA extraction from cells using RNeasy Mini Kit^®^, Up to 1 × 107 cells, depending on the cell line, are disrupted in Buffer RLT and homogenised & disrupted, Ethanol is then added to the lysate, creating conditions that promote selective binding of RNA to the RNeasy membrane. The sample is then applied to the RNeasy Mini spin column. Total RNA binds to the membrane, contaminants are efficiently was headway, and high-quality RNA is eluted in RNase-free water. Primer sequences for each gene were as follows: caspase-3 forward primer 5′-TGCATACTCCACAGCACCTGGTTA-3′ and reverse primer 5′-CATGGCACAAAGCGACTGGATGAA-3′; Caspase-8 forward primer 5′-TTTCACTGTGTTAGCCAGGGTGGTA-3′ and reverse primer 5′-CCTGTAATCCCAGCACTTTGGGAG-3′; Caspase-9 forward primer 5′-TCAGTGACGTCTGTGTTCAGGAGA-3′ and reverse primer 5′-TTGTTGATGATGAGGCAG TAGCCG-3′; Bcl-2 forward primer 5′-ATGACCAGACACTGACCATCCACT-3′ and reverse primer 5′-ATGTAGTGGTTCTCCTGGTGGCAA-3; Bax forward primer 5′-TCTACTTTGCCAGCAAACTGGTGC-3′ and reverse primer 5′-TGTCCAGCCCATGATGGTTCTGAT-3′; Cytochrome-C forward primer 5′-AGCTGGAGACGTTGAGAAGG-3′ and reverse primer 5′-ATCTTCGTGCCAGGGATGTA-3′; GAPDH was used as reference housekeeping gene with forward primer 5′-TTCCAGGACCAAGATCCCTCCAAA-3′ and reverse primer 5′-ATGGTGGTGAAGACACCAGTGAAC-3′.

#### DPPH-free radical scavenging activities

The samples were tested for the antioxidant activity by measuring their radical scavenging ability that was assessed by the stable 2,2′-diphenyl-1-picrylhydrazyl free radical (DPPH; purchased from Sigma; St. Louis, MO) scavenging method as modified by ElSheikh et al.^29^. In details, 150 µl (DMSO solution; 1000 µg/ml final concentration) of each sample (or ascorbic acid as a reference antioxidant) was added to 850 µl pure methanol and 10 two-fold dilutions were made from this stock solution to give good dose-response curve then 2 ml of freshly prepared 0.13 mM DPPH solution in methanol was added to each tube. For control sample, 850 µl methanol and 150 µl dimethyl sulphoxide (DMSO) were added to 2 ml DPPH solution The sample solutions were vigorously shaken on a vortex for 1 min. and then the absorbance was measured at 516 nm in a UV/VIS spectrophotometer (Spectronic Spectrometer, Milton Roy Ltd, Ivyland, PA) after 30 min. The radical scavenging ability (RSA) was calculated according to the following equation:

RSA%=[(Ac – As)/Ac]*100

where Ac is the absorbance of control DPPH sample without antioxidant; As is the absorbance of tested sample after 30 min. The concentration required for 50% radical scavenging ability (IC_50_) was calculated from the graphic plots.

#### Oxidative stress parameters

##### Total protein assay in MCF-7 cells

The total proteins were assayed in treated and non-treated MCF-7 cells according to Lowry et al.^30^. Briefly, a solution containing 0.01% cupric sulphate, 0.01% NaK Tartarate, 2% sodium carbonate and 0.1 N sodium hydroxide was added to each sample and to each tube containing BSA and water (for the standard curve of the assay). Samples were vortexed and then allowed to incubate for 10 min at RT. Then, Folin–Ciocalten phenol reagent was added to each reaction tube to a final concentration of 0.1 N. Samples were vortexed and then allowed to incubate for 30 min at RT. The absorbance of each sample tube was then read at 500 nm. A standard curve was constructed and each sample protein concentration was then calculated by interpolating within the range of values provided by the standard curve. All Lowry protein assay reagents were obtained from Sigma-Aldrich (St. Louis, MO).

##### Superoxide dismutase (SOD) activity

SOD activity was done according to Kakkar et al.^31^. SOD assay is based on the spectrophotometric assessment of the inhibition of nitro blue tetrazolium-NADH and phenazine methosulphate (PMS)-mediated formazan formation. Absorbance was measured at 560 nm. About 50% inhibition of formazan formation under the assay condition in 1 min is taken as one unit of enzyme activity/minute.

##### Catalase (CAT)activity

CAT was assayed spectrophotometrically using the method of Aebi et al.^32^. Assay is based on the principle of measurement of decomposition of H_2_O_2_ by CAT measured at 240 nm.

##### Glutathione peroxidase (GSHPx) activity

The method of Paglia and Valentine^33^ was used to measure GPx activity. Cell pellets were homogenised in stock buffer containing 55 mM potassium phosphate, 1 mM sodium azide and 1 mM EDTA. GPx activity was measured by adding 0.25 mM H2O2 in the presence of 1 mM GSH, 1 EU/ml glutathione reductase (GR) and 0.2 mM NADPH and measuring the change in absorbance at 340 nm for 5 min. GSH is oxidised by H_2_O_2_ to GSSG which is recycled by GR in presence of NADPH. Data were normalised per mg protein as determined by the Lowry protein assay.

##### Malondialdehyde (MDA) assay

MDA is the last marker of the lipid peroxidation pathway. This assay is according to the repercussion of MDA with thiobarbituric acid (TBA) that forms the MDATBA adduct that can be quantified calorimetrically^34^. Briefly, cells were collected by centrifugation and sonicated in ice-cold potassium chloride (1.15%) and centrifuged for 10 min at 3000 rpm. The resulting supernatant (1 ml) was added to 2 ml of thiobarbituric acid (TBA) reagent (15% TCA, 0.7% TBA and 0.25 N HCl) and heated at 100 °C for 15 min in a boiling bath. The sample was then placed in cold and centrifuged at 1500 rpm for 10 min. Absorbance of the supernatant was measured at 535 nm.

##### Carbonyl protein content assay

Protein carbonyl (PC) is the marker of protein oxidation. The carbonyl was identified by measuring the PC residues using dinitrophenylhydrazine (DNPH). Absorbance of the samples was measured at 370 nm^35^.

## Results and discussion

### Chemistry

**Scheme 1.**
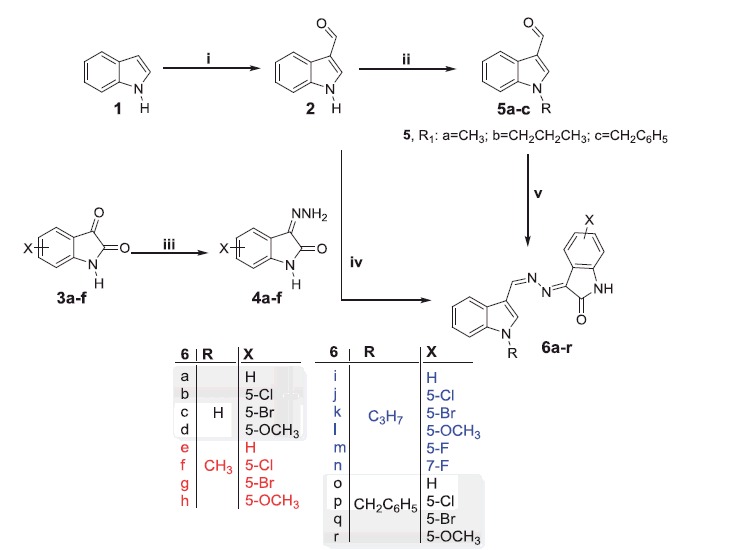
Synthesis of target compounds **6a–r**; *Reagents and conditions*: (i) dry DMF/POCl_3_/NaOH/H_2_O/2 h, (ii) alkyl (or benzyl) bromide/THF/NaH, r.t. 12 h, (iii) CH_3_OH/NH_2_NH_2_.H_2_O/reflux 1 h, (iv) EtOH/AcOH (catalytic)/reflux 3 h and (v) Hydrazones **4a–d**/EtOH/AcOH (catalytic)/reflux 3 h.

**Scheme 2.**
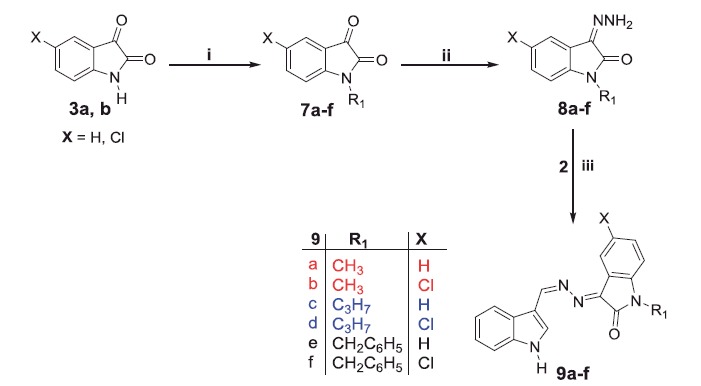
Synthesis of target compounds **9a–f**; *Reagents and conditions*: (i) methyl, propyl or benzyl bromide, dry acetonitrile, K_2_CO_3_, reflux 3 h, (ii) CH_3_OH/NH_2_NH_2_.H_2_O/reflux 1 h and (iii) EtOH/AcOH (catalytic)/reflux 3 h.

**Scheme 3.**
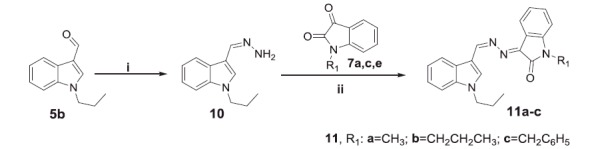
Synthesis of target compounds **11a–c**; *Reagents and conditions*: (i) EtOH/NH_2_NH_2_.H_2_O/reflux 2 h and (ii) EtOH/AcOH (catalytic)/reflux 3 h.

The proposed synthetic routes to prepare the target compounds are shown in Schemes 1–3. The synthesis of the *N*-substituted-indole-3-carbaldehyde derivatives **5a–c** was accomplished through formylation of 1*H*-indole **1** with phosphorus oxychloride and DMF to give 1*H*-indole-3-carbaldehyde **2**, then alkylation of the latter by methyl/propyl/benzyl bromide in DMF in the presence of potassium carbonate. While, refluxing isatins **3a–d** with hydrazine hydrate in methanol afforded the 3-hydrazonoindolin-2-ones **4a–d**. The first series of the target compounds **6a–r** was obtained in good yields (75–83%) through condensation of hydrazones **4a–d** with 1*H*-indole-3-carbaldehyde **2** or *N*-substituted-indole-3-carbaldehydes **5a–c** in absolute ethyl alcohol in the presence of a catalytic amount of glacial acetic acid (Scheme 1).

We next synthesised another series of hybrids (**9a–f**) to assess the impact of *N*-alkylation of the isatin moiety. Reaction of isatins **3a,b** with methyl/propyl/benzyl bromide were carried out in a refluxing dry acetonitrile in the presence of potassium carbonate to furnish *N*-alkylated isatins **7a–f**, respectively, which subsequently condensed with hydrazine hydrate in methanol to afford the hydrazones **8a–f**. The target compounds **9a–f** were obtained (64–75% yields) by reacting the hydrazone intermediates **8a–f** with *H*-indole-3-carbaldehyde **2** in a refluxing ethanol in the presence of a catalytic amount of glacial acetic acid (Scheme 2). Trials to increase the yield, by using only glacial acetic acid as a solvent, failed as it is reported that it will give another product^36^.

As a part of the SAR study, an additional series of derivatives (**11a–c**) was designed. To prepare such set of analogues (**11a–c**), the intermediate **10** was condensed with the appropriate *N*-substituted isatin **7a,c,e** in ethanol to furnish the target compounds **11a–c**, respectively, with 58–70% yields (Scheme 3).

The structures of the all synthesised compounds were confirmed under the basis of spectral and elemental analyses which were in full agreement with the proposed structures.

### Biological evaluation

#### In vitro anti-proliferative activity assay

##### Anti-proliferative activity towards MCF-7, HepG-2 and HCT-116 cell lines

Anti-proliferative activity of the synthesised compounds was proved by performing cytotoxic activity assay against three different cell lines (MCF-7, HepG-2 and HCT-116) as reported^13^. The results showed various strength of activity as shown in Table 1. Scrutinising the results reveals that compounds **6** of the first series, which is characterised by free NH of the isatin group and diverse *N*-substituents of indole moiety, illustrated their activity with respect to the substituent on NH of indole moiety. For example, compounds **6a–h**, with –NH and –N–CH_3_ on indole group, displayed no or weak cytotoxic activity against all three cell lines as they all possess IC_50_ values exceeding 24.55 µM. Grafting an *N*-propyl group on –NH of indole moiety yielded compounds with variant activity. For instance, compounds **6j** and **6k** with 5-Cl and 5-Br substituent on the isatin group exhibited moderate cytotoxic activity against the three cell lines ranging from 12.74 µM up to 25.87 µM. Exceptionally, compound **6i** with *N*-propyl indole group and unsubstituted isatin moiety and compound **6n** with *N*-propyl indole group and substituted 7-F isatin moiety, displayed significant cytotoxic activity against the three cell lines with IC_50_ values of 3.67 µM and 8.7 µM, respectively, against HCT-116 cell line and IC_50_ values of 13 µM and 3.97 µM, respectively, against HepG-2 cell line. Compounds **6i** and **6n** with the *N*-propyl indole group and unsubstituted indolin-2-one moiety or unsubstituted indolin-2-one moiety with the 7-F group, displayed strong cytotoxic activity against MCF-7 (IC_50_ = 3.58 µM and 1.04 µM) as shown in Table 1. Fortunately, introduction of benzyl group on indole moiety was fruitful in compound **6q** (HCT-116; IC_50_ = 5.99 µM, HepG-2; IC_50_ = 3.81 µM). On the other hand, all other derivatives with different substitutions on indolin-2-one moiety possessed weak or no activity.

**Table 1.** Antitumor activity of compounds **6a–u**, **9a–f,** and **11a–c** against HCT-116, HepG2, and MCF-7 cancer cell lines.
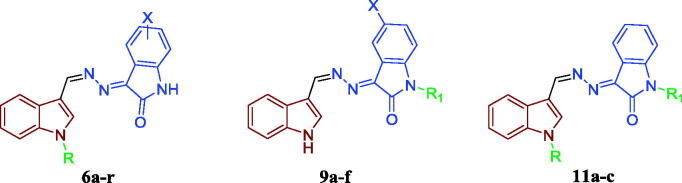

	IC_50_ (µM)^a^		
Compound	HCT-116	HepG-2	MCF-7
**6a**	>100	>100	>100
**6b**	>100	94.62 ± 6.93	>100
**6c**	>100	58.34 ± 2.38	>100
**6d**	95.46 ± 4.08	52.95 ± 1.87	>100
**6e**	37.46 ± 1.24	24.55 ± 0.47	55.34 ± 2.72
**6f**	>100	>100	>100
**6g**	>100	>100	>100
**6h**	>100	>100	>100
**6i**	3.67 ± 1.79	13.00 ± 0.67	3.58 ± 0.28
**6j**	14.00 ± 1.02	18.41 ± 1.03	25.87 ± 1.88
**6k**	25.42 ± 1.22	12.74 ± 0.83	14.38 ± 0.98
**6l**	99.14 ± 4.52	83.78 ± 3.41	>100
**6m**	95.59 ± 5.88	83.74 ± 2.29	>100
**6n**	8.57 ± 0.52	3.97 ± 0.19	1.04 ± 0.08
**6o**	80.50 ± 2.48	57.00 ± 1.44	94.00 ± 1.44
**6p**	27.80 ± 0.96	60.60 ± 2.92	27.20 ± 1.58
**6q**	5.99 ± 0.42	3.81 ± 0.13	7.67 ± 0.62
**6r**	>100	>100	>100
**9a**	>100	>100	>100
**9b**	>100	>100	>100
**9c**	>100	>100	>100
**9d**	55.30 ± 1.78	27.70 ± 1.04	98.40 ± 5.74
**9e**	>100	91.60 ± 5.44	>100
**9f**	98.20 ± 4.44	>100	>100
**11a**	70.13 ± 3.20	55.25 ± 3.18	39.42 ± 1.06
**11b**	>100	>100	>100
**11c**	>100	>100	>100
Dox.	3.70 ± 0.26	3.56 ± 0.17	2.57 ± 0.18

a IC_50_ values are the mean ± SE of three separate experiments.

Unfortunately, changing the *N*-substitution from indole moiety to isatin moiety did not display any cytotoxic activity as in compounds **9a–f** revealed by their high IC_50_ values as shown in Table 1. This gives insight that *N*-propyl substitution of the indole moiety is advantageous to anti-proliferative activity over substitution of the indolin-2-one group. Accordingly, a third series was designed **(11a–c)**, sparing the *N*-propyl indole scaffold and substituting the indolin-2-one group with different substituents. Unfortunately, none of derivatives **11a–c** proved to be a potent cytotoxic compound as they possess IC_50_ values on the three cell lines exceeding 27.7 µM.

##### NCI, USA anti-proliferative assay of 6i, 6j, 6n and 6q towards 60 cell lines

The structures of the active synthesised compounds were submitted to the National Cancer Institute (NCI) Developmental Therapeutic Program (www.dtp.nci.nih.gov). Four compounds **6i**, **6j**, **6n** and **6q** were selected to be screened for their anticancer activity *in vitro*. The compounds were screened at one dose primary anticancer assay towards a panel of 60 cancer cell lines (concentration 10^−5 ^M). The tumour cell lines were taken from nine different organs (blood, colon, lung, brain, skin, ovary, kidney, prostate and breast). The data reported as mean-graph of the percent growth of the treated cells, and presented as percentage growth inhibition (GI%) caused by the test compounds (Table 2).

**Table 2.** Percentage growth inhibition (GI %) of *in vitro* subpanel tumour cell lines at 10 μM concentration for compounds **6i**, **6j**, **6n,** and **6q**.

	Compound^a^			
Subpanel/cell line	**6i** (NSC *795310*)	**6j** (NSC *795312*)	**6n** (**NSC***795322*)	**6q** (**NSC***795327*)
Leukemia				
CCRF-CEM	24	31	66	19
HL-60(TB)	39	20	56	16
K-562	29	37	65	33
MOLT-4	26	39	52	33
RPMI-8226	25	21	38	–
SR	35	33	76	20
Non-small cell lung cancer				
A549/ATCC	–	23	12	–
EKVX	–	63	33	44
HOP-62	–	12	–	–
HOP-92	24	32	28	–
NCI-H226	12	26	–	20
NCI-H23	–	15	–	–
NCI-H322M	–	–	15	16
NCI-H460	–	26	–	48
NCI-H522	17	14	28	16
Colon cancer				
COLO 205	–	12	–	–
HCC-2998	–	–	–	–
HCT-116	12	21	20	–
HCT-15	–	25	23	–
HT29	–	14	23	–
KM12	–	24	14	–
SW-620	11	–	37	–
CNS cancer				
SF-268	–	18	13	–
SF-295	–	–	–	–
SF-539	15	–	–	–
SNB-19	–	–	–	–
SNB-75	35	19	17	15
U251	12	22	16	–
Melanoma				
LOX IMVI	17	22	16	–
MALME-3M	–	–	–	–
M14	12	20	13	–
MDA-MB-435	–	–	11	–
SK-MEL-2	–	–	–	–
SK-MEL-28	–	–	–	–
SK-MEL-5	11	20	12	–
UACC-257	18	12	17	–
UACC-62	27	25	21	21
Ovarian cancer				
IGROV1	31	50	39	19
OVCAR-3	21	–	35	–
OVCAR-4	33	12	21	16
OVCAR-5	–	–	–	72
OVCAR-8	10	21	17	–
NCI/ADR-RES	–	16	–	–
SK-OV-3	–	15	–	25
Renal cancer				
786-0	–	–	–	–
A498	–	16	–	85
ACHN	–	29	26	–
RXF 393	–	38	18	42
SN12C	11	17	17	16
TK-10	–	–	–	18
UO-31	30	47	35	24
Prostate				
PC-3	13	46	12	25
DU-145	–	14	–	–
Breast cancer				
MCF7	27	32	46	17
MDA-MB-231	11	29	18	17
HS 578T	11	29	13	19
BT-549	–	–	–	–
T-47D	29	31	50	10
MDA-MB-468	25	21	23	143
Mean growth, %	89	81	81	85
Sensitive cell lines no.	31	44	39	26

a Only GI % higher than 10% are shown.

Close examination of the obtained results, GI% values in Table 2, suggested that compounds **6j** and **6n** (*N*-propyl-indole derivatives combined with substituted indolin-2-one) are the most active analogues in this assay, showing broad spectrum activity toward numerous cell lines that belong to different tumour subpanels. Both compounds **6j** and **6n** displayed mean inhibition =19%, and possessed anti-proliferative activity against 44 and 39 cell lines, respectively, representing all subpanels (GI; 11–76%). Compound **6n** showed potent growth inhibitory effect over leukaemia CCRF-CEM, HL-60(TB), K-562, MOLT-4, SR and breast cancer MCF7, T-47D with inhibition percent of 66%, 56%, 65%, 52%, 76%, 46% and 50%, respectively. Whereas, compound **6j** showed potent growth inhibitory effect over non-small cell lung cancer EKVX, ovarian cancer IGROV1, renal cancer UO-31 and prostate cancer PC-3 with inhibition percent of 63%, 50%, 47% and 46%, respectively. All the leukaemia (except RPMI-8226) and breast cancer (except BT-549) cell lines were sensitive to all the tested compounds **6i**, **6j**, **6n** and **6q** with GI% range of 16–76% and 11–50%, respectively.

**Figure 4.**
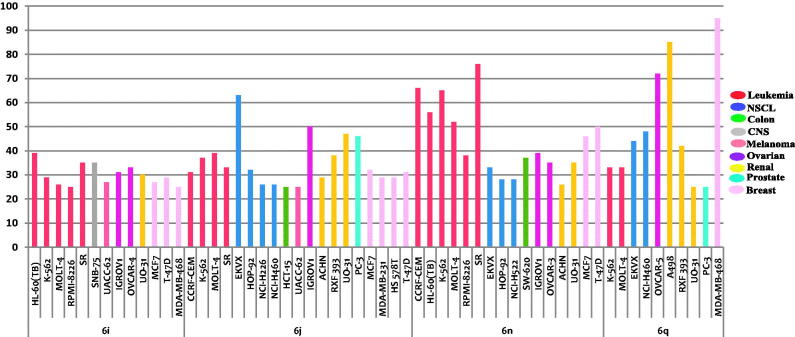
The most sensitive cell lines towards the target compounds **6i**, **6j**, **6n** and **6q** according to the GI %.

The susceptible cell lines to the tested compounds **6i**, **6j**, **6n** and **6q** with GI % ≥ 25 are presented in Figure 4. Only leukaemia K-562, MOLT-4 and renal UO-31 cell lines were susceptible to all the tested compounds, with GI % ≥ 25. While compound **6q** showed potent growth inhibition against ovarian cancer OVCAR-5 and renal cancer A498 (GI % = 72 and 85, respectively), it exerted lethal activity over breast cancer MDA-MB-468 with GI % = 143.

##### Cell-cycle analysis and apoptotic study

Compound **6n** displayed a significant cytotoxic activity against MCF-7 cancer cell lines. Consequently, more biological studies were performed to explore its mechanistic study inside this cancer cell line. Cell-cycle analysis and apoptotic cell markers would be taken into consideration during this investigative study.

##### Cell-cycle analysis

**Figure 5.**
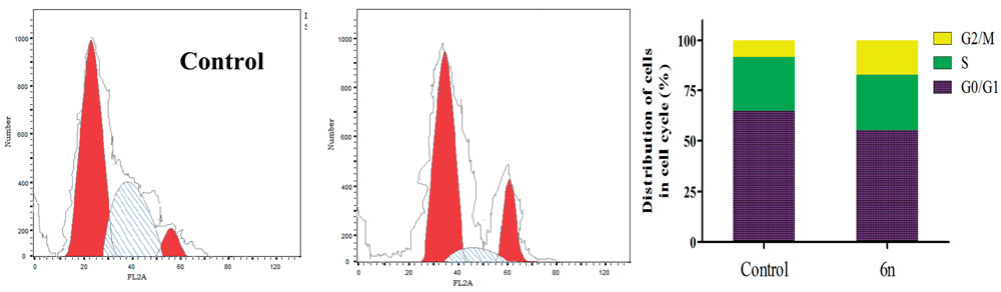
Effect of compound **6n** on the phases of cell cycle of MCF-7 cells.

Anti-proliferative agents abort cell growth by arresting its proliferation at certain well-known checkpoints^37^. Upon treatment of cancer cells with anticancer agents, distinguish cells can be detected in various phases of cell cycle. Herein, MCF-7 cells were treated with compound **6n** at its IC_50_ (1.04 µM). Analysing the obtained data, reveals that compound **6n** produced a marked decrease of the cell population at G1 and S phases as compared to the control (55.3% and 27.56%, versus 64.82% and 26.85%, respectively). Whereas it caused significant decrease of cell population in the G2/M phase when compared with untreated control cells (17.13% versus 8.33%). These results prove that compound **6n** exerts its cytotoxic effect in MCF-7 cells through arresting its proliferation in G2/M phase (Figure 5).

##### Annexin-V FTIC apoptotic study

**Figure 6.**
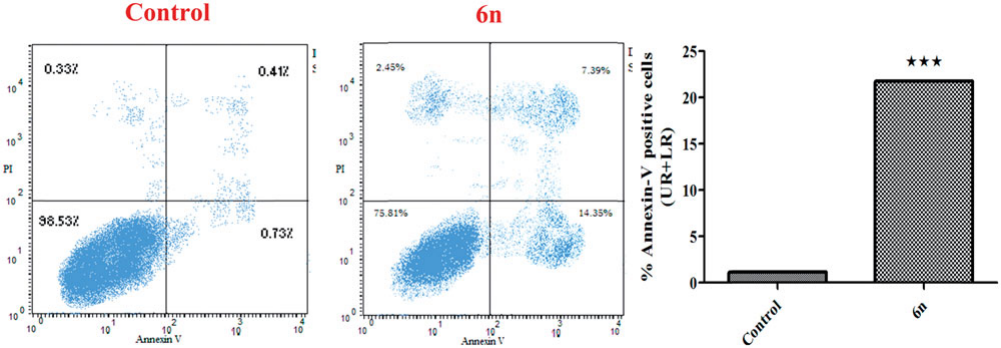
Effect of compound **6n** on the percentage of Annexin V-FITC positive staining in MCF-7 cells versus control (1% DMSO).

Annexin V-based flow cytometry analysis indicates either the cell death is achieved through programmed apoptosis or non-specific necrosis^38^. Therefore, Annexin V-FTIC/DAPI dual staining assay (Figure 6) was carried out for compound **6n** aiming to evaluate its apoptotic effect. In the current study, MCF-7 cells were treated with **6n**.

Scrutinising the results of Annexin-V FTIC assay revealed that **6n** exhibited a marked increase in the percent of Annexin V-FITC positive apoptotic cells including both the early and late apoptotic phases, from 1.14% to 21.74% which implies 19-folds increase as compared with the control (*p* < .001). Moreover, only 2.45% of the cells showed non-specific necrosis. These findings are in accordance with the previous apoptosis studies observations that propound undoubtedly that compound **6n** exhibits pro-apoptotic potential which contributes to its anti-proliferative activity.

##### Activation of proteolytic caspase cascade

Activation of the cysteine aspartic proteases, known as caspases, comprises a pivotal role in the self-automated cell death; apoptosis^39^. Consequently, the effect of compound **6n** on caspase 3, the executioner caspase, was evaluated aiming to investigate its pro-apoptotic effect. Moreover, its effect on caspase 8 and caspase 9 activations was determined in order to specify either apoptosis was induced through the intrinsic or extrinsic pathway. Results revealed that compound **6n** amazingly induced the level of active caspase 3 by 76-folds in comparison with control cells. Exploring its effects on both caspase 8 and caspase 9 revealed that it potentiated the level of the former by only 1.98-folds, whereas it produced marked increments of the latter by 928-folds. These results strongly suggest that compound **6n** induced apoptosis in MCF-7 cell line through the intrinsic rather than the extrinsic pathway (Table 3).

**Table 3.** Effect of compound **6n** on the active caspases-3, -8 and -9 levels, and the expression levels of Bcl-2, Bax and cytochrome C, respectively, in MCF-7 cancer cells treated with the compound at its IC_50_ concentration.

Compound	Casp-3 IU/ml	Casp-8 IU/ml	Casp-9 IU/ml	Bax IU/ml	Bcl-2 IU/ml	Bax/Bcl-2 ratio	Cyt-C IU/ml
Control	1733	3368	246	985	178,233	0.0055	660
**6n**	131,927***	6690**	228,320***	374,152***	165,783**	2.256	383,873***

Data are mean ± SD of three separate experiments.

* Significantly different from control (1% DMSO) at *p* < .05.

** Significantly different from control at *p* < .01.

*** Significantly different from control at *p* < .001.

##### Effects on mitochondrial apoptosis pathway (Bcl-2 family) proteins

Members of the Bcl-2 are chief regulators of the mitochondrial apoptotic pathway^40^. Among these, Bax and Bcl-2 precisely modulate this programmed process. Both proteins have opposing effects on apoptosis induction, where Bax possesses pro-apoptotic activity while Bcl-2 has anti-apoptotic effect^41^^,^^42^. Thus, the ratio between them is the determining factor for cell fate regulation^42^. Accordingly, in the current study, MCF-7 cells were treated with the IC_50_ of compound **6n** and its effect on the levels of both Bax and Bcl-2 were recorded in Table 3. Furthermore, the Bax/Bcl2 ratio was determined to give more profound insight of the pro-apoptotic activity of the molecule.

As indicated by the results in Table 3, compound **6n** amazingly boosted the Bax level 379.8-folds as compared with the control. Whereas it only downregulated the Bcl2 level by 1.07-folds. A rather decisive value is the Bax/Bcl2 ratio which gives insight to the overall pro-apoptotic effect of our compound. The calculated value for the Bax/Bcl2 ratio in comparison with that of the reference compound is increased by 407-folds. Conclusively, the ability of compound **6n** to upregulate Bax level, downregulate Bcl2 level while significantly boosting the Bax/Bcl2 ratio proves undoubtedly compound **6n** pro-apoptotic activity.

##### Cytochrome C activation

Mitochondrial cytochrome C is released into the cytosol as a result of Bax/Bcl2 increments. Accordingly, it potentiates a cascade of caspases that finally triggers the executioner caspase: caspase 3^43^^,^^44^. From the previous findings, compound **6n** proved to induce apoptosis in MCF-7 cell line through activation of the intrinsic rather than the extrinsic pathway as witnessed by the significant augmentation of caspase 9 level compared to caspase 8 level increments. Thus, we measured the level of cytochrome C to further emphasise the adoption of the intrinsic pathway. MCF-7 cells were treated with the IC_50_ of compound **6n** and the level of cytochrome C was determined as compared with the control. Results revealed that cytochrome C level increased by 581-folds (Table 3).

#### Apoptotic effect through generation of ROS

##### DPPH free radical scavenging activities

In the current study, the antioxidant potential of the synthesised compounds **6i**, **6j**, **6n** and **6q** was tested using one of the most common antioxidant assays; the DPPH (2,2-diphenyl-1-picrylhydrazyl free radical) radical scavenging assay, and were compared with standard antioxidant (ascorbic acid). DPPH is a stable free radical that has the potential to accept an electron producing a stable molecule. Compounds that have the ability to act as electron donors result in reduction of this DPPH radical revealing their antioxidant potential. The principle of the assay depends on measuring the absorbance of the odd electrons of DPPH at 516 nm. In the presence of free radical scavengers, the absorbance decreases proportional to the decrease of the DPPH radical concentration. IC_50_ values that produce 50% inhibition of the DPPH radical are given in Table 4.

**Table 4.** Evaluation of antioxidant activity for compounds **6i**, **6j**, **6n,** and **6q** using DPPH radical scavenging activity.

Compound	IC_50_ (μg/ml)^a^
**6i**	115.8 ± 6.4
**6j**	43.9 ± 0.8
**6n**	31.6 ± 0.8
**6q**	48.1 ± 1.7
Ascorbic acid	9.7 ± 0.5

a IC_50_ values are the mean ± SE of three separate experiments.

The results reveal that all the tested compounds, **6i**, **6j**, **6n** and **6q,** exhibited much higher IC_50_ values compared with that of ascorbic acid (IC_50_ = 115.8 µg/ml, 43.9 µg/ml, 31.6 µg/ml and 48.1 µg/ml, versus 9.7 µg/ml, respectively). This confirms that they have low antioxidant potential which favours the induction of oxidative stress that triggers apoptosis in neoplastic cells.

##### Oxidative stress parameters

In the current study, we evaluated the activities of some free radical enzymes including SOD, CAT, GSHPx, lipid peroxide (LP) and PC in MCF-7 cells treated with our synthesised compounds (Table 5). Moreover, the level of total protein was evaluated as an indication of the cytotoxic effect of the tested compounds.

**Table 5.** Oxidative stress markers and antioxidants in control and MCF-7 treated cells with compounds **6i**, **6j**, **6n** and **6q**.

	Compound				
ROS	**6i**	**6j**	**6n**	**6q**	Control
Total protein (μg/10^6^ cells)	59.1 ± 7.5	89.2 ± 10.4	54.6 ± 8.2	70.8 ± 6.4	131.9 ± 11.7
Superoxide dismatase (SOD) (nMol/mg protein)	137.8 ± 12.4	104.6 ± 7.8	145.2 ± 9.6	114.5 ± 10.1	64.6 ± 2.8
Catalase (CAT) (U/mg protein)	8.7 ± 2.9	22.6 ± 1.2	10.9 ± 4.1	18.4 ± 1.8	24.8 ± 3.4
Glutathione peroxidase (GSHPx) (mU/mg protein)	4.9 ± 1.3	6.7 ± 1.5	4.6 ± 0.8	5.3 ± 1.1	8.9 ± 1.8
Malondialdehyde (MDA) (nMol/mg protein)	1.75 ± 0.31	0.56 ± 0.18	1.68 ± 0.24	1.27 ± 0.15	0.42 ± 0.06
Protein carbonyl (PC) (mMol/mg protein)	16.8 ± 2.4	8.4 ± 0.4	28.1 ± 3.7	12.3 ± 1.5	6.4 ± 0.2

Data are mean ± SD of three separate experiments.

The results reveal that treatment of the MCF-7 cells with IC_50_ values of the compounds **6i**, **6j**, **6n** and **6q** elevated the level of SOD with concomitant depression of CAT and GSHPx as compared to the control cells. Additionally, the levels of ROS exemplified in LP were significantly elevated in comparison with the control cells. Moreover, the levels of PC were also elevated consequently (Table 5).

These results can thoroughly prove that the cytotoxic effect of the tested compounds **6i**, **6j**, **6n** and **6q** is partially exerted by disrupting the balance between free radical or ROS production and the antioxidant system. This is achieved through elevation of ROS (LP) levels evidenced by the significant increase in the level of SOD which triggers the production of ROS that in turn mediates apoptosis. ROS catabolising enzymes as CAT and GSH-Px levels were shown to be depleted in MCF-7 cells treated with the synthesised compounds as compared with the control which guarantees the consequent accumulation of ROS that eventually affects tumour cell killing due to protein oxidation which converts the protein to PC derivatives. In accordance, the elevated PC levels imply the protein oxidative damage as it is a reliable marker for protein damage. These results are consistent with the postulation that these compounds endeavour their cytotoxic activity through ROS generation. In accordance with these findings, the level of protein in the treated cells was lower than that of the control due to the oxidative damage of the protein by ROS over generation.

## Conclusion

Hybridisation technique between indole and 3-hydrazinoindolin-2-one was successful to produce apoptosis inducing agents. According to this technique, 27 compounds were designed and synthesised to be investigated for their cytotoxic activity against three cancer cell lines. Compound **6n** showed the best activity with IC_50_ = 1.04 µM against MCF-7 cancer cell line. This compound displayed a broad-spectrum activity against different cancer cell lines in 60-cell line panel test by NCI-USA. Deep apoptotic study on this compound revealed that this compound would disrupt G2/M phase in cell cycle and increase % of early and late apoptosis by 19-folds more than control in Annexin V-FTIC. Compound **6n** exhibited an ability to increase the expression of some enzymes that affect the apoptosis as caspase 3, caspase 9, cytochrome c, Bax and decrease the expression of Bcl-2. Compound **6n** showed the ability to induce apoptosis by generation of ROS and this shown in a further biological study on oxidative stress markers as superoxide dismutase (SOD), CAT and GSHPx.

## Supplementary Material

IENZ_1421181_Supplementary_Material.pdf
