# Supplementary material for: Novel [(3-indolylmethylene)hydrazono]indolin-2-ones as apoptotic anti-proliferative agents: design, synthesis and in vitro biological evaluation
Source: J Enzyme Inhib Med Chem. 2018 Mar 21;33(1):686–700. doi: 10.1080/14756366.2017.1421181 (PMC6010103; doi:10.1080/14756366.2017.1421181)

Supplementary Material

Novel [(3-indolylmethylene)hydrazono]indolin-2-ones as apoptotic anti-proliferative agents: Design, synthesis and *in vitro* biological evaluation

Wagdy M. Eldehna*, Mahmoud Farid, Hany S. Ibrahim, Ghada H. Al-Ansary, Hazem A. Ghabbour, Mahmoud M. Elaasser, Hanaa Y. A. Ahmed, Nesreen A. Safwat

Dr_WagdyMohamed-Z6-A

Sample Name	Dr_WagdyMohamed-Z6-A	Pulse sequence	PROTON	Temperature	25	Study owner	vnmr1
Date collected	2016-07-17	Solvent	dmso	Spectrometer	nmr400-mercury400	Operator	vnmr1

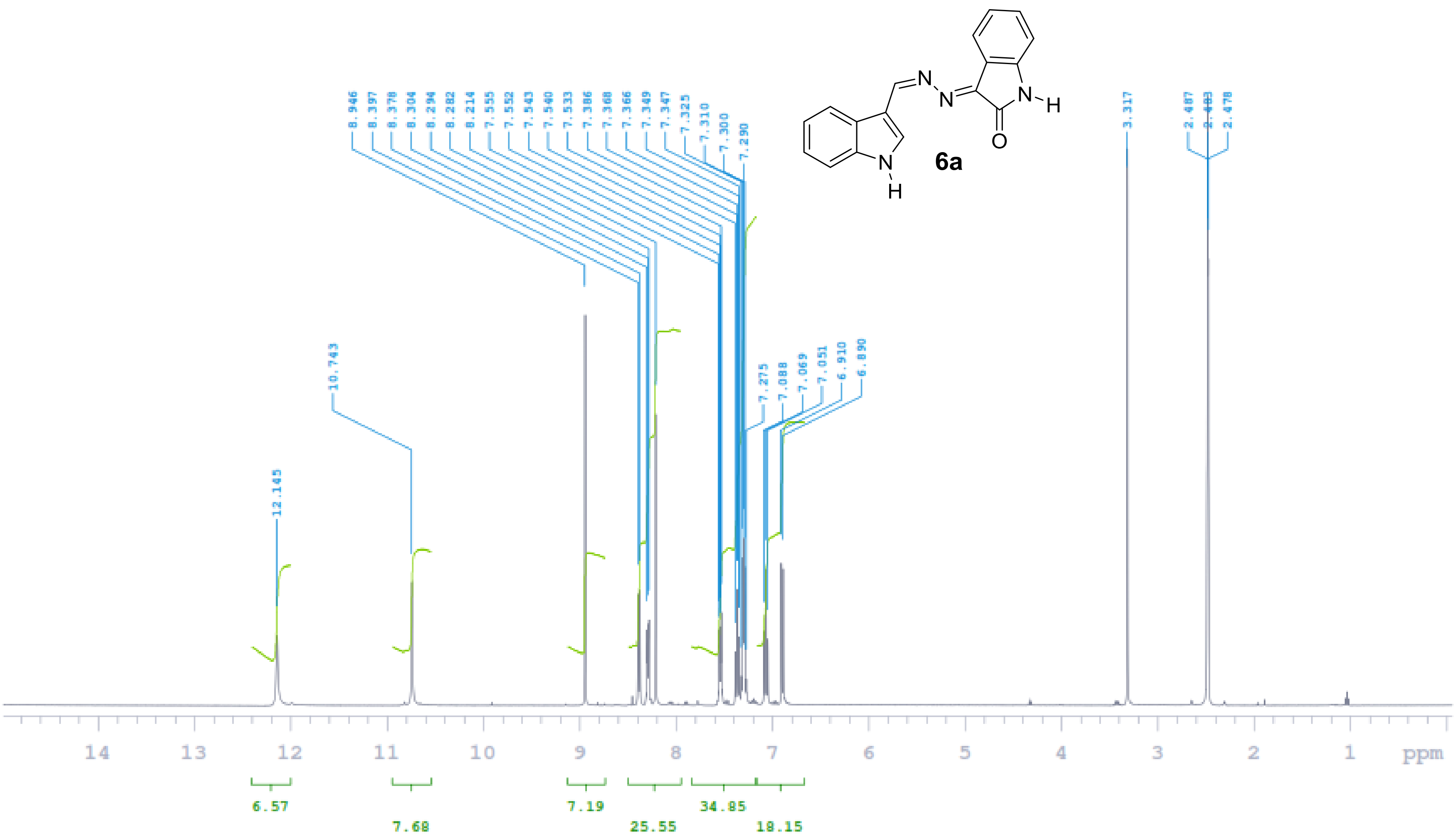

Dr_WagdyMohamed-Z6-A-D2O

Sample Name **Dr_WagdyMohamed-Z6-A-D2O** Pulse sequence **PROTON** Temperature **25** Study owner **vnmr1**
Date collected **2016-07-18** Solvent **dmso** Spectrometer **nmr400-mercury400** Operator **vnmr1**

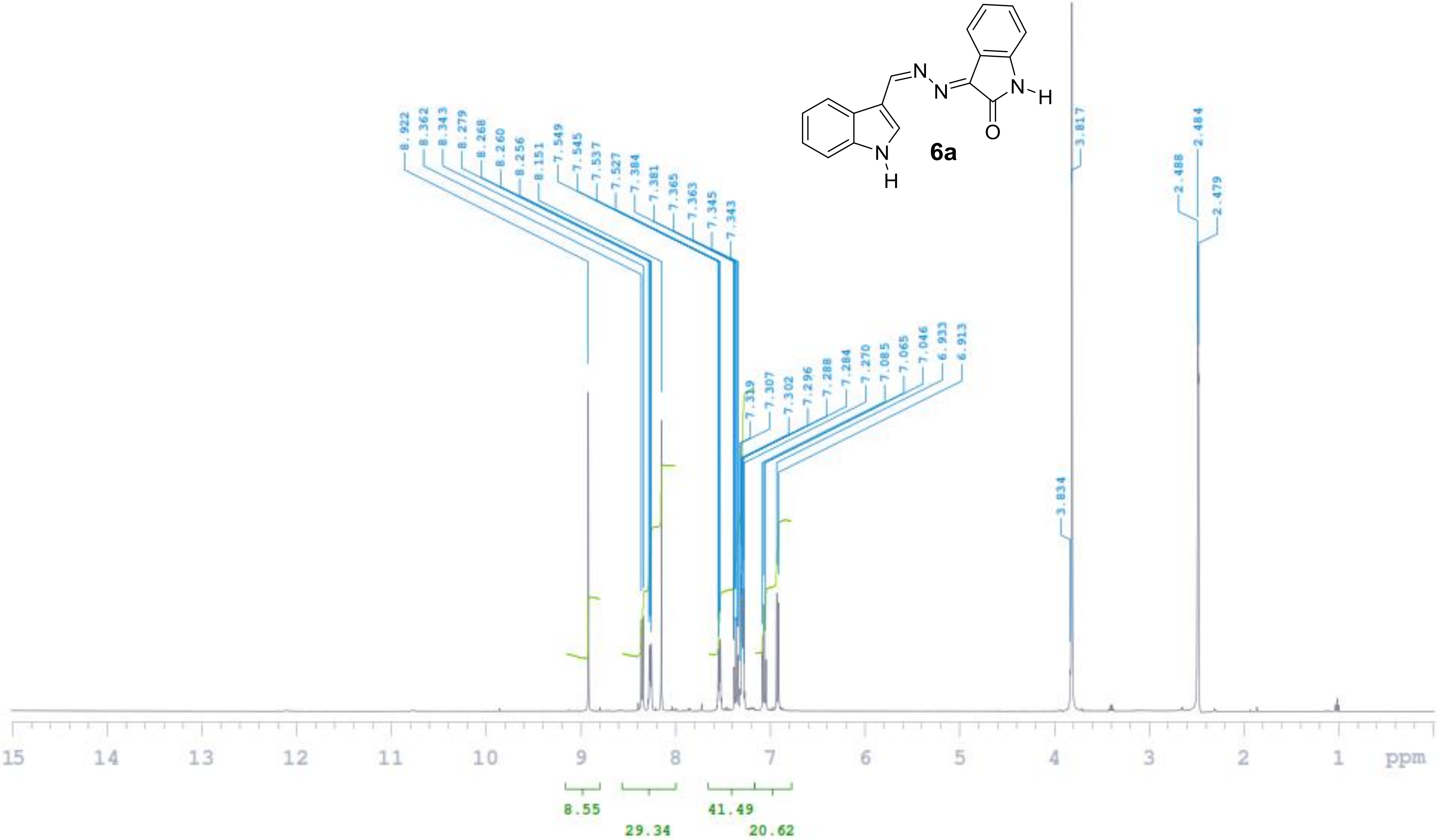

Agilent Technologies

Dr_WagdyMohamed-Z6-A

Sample Name	Dr_WagdyMohamed-Z6-A	Pulse sequence	CARBON	Temperature	25	Study owner	vnmr1
Date collected	2016-12-06	Solvent	dmso	Spectrometer	nmr400-mercury400	Operator	vnmr1

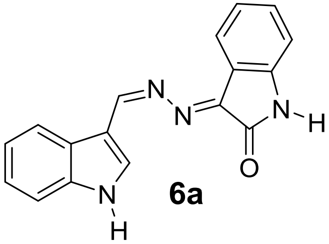

6a

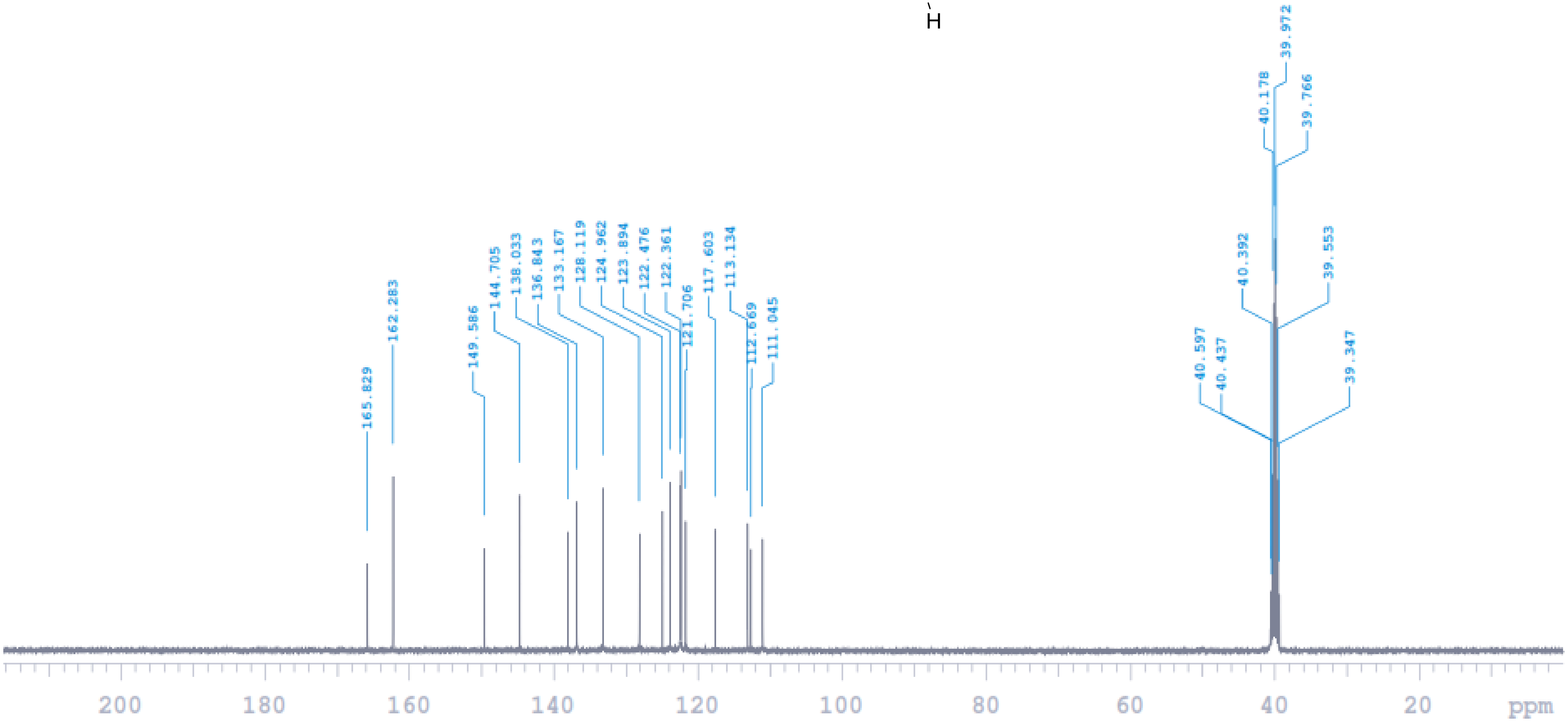

Dr_WagdyMohamed-Z6-B

Sample Name	Dr_WagdyMohamed-Z6-B	Pulse sequence	PROTON	Temperature	25	Study owner	vnmr1
Date collected	2016-07-17	Solvent	dmso	Spectrometer	nmr400-mercury400	Operator	vnmr1

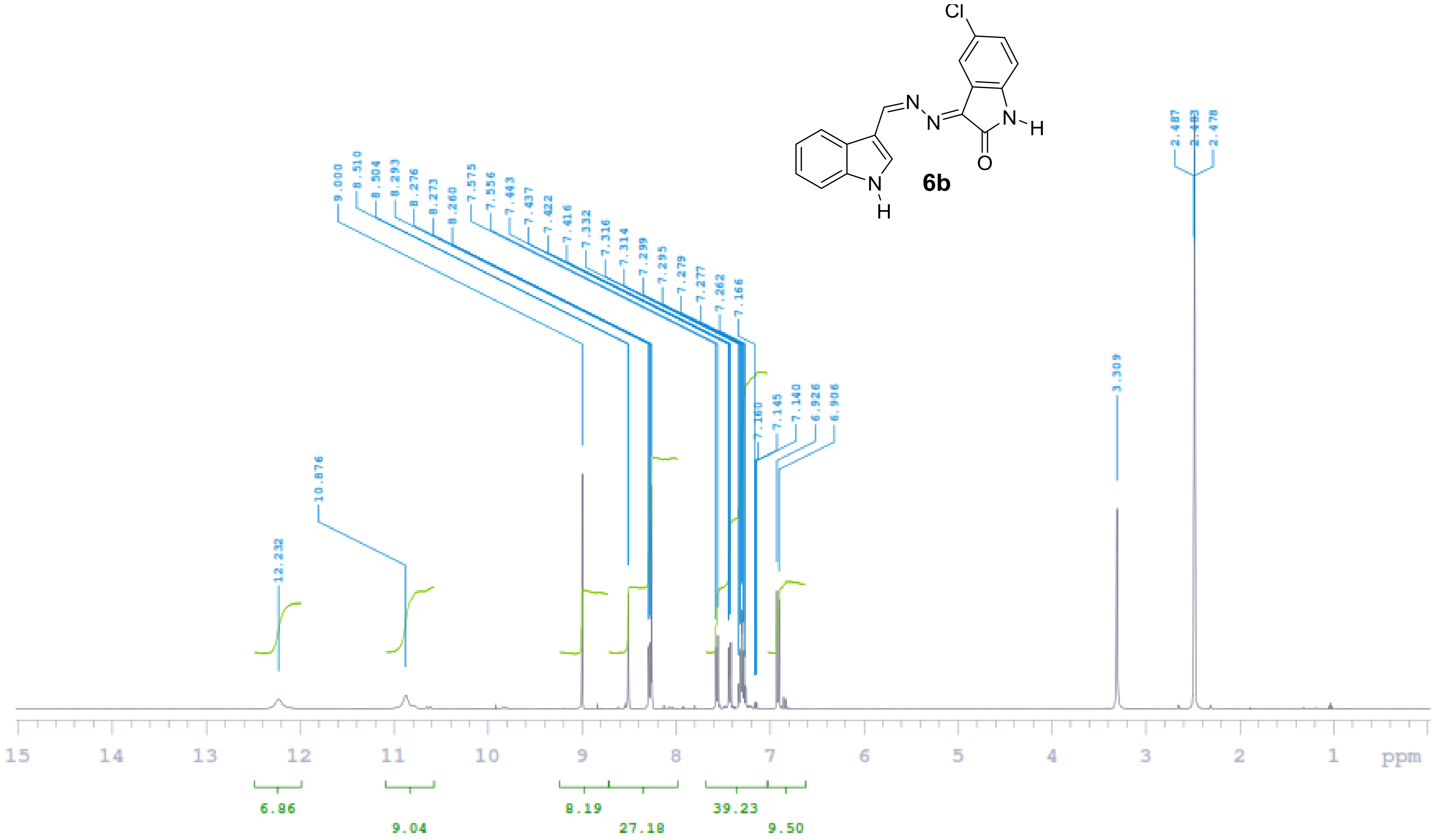

Dr_WagdyMohamed-Z6-B-D2O

Sample Name	Dr_WagdyMohamed-Z6-B-D2O	Pulse sequence	PROTON	Temperature	25	Study owner	vnmr1
Date collected	2016-07-18	Solvent	dmso	Spectrometer	nmr400-mercury400	Operator	vnmr1

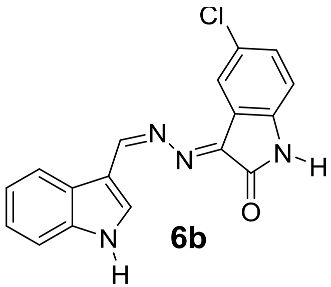

6b

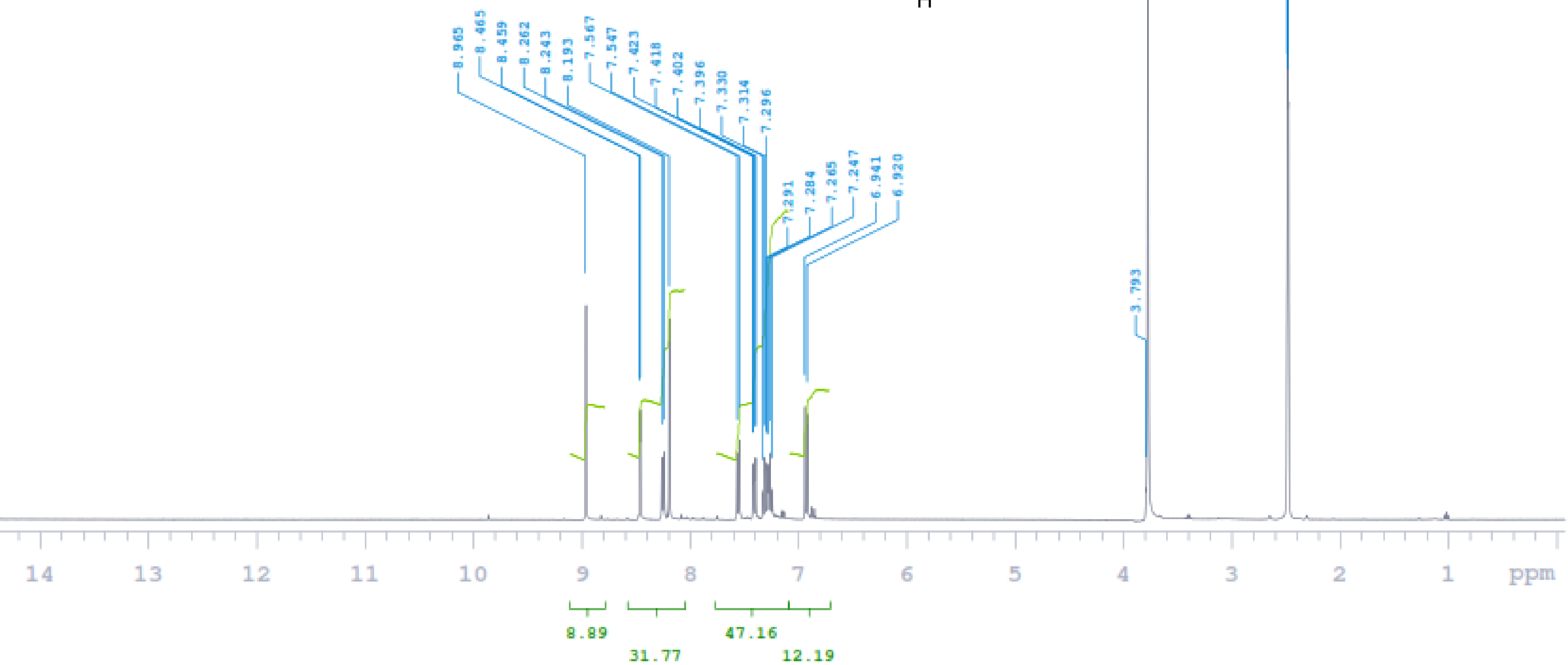

Dr_WagdyMohamed-Z6-B

Sample Name	Dr_WagdyMohamed-Z6-B	Pulse sequence	CARBON	Temperature	25	Study owner	vnmr1
Date collected	2016-12-14	Solvent	dmso	Spectrometer	nmr400-mercury400	Operator	vnmr1

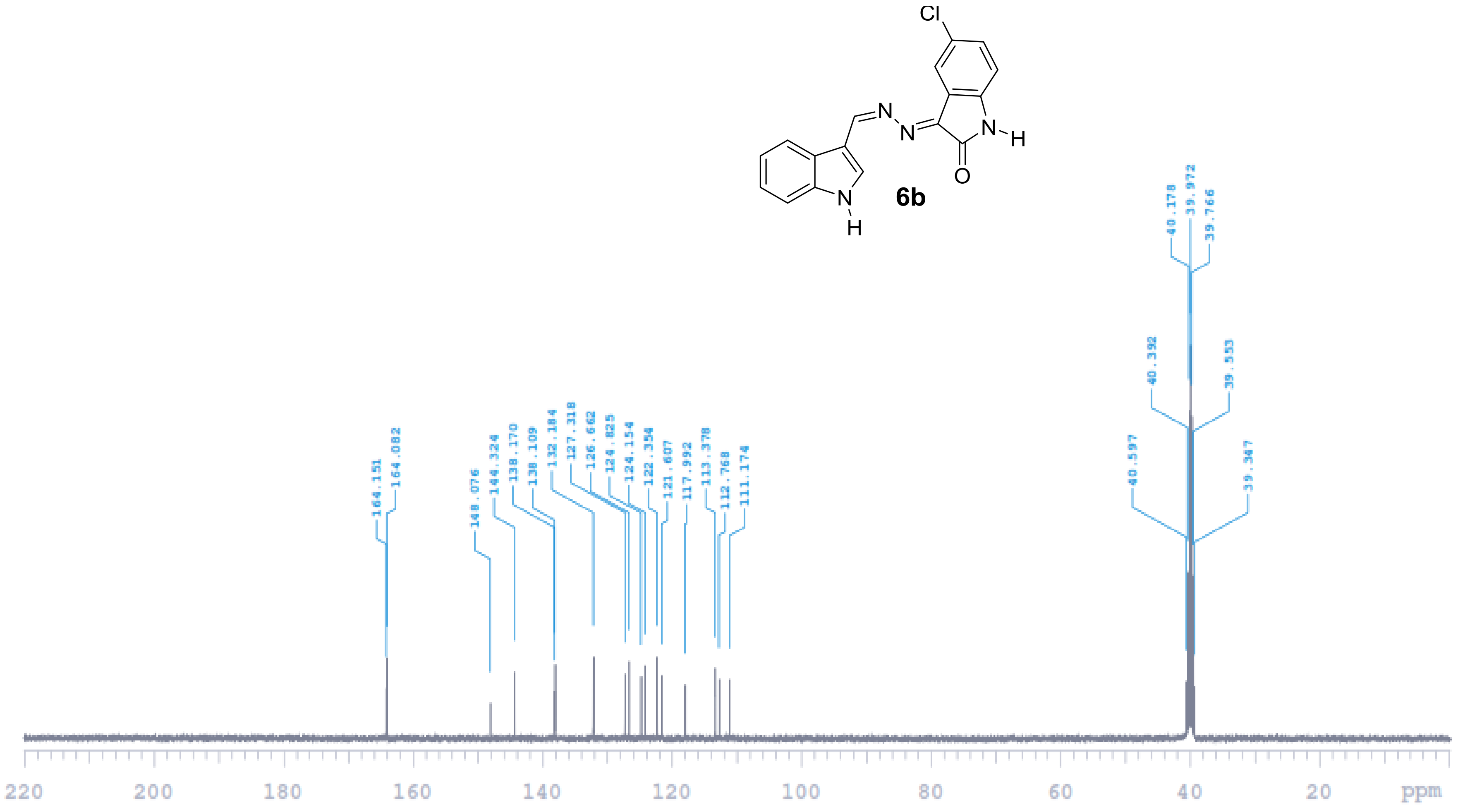

Dr_WagdyMohamed-Z6-C

Sample Name	Dr_WagdyMohamed-Z6-C	Pulse sequence	CARBON	Temperature	25	Study owner	vnmr1
Date collected	2016-12-14	Solvent	dmso	Spectrometer	nmr400-mercury400	Operator	vnmr1

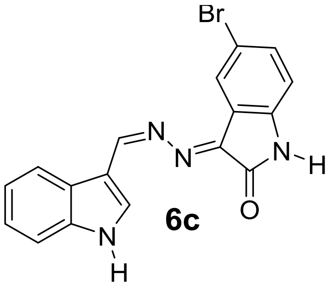

6c

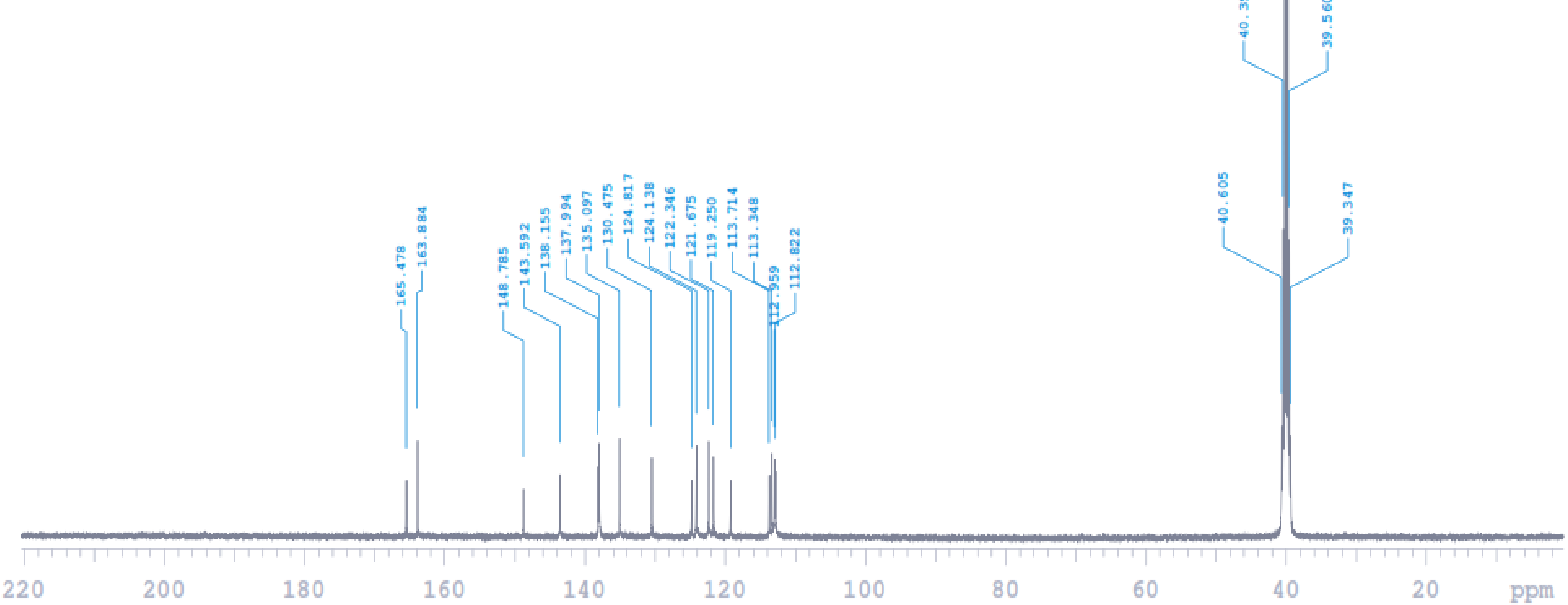

Dr_WagdyMohamed-Z6-D

Sample Name	Dr_WagdyMohamed-Z6-D	Pulse sequence	PROTON	Temperature	25	Study owner	vnmr1
Date collected	2016-08-06	Solvent	dmso	Spectrometer	nmr400-mercury400	Operator	vnmr1

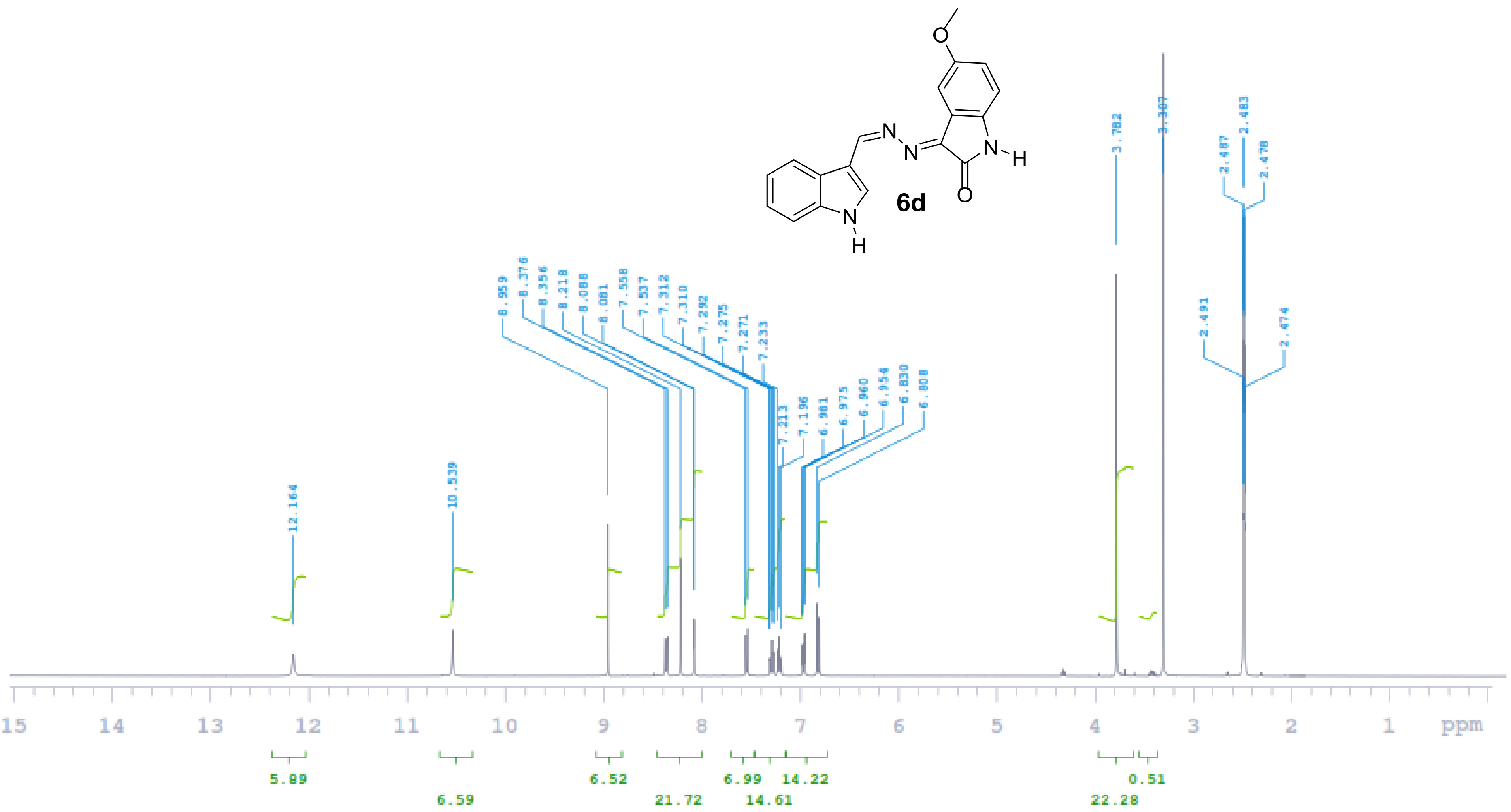

Dr_WagdyMohamed-Z6-D-D2O

Sample Name	Dr_WagdyMohamed-Z6-D-D2O	Pulse sequence	PROTON	Temperature	25	Study owner	vnmr1
Date collected	2016-08-07	Solvent	dmso	Spectrometer	nmr400-mercury400	Operator	vnmr1

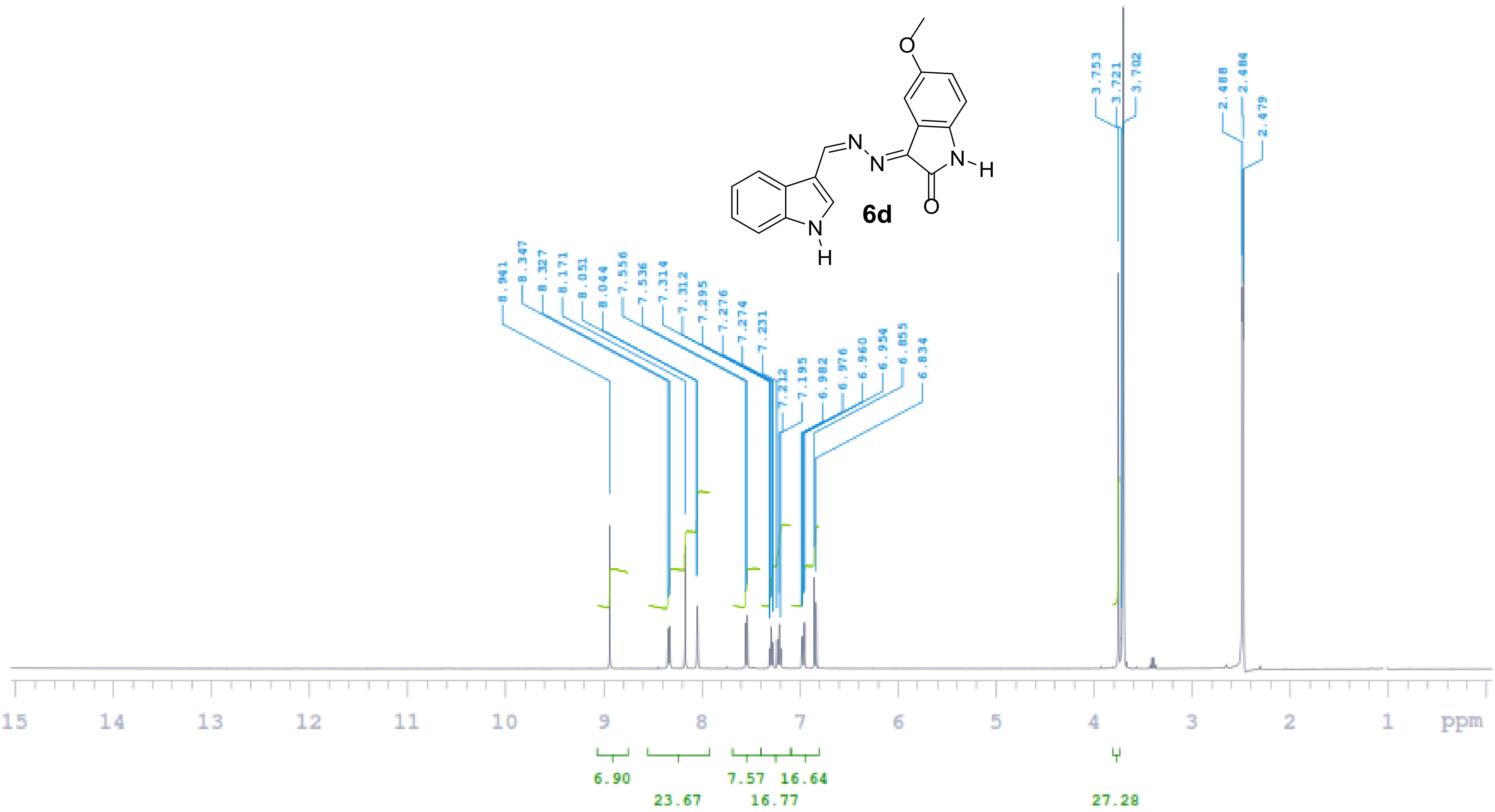

WAGDY(Z6-D)-13C-DMSO-26112017

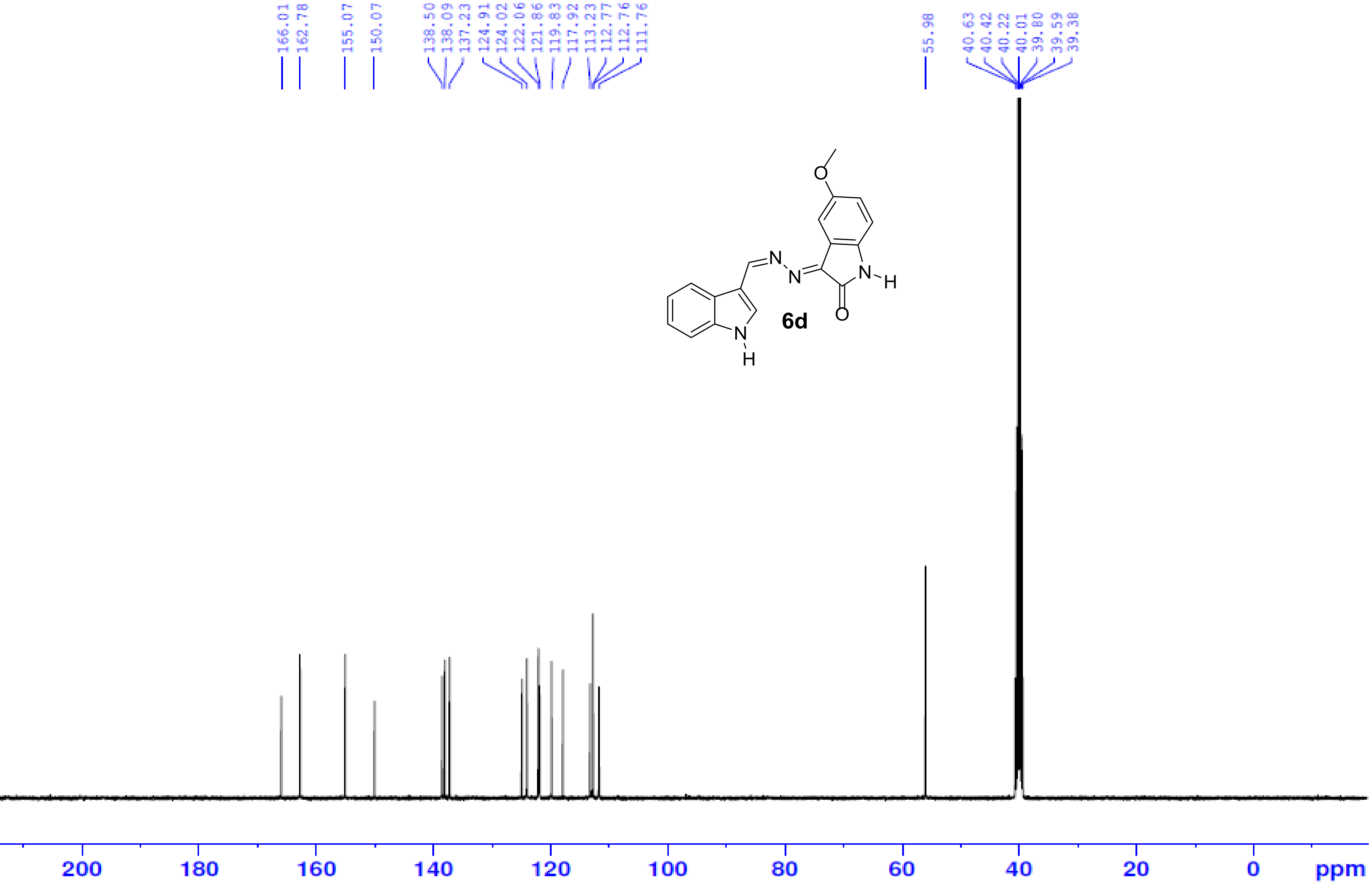

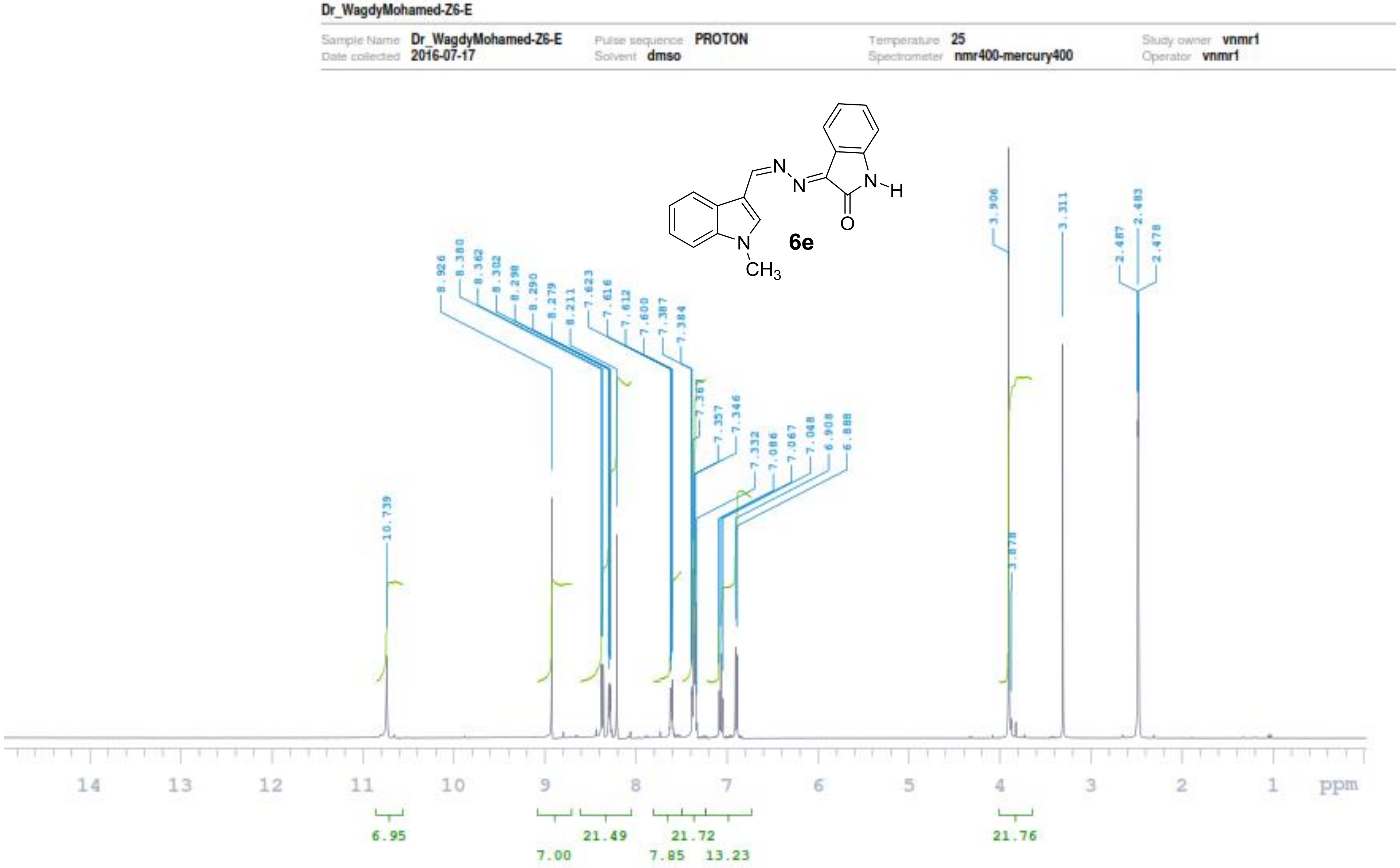
Dr_WagdyMohamed-Z6-E
Sample Name Dr_WagdyMohamed-Z6-E
Date collected 2016-07-17
Pulse sequence PROTON
Solvent dmso
Temperature 25
Spectrometer nmr400-mercury400
Study owner vnmr1
Operator vnmr1
N
N
N
H
O
N
CH3
6e
10.739
8.926
8.380
8.362
8.302
8.298
8.290
8.279
8.211
7.623
7.616
7.612
7.600
7.387
7.384
7.361
7.357
7.346
7.332
7.086
7.067
7.048
6.908
6.888
3.906
3.878
3.311
2.487
2.483
2.478
14 13 12 11 10 9 8 7 6 5 4 3 2 1 ppm
6.95
7.00
21.49
7.85
21.72
13.23
21.76

WAGDY(Z6-E)-13C-DMSO-26112017

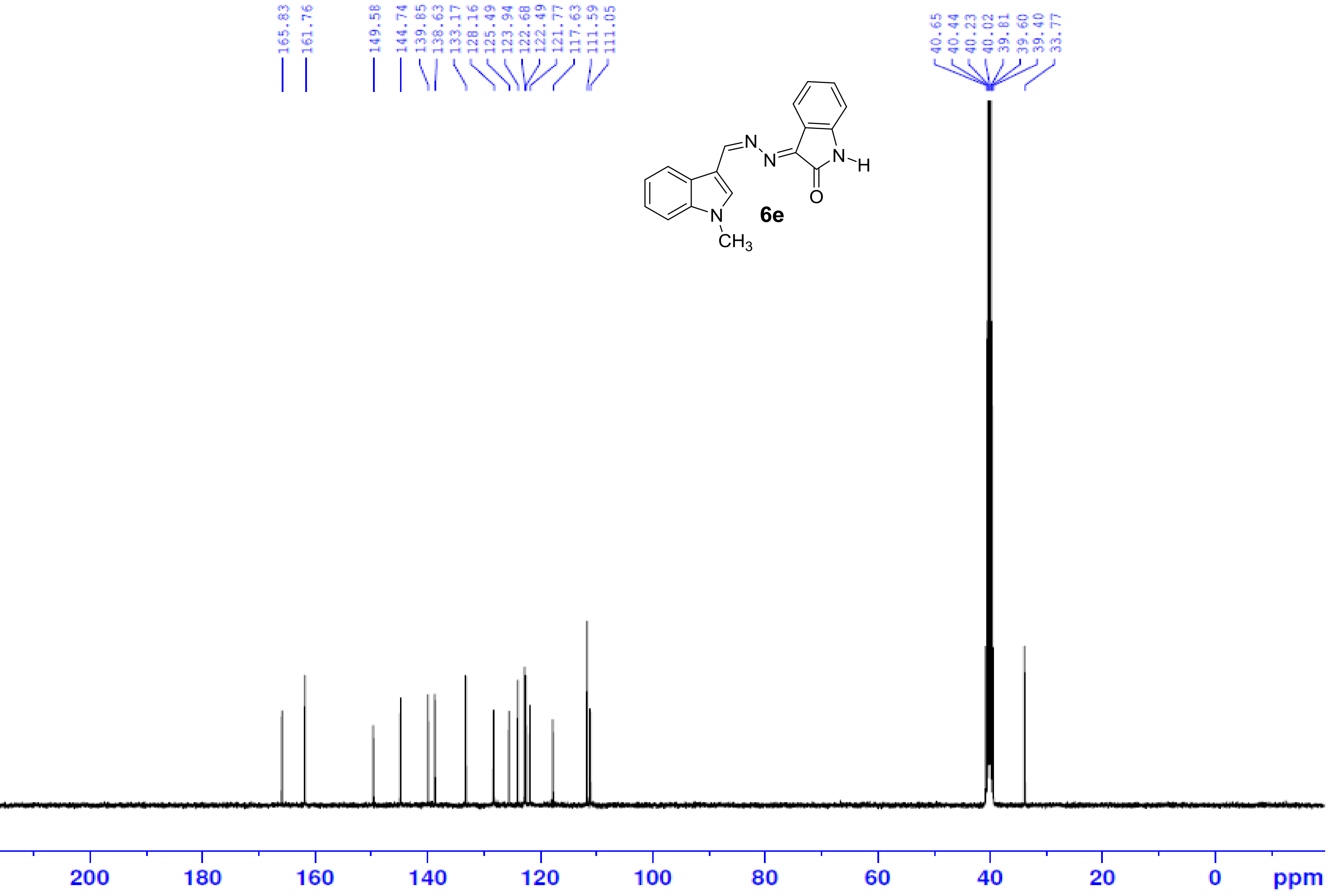

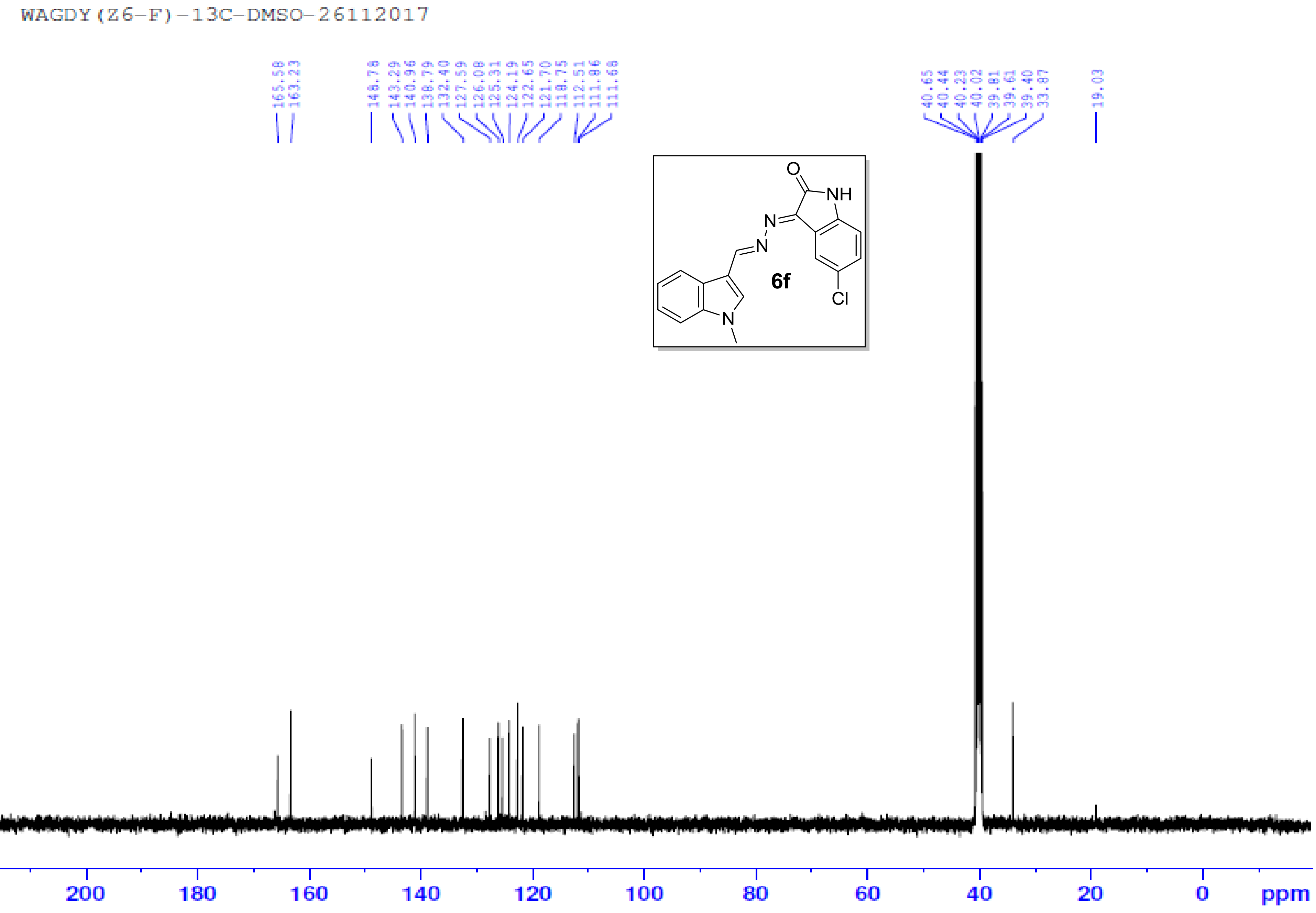
WAGDY(Z6-F)-13C-DMSO-26112017
165.58
163.23
148.78
143.29
140.96
138.79
132.40
127.59
126.08
125.31
124.19
122.65
121.70
118.75
112.51
111.86
111.68
40.65
40.44
40.23
40.02
39.81
39.61
39.40
33.87
19.03
O
NH
N
N
6f
Cl
N
200
180
160
140
120
100
80
60
40
20
0
ppm

Agilent Technologies

Dr_wagdy_mohamed-Z6-G

Sample Name	Dr_wagdy_mohamed-Z6-G	Pulse sequence	CARBON	Temperature	25	Study owner	vnmr1
Date collected	2016-12-01	Solvent	dmso	Spectrometer	nmr400-mercury400	Operator	vnmr1

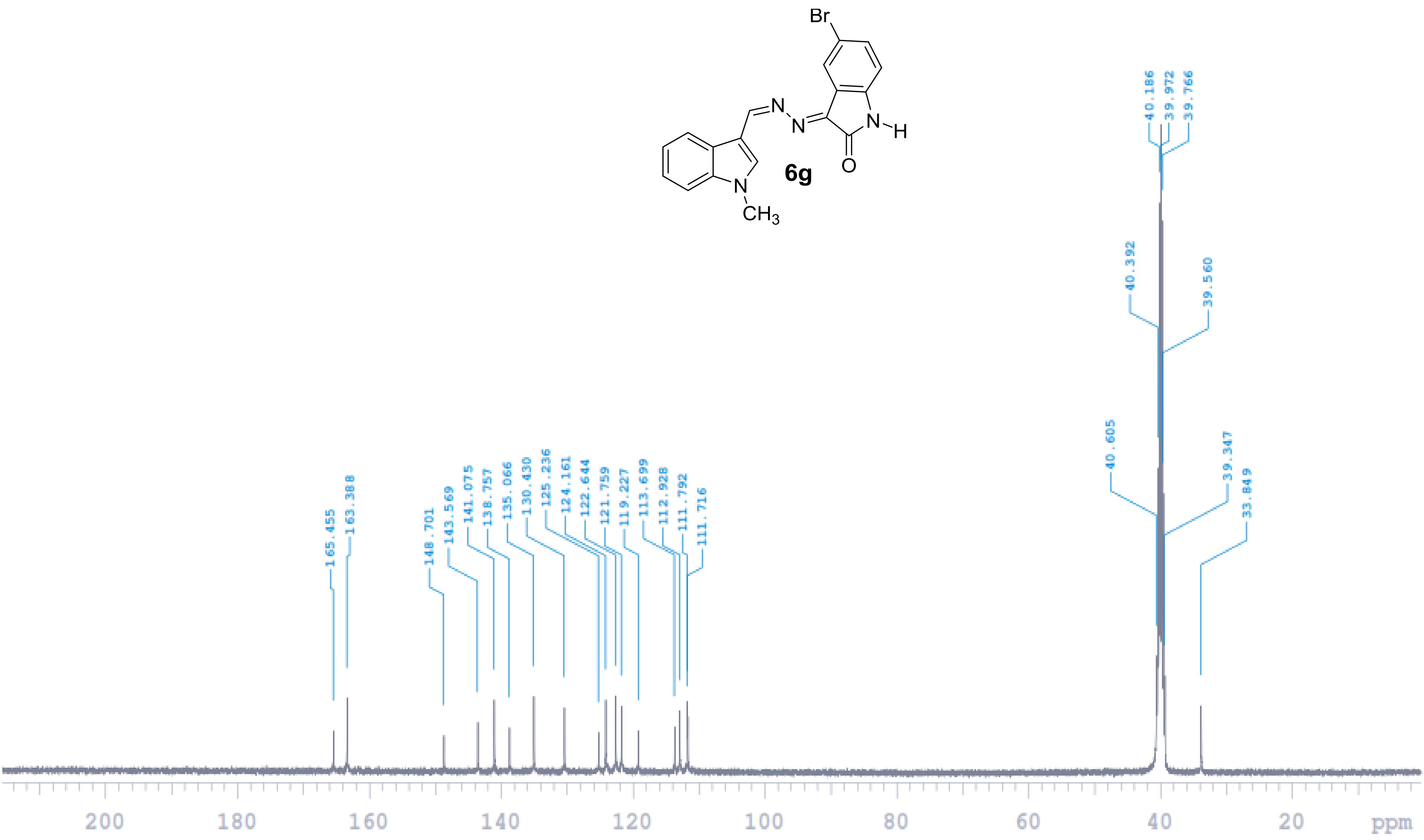

Agilent Technologies

Dr_WagdyMohamed-Z6-H

Sample Name	Dr_WagdyMohamed-Z6-H	Pulse sequence	CARBON	Temperature	25	Study owner	vnmr1
Date collected	2016-11-30	Solvent	dmso	Spectrometer	nmr400-mercury400	Operator	vnmr1

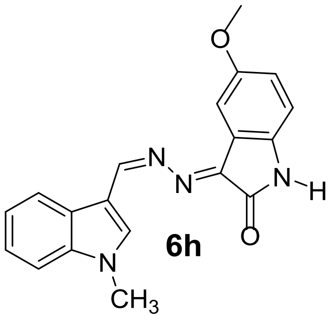

6h

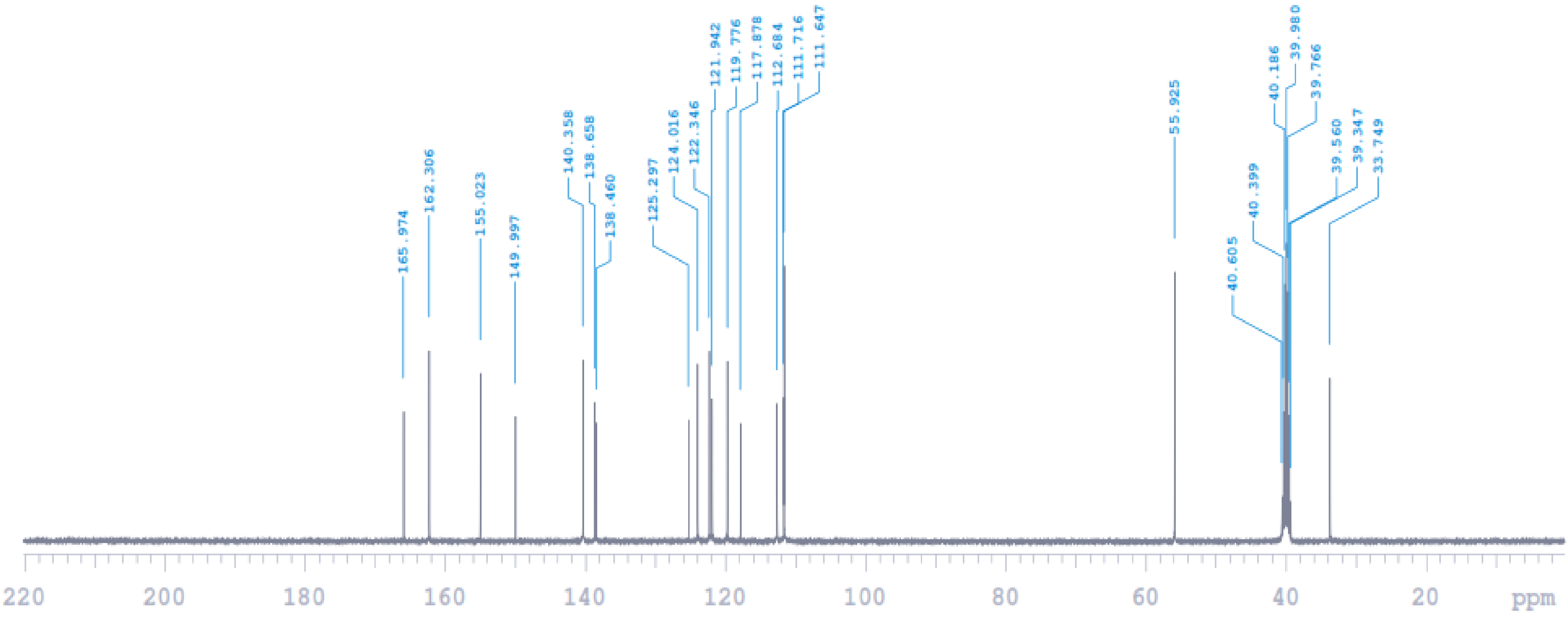

Agilent Technologies

Dr_WagdyMohamed-Z6-K

Sample Name	Dr_WagdyMohamed-Z6-K	Pulse sequence	CARBON	Temperature	25	Study owner	vnmr1
Date collected	2016-12-12	Solvent	dmso	Spectrometer	nmr400-mercury400	Operator	vnmr1

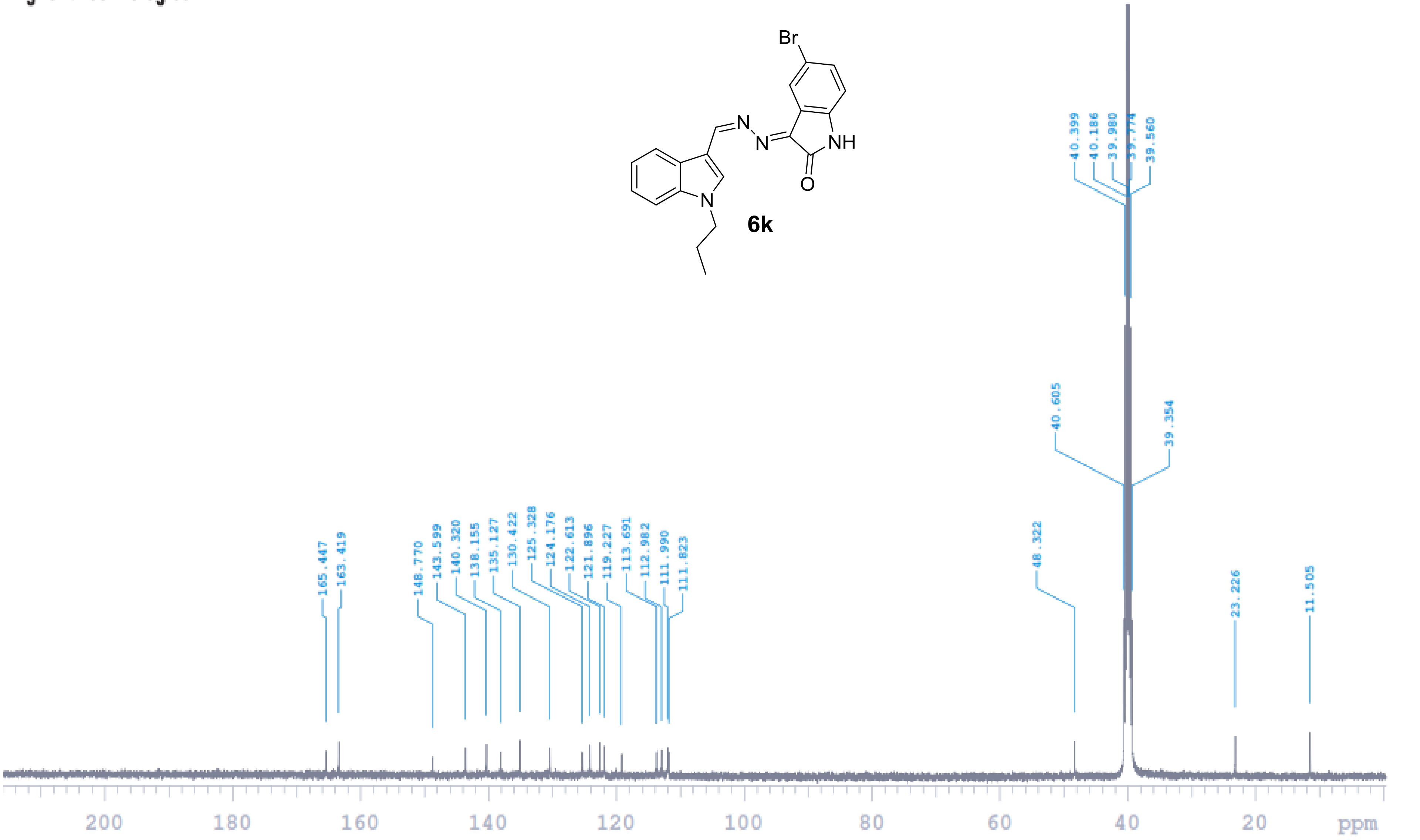

Dr_WagdyMohamed-3

Sample Name	**Dr_WagdyMohamed-3**	Pulse sequence	**PROTON**	Temperature	**25**	Study owner	**vnmr1**
Date collected	**2016-08-22**	Solvent	**dmso**	Spectrometer	**nmr400-mercury400**	Operator	**vnmr1**

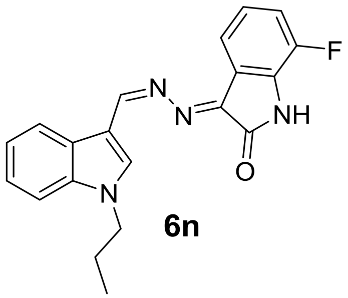

6n

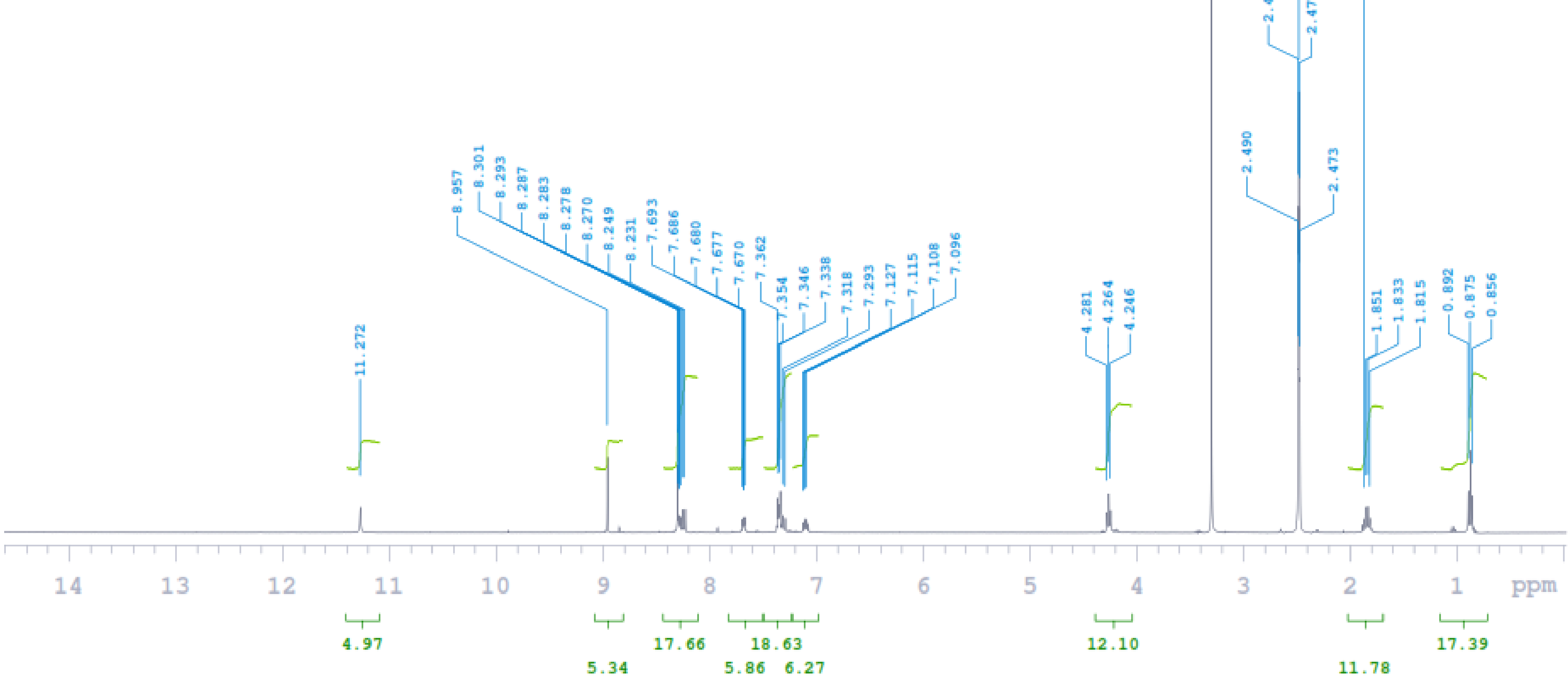

Dr_WagdyMohamed-Z6-M

Sample Name	Dr_WagdyMohamed-Z6-M	Pulse sequence	PROTON	Temperature	25	Study owner	vnmr1
Date collected	2016-08-06	Solvent	dmso	Spectrometer	nmr400-mercury400	Operator	vnmr1

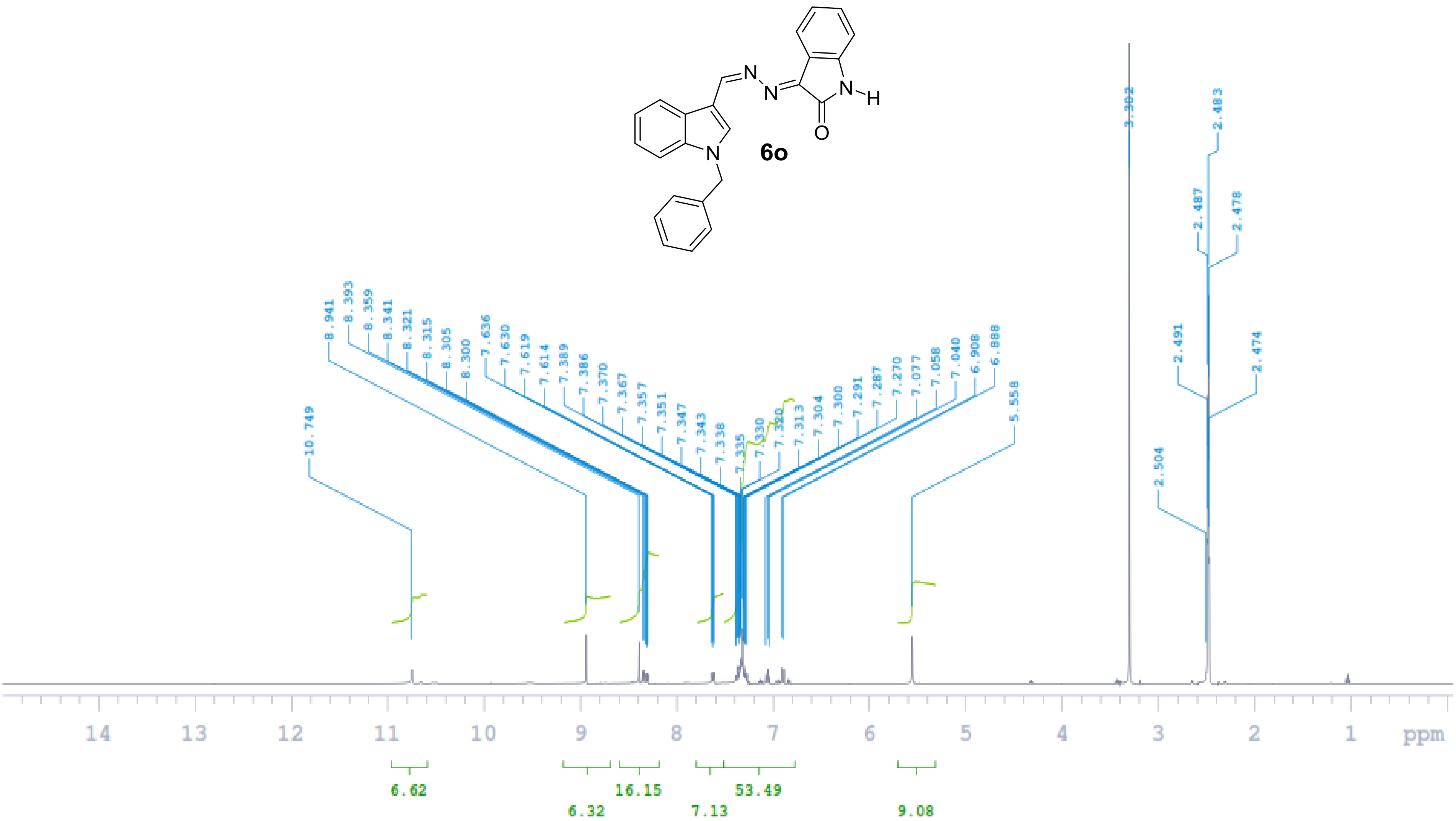

Dr_WagdyMohamed-Z6-M

Dr_WagdyMohamed-Z6-M

Sample Name	Dr_WagdyMohamed-Z6-M	Pulse sequence	CARBON	Temperature	25	Study owner	vnmr1
Date collected	2016-12-13	Solvent	dmso	Spectrometer	nmr400-mercury400	Operator	vnmr1

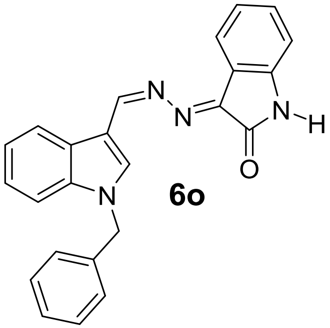

6o

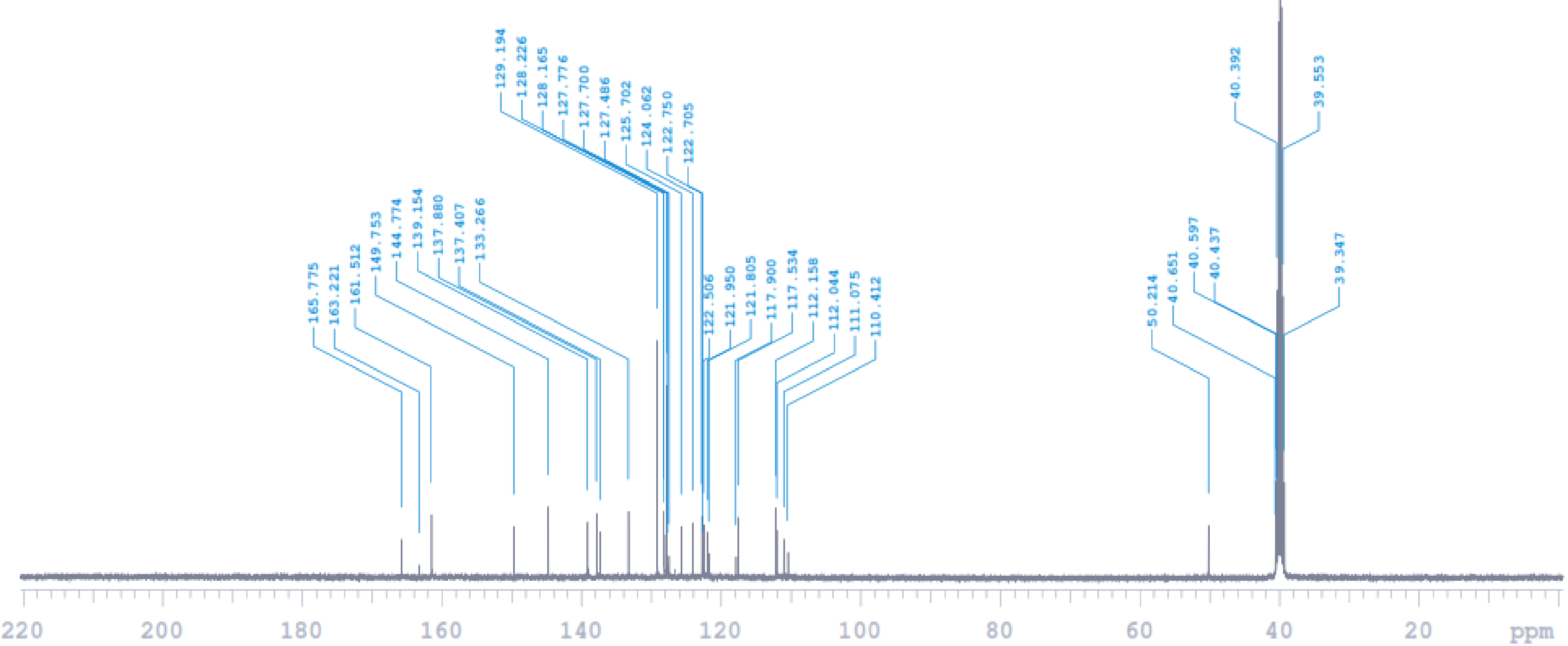

Sample Name	Dr_WagdyMohamed-Z6-N-D2O	Pulse sequence	PROTON	Temperature	25	Study owner	vnmr1
Date collected	2016-08-07	Solvent	dmso	Spectrometer	nmr400-mercury400	Operator	vnmr1

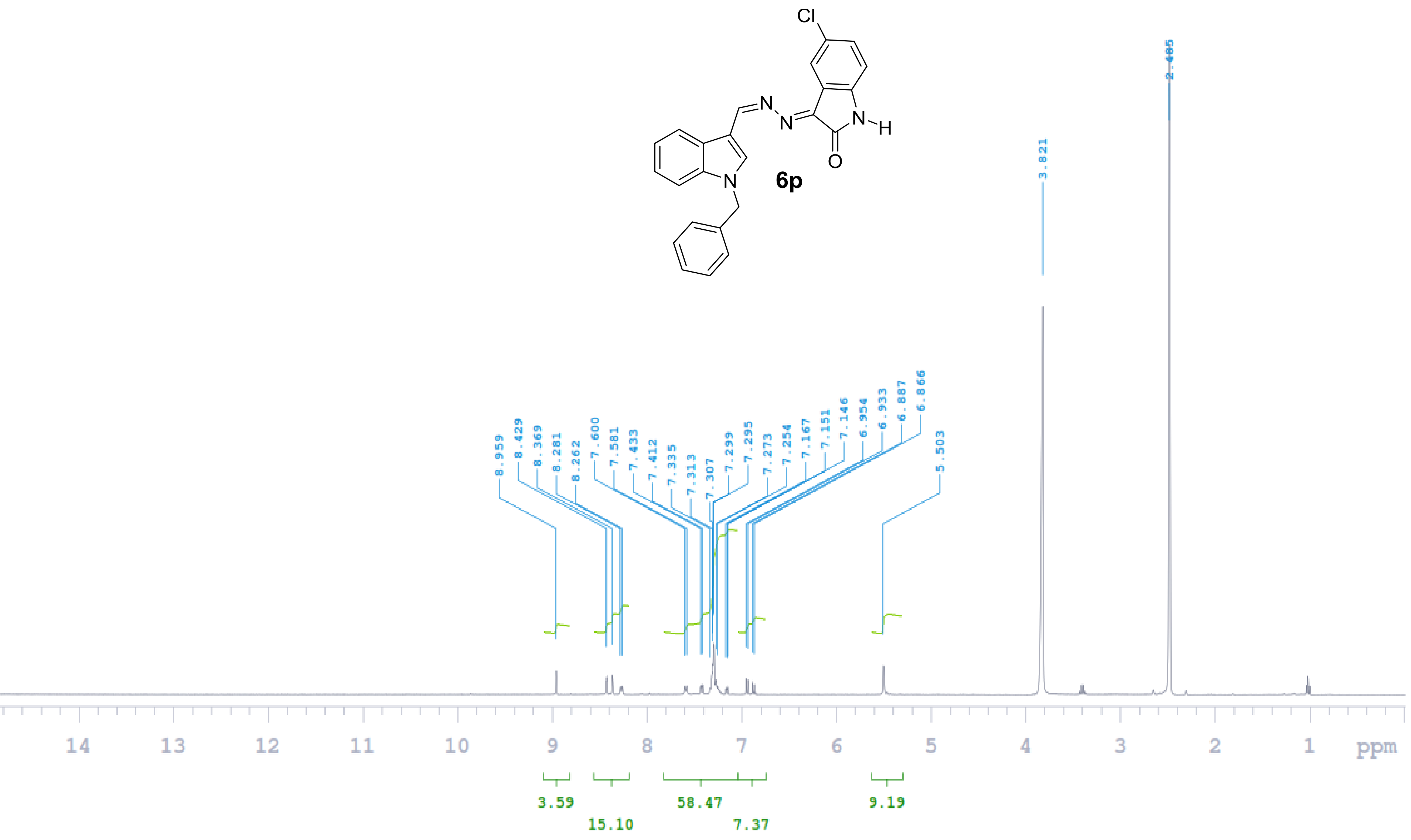

WAGDY(6P)-13C-DMSO-26112017

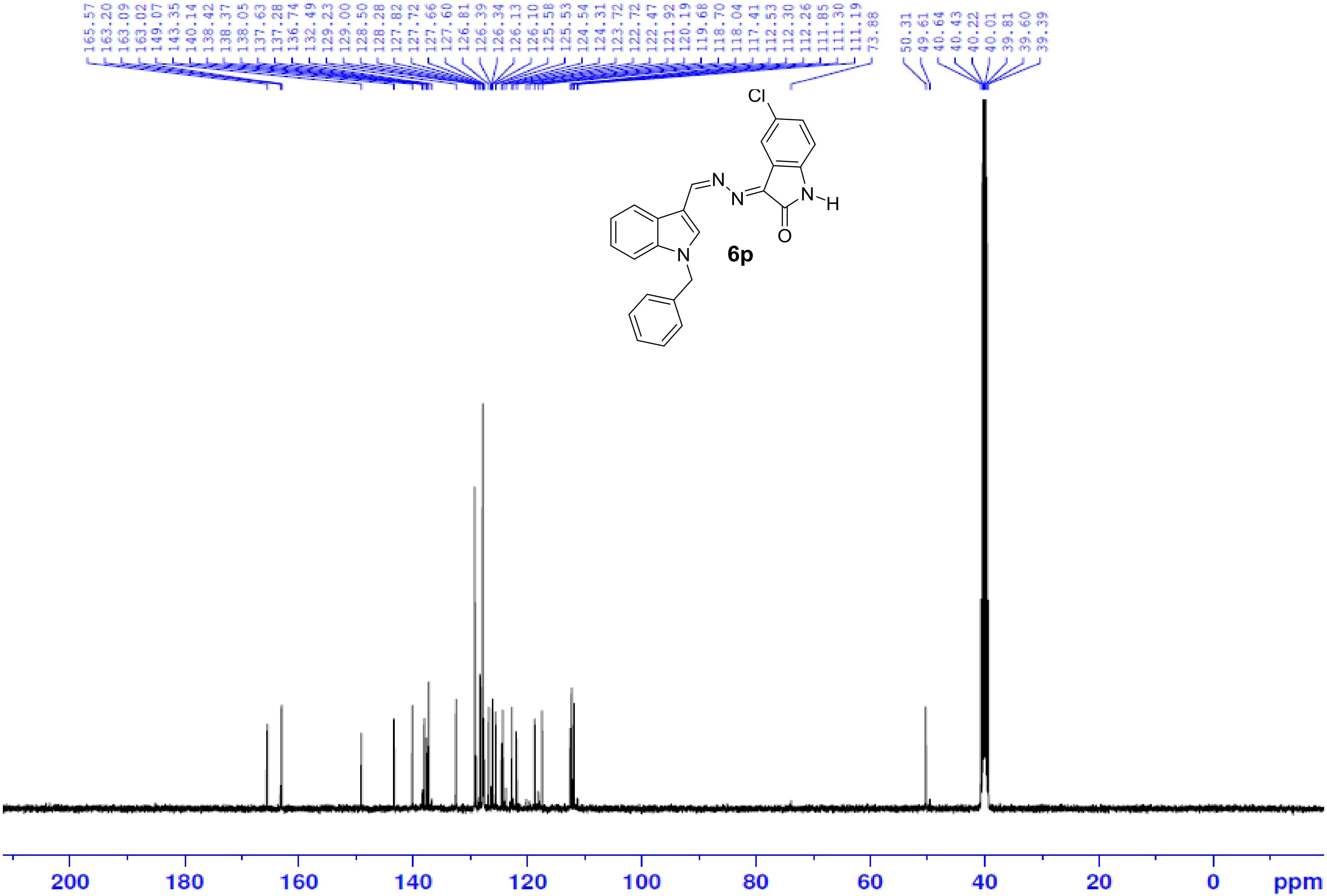

Sample Name **Dr_WagdyMohamed-Z6-P** Pulse sequence **PROTON** Temperature **25** Study owner **vnmr1**
Date collected **2016-07-17** Solvent **dmso** Spectrometer **nmr400-mercury400** Operator **vnmr1**

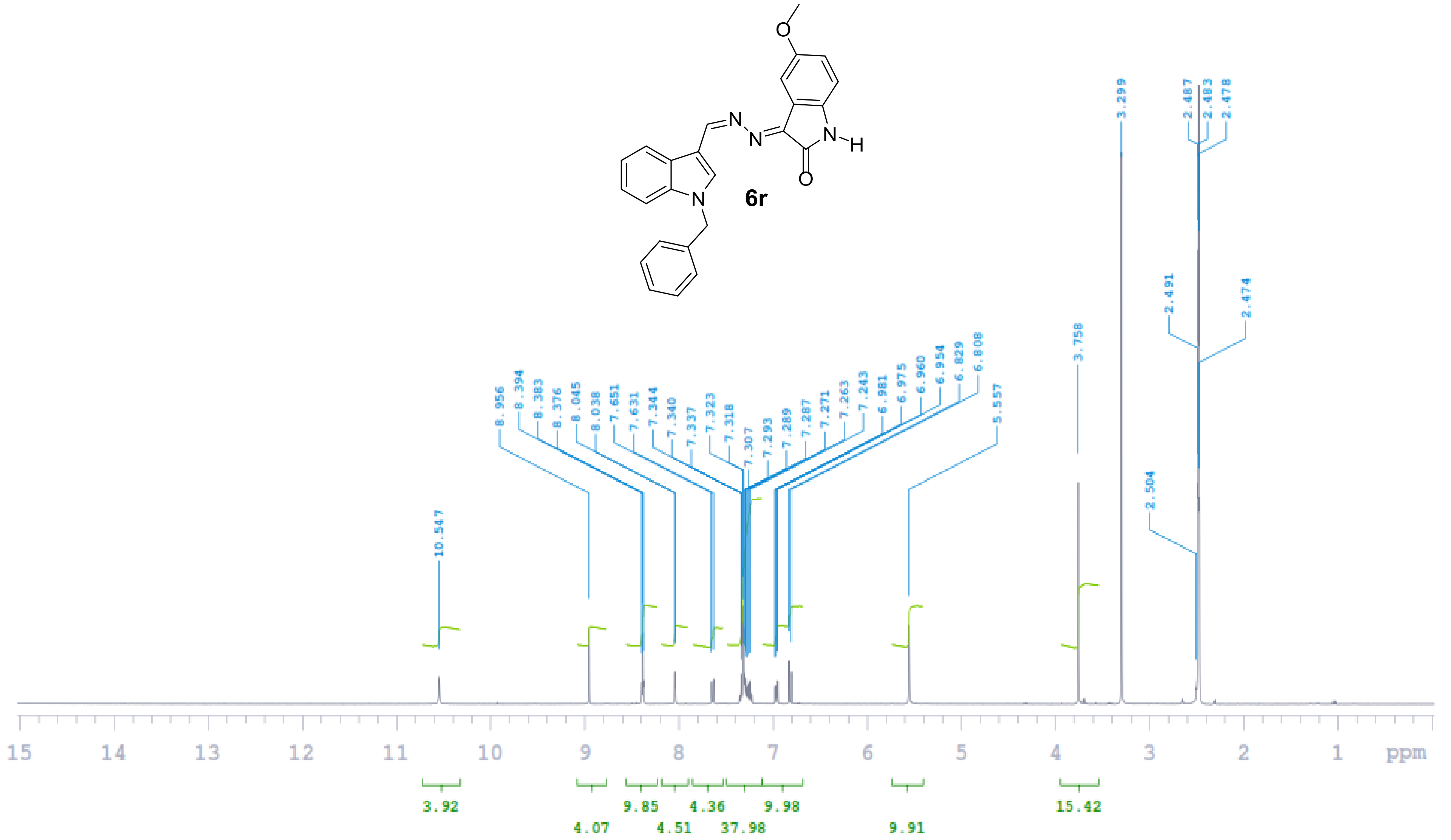

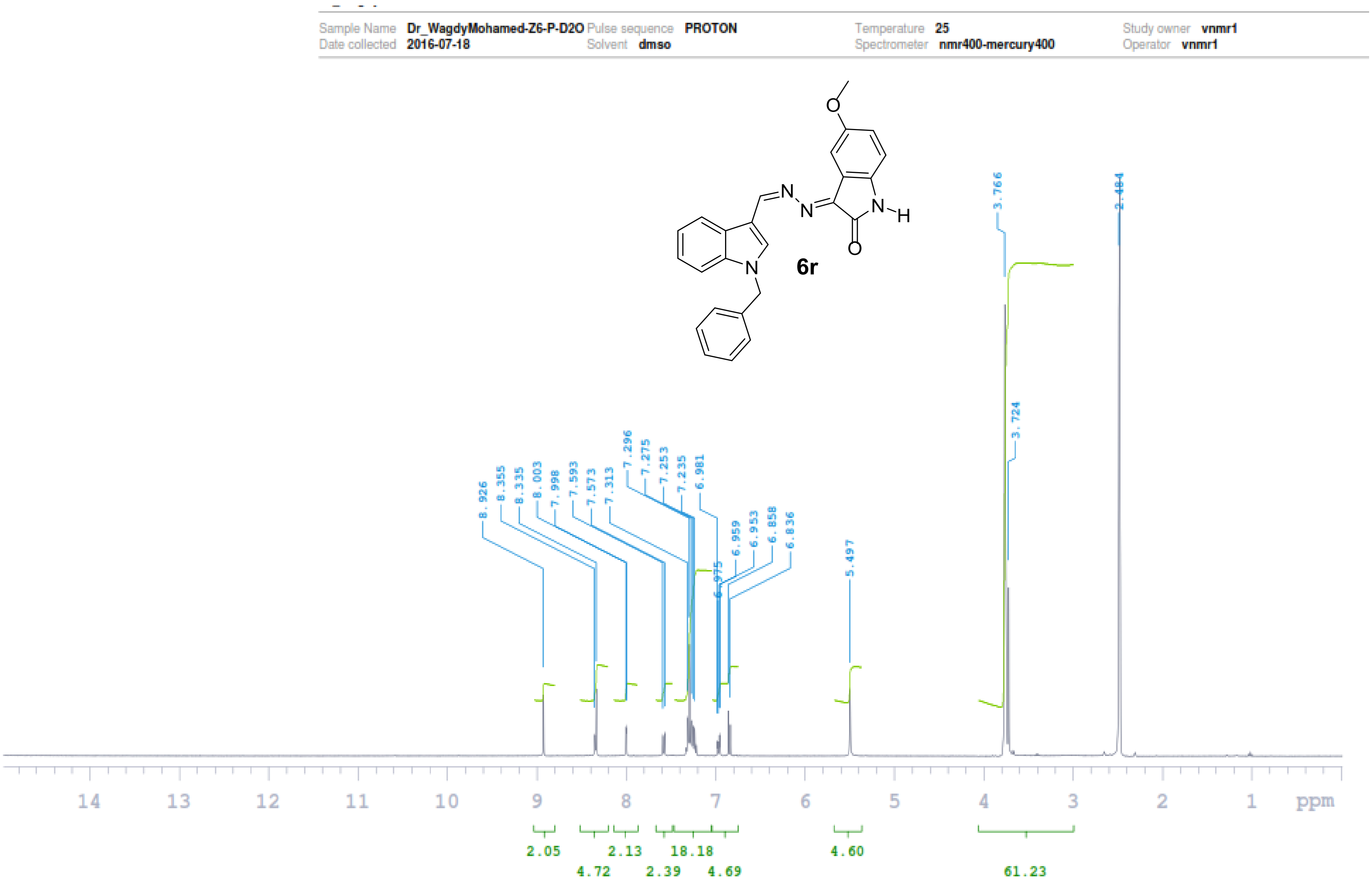
Sample Name Dr_WagdyMohamed-Z6-P-D2O
Date collected 2016-07-18
Pulse sequence PROTON
Solvent dmso
Temperature 25
Spectrometer nmr400-mercury400
Study owner vnmr1
Operator vnmr1
6r
8.926 8.355 8.335 8.003 7.998 7.593 7.573 7.313 7.296 7.275 7.253 7.235 6.981 6.975 6.959 6.953 6.858 6.836 5.497 3.766 3.724 2.484
2.05 4.72 2.13 2.39 18.18 4.69 4.60 61.23
14 13 12 11 10 9 8 7 6 5 4 3 2 1 ppm

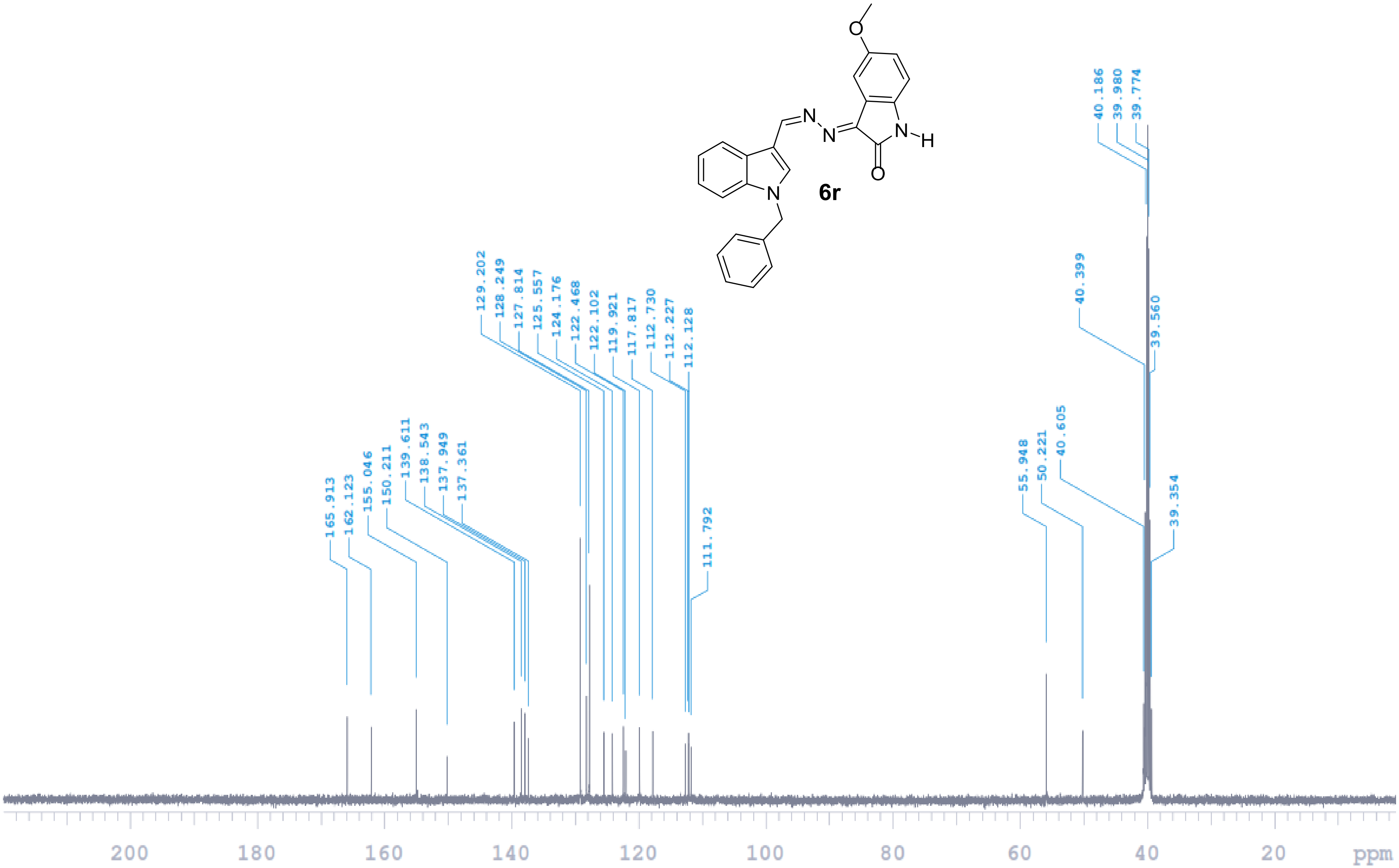
6r
165.913
162.123
155.046
150.211
139.611
138.543
137.949
137.361
129.202
128.249
127.814
125.557
124.176
122.468
122.102
119.921
117.817
112.730
112.227
112.128
111.792
55.948
50.221
40.605
40.399
40.186
39.980
39.774
39.560
39.354
200
180
160
140
120
100
80
60
40
20
ppm

Dr_WagdyMohamed-N-Me-CL

Sample Name	Dr_WagdyMohamed-N-Me-CL	Pulse sequence	PROTON	Temperature	25	Study owner	vnmr1
Date collected	2016-11-17	Solvent	dmso	Spectrometer	nmr400-mercury400	Operator	vnmr1

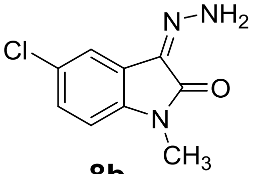

8b

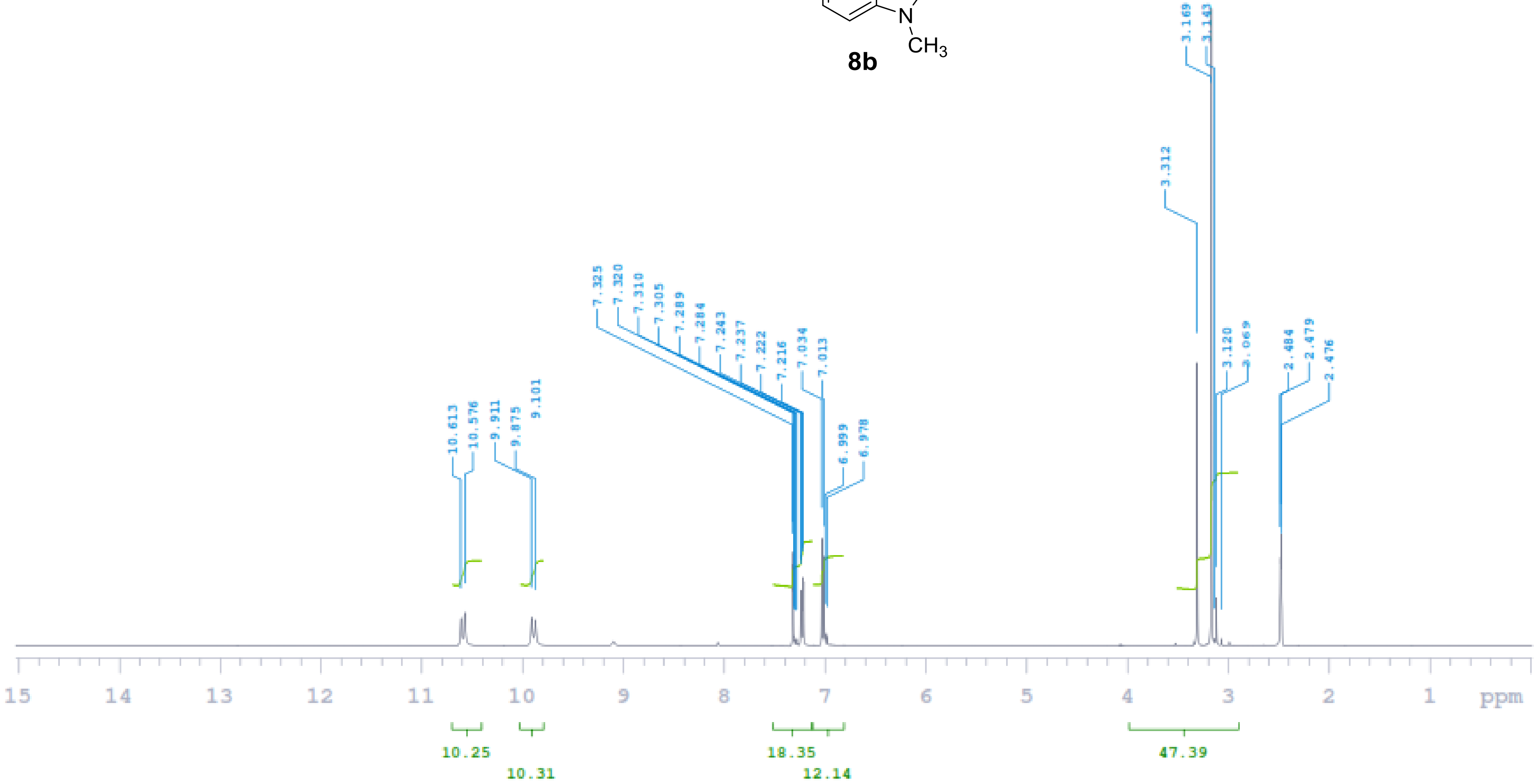

Sample Name	Dr_WagdyMohamed-N-Me-CL-D2O	Pulse sequence	PROTON	Temperature	25	Study owner	vnmr1
Date collected	2016-12-01	Solvent	dmso	Spectrometer	nmr400-mercury400	Operator	vnmr1

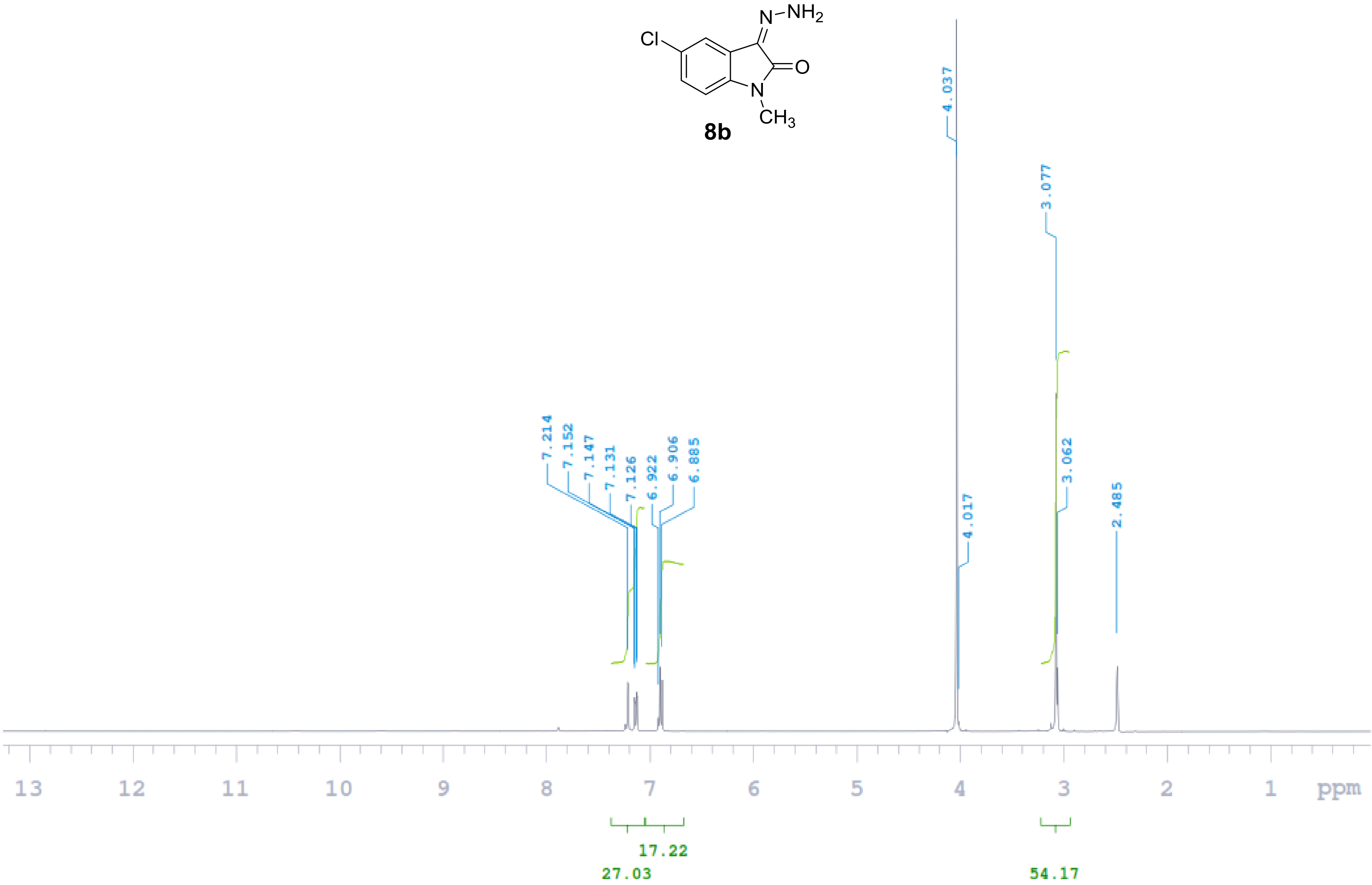

Dr_WagdyMohamed-N-Me-CL

Agilent Technologies

Sample Name	Dr_WagdyMohamed-N-Me-CL	Pulse sequence	CARBON	Temperature	25	Study owner	vnmr1
Date collected	2016-11-29	Solvent	dmso	Spectrometer	nmr400-mercury400	Operator	vnmr1

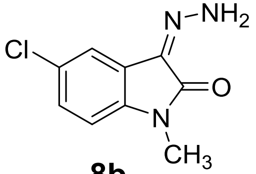

8b

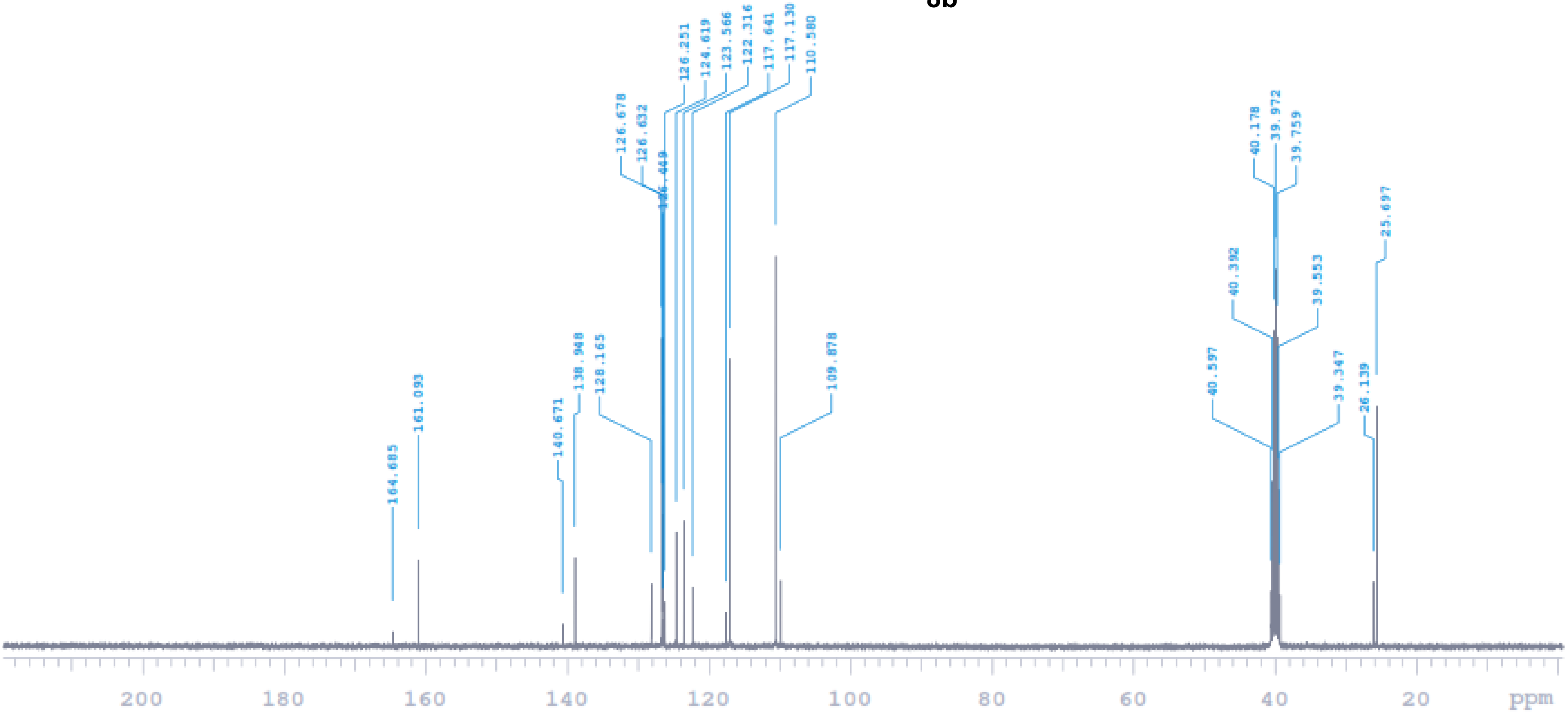

Dr_WagdyMohamed-Z9-A

Sample Name	**Dr_WagdyMohamed-Z9-A**	Pulse sequence	**PROTON**	Temperature	**25**	Study owner	**vnmr1**
Date collected	**2016-07-17**	Solvent	**dmso**	Spectrometer	**nmr400-mercury400**	Operator	**vnmr1**

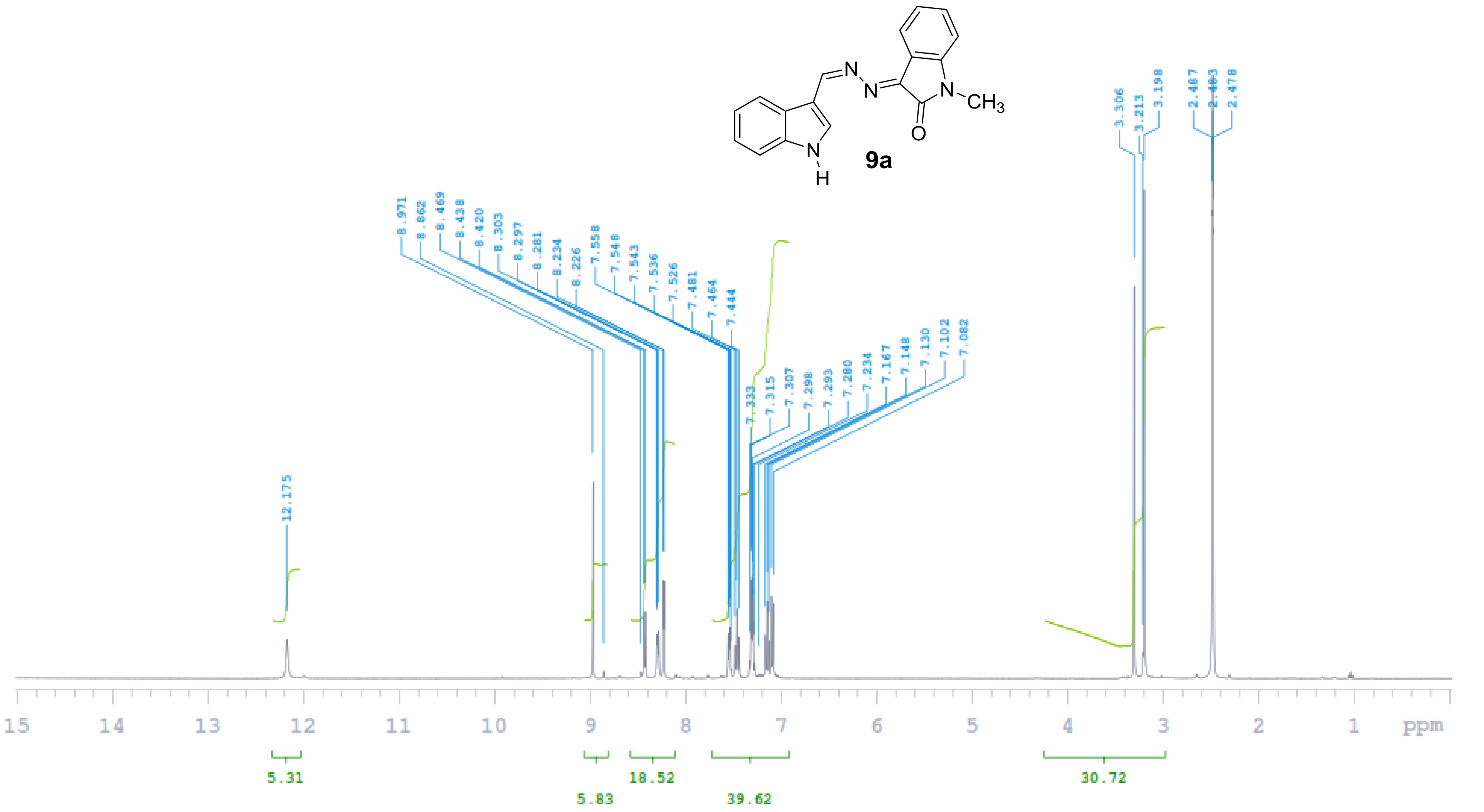

Dr_WagdyMohamed-Z9-A-D2O

Sample Name	Dr_WagdyMohamed-Z9-A-D2O	Pulse sequence	PROTON	Temperature	25	Study owner	vnmr1
Date collected	2016-07-18	Solvent	dmso	Spectrometer	nmr400-mercury400	Operator	vnmr1

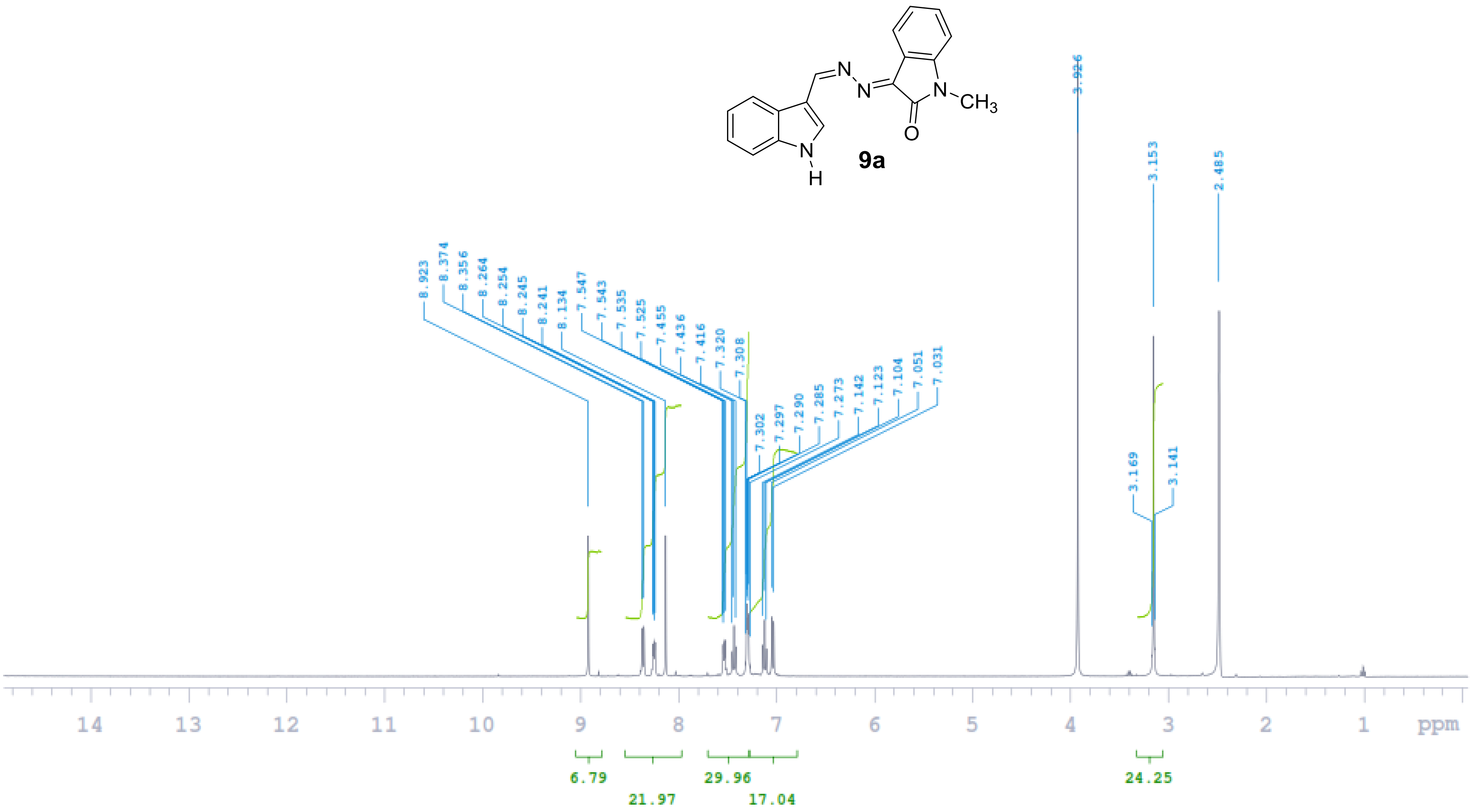

Dr_WagdyMohamed-Z9-A

Agilent Technologies

Sample Name	Dr_WagdyMohamed-Z9-A	Pulse sequence	CARBON	Temperature	25	Study owner	vnmr1
Date collected	2016-12-08	Solvent	dmso	Spectrometer	nmr400-mercury400	Operator	vnmr1

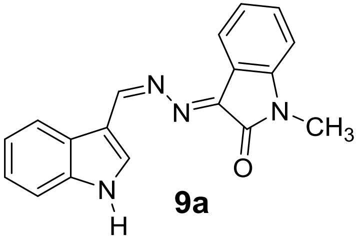

9a

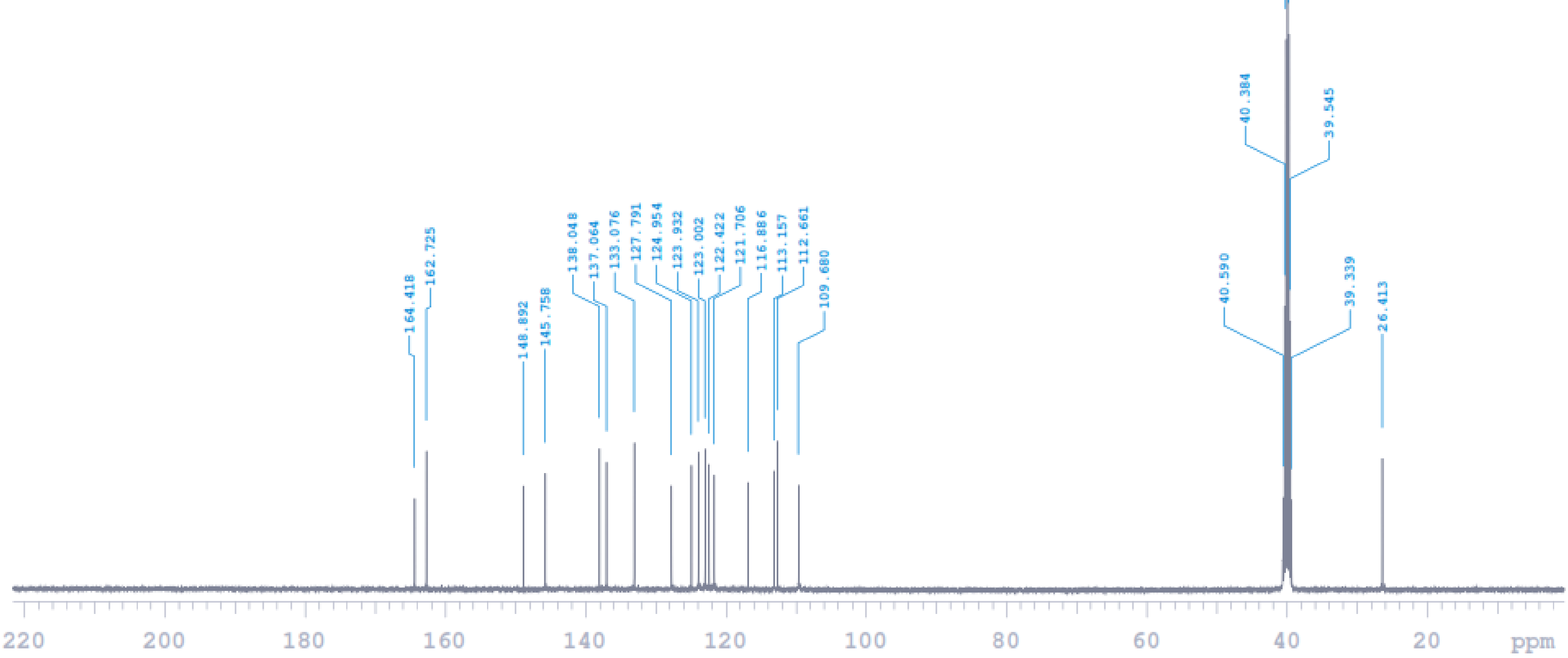

Sample Name **Dr_WagdyMohamed-Z9-B** Pulse sequence **PROTON** Temperature **25** Study owner **vnmr1**
Date collected **2016-08-06** Solvent **dmso** Spectrometer **nmr400-mercury400** Operator **vnmr1**

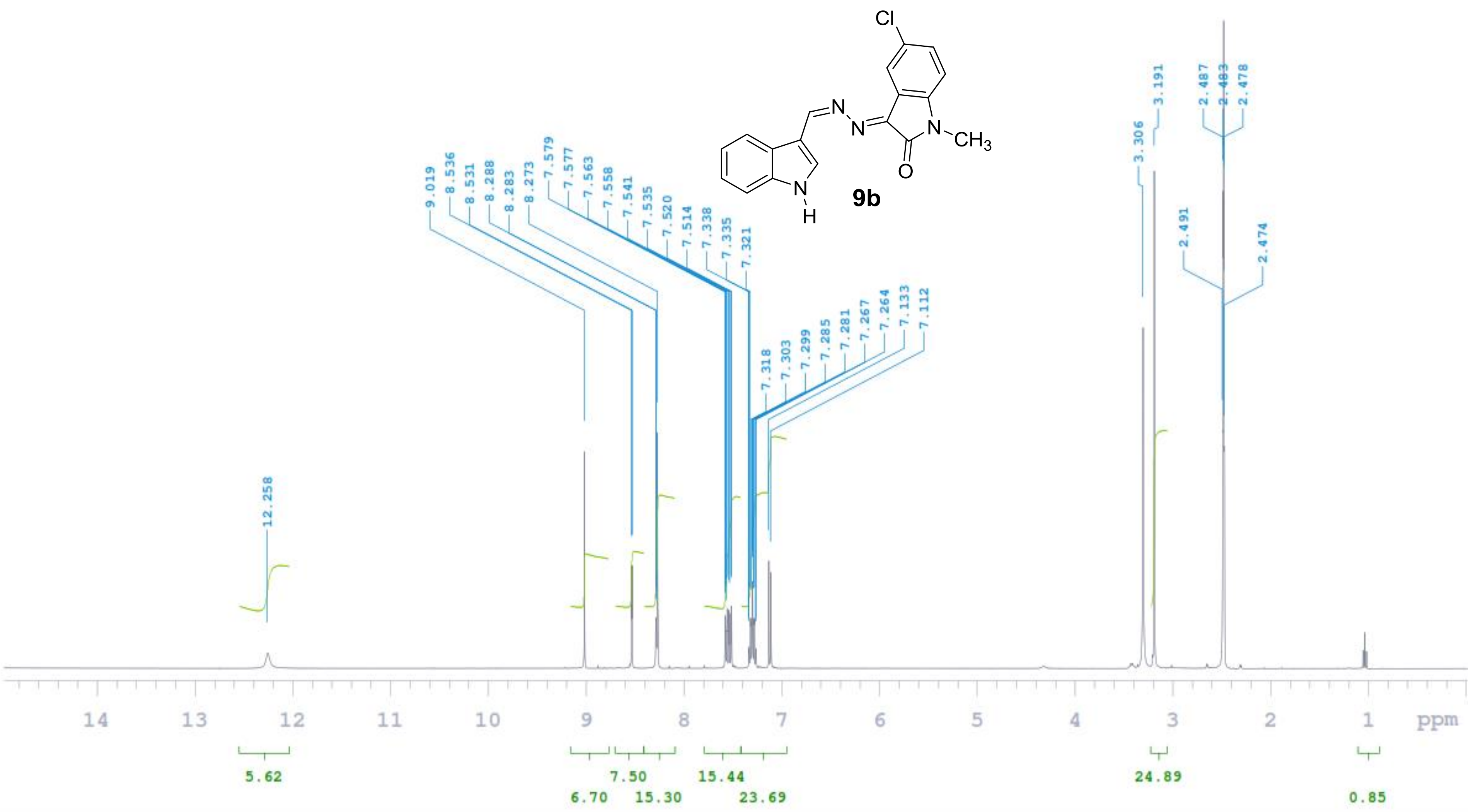

Dr_WagdyMohamed-Z9-C

Sample Name	**Dr_WagdyMohamed-Z9-C**	Pulse sequence	**PROTON**	Temperature	**25**	Study owner	**vnmr1**
Date collected	**2016-08-06**	Solvent	**dmso**	Spectrometer	**nmr400-mercury400**	Operator	**vnmr1**

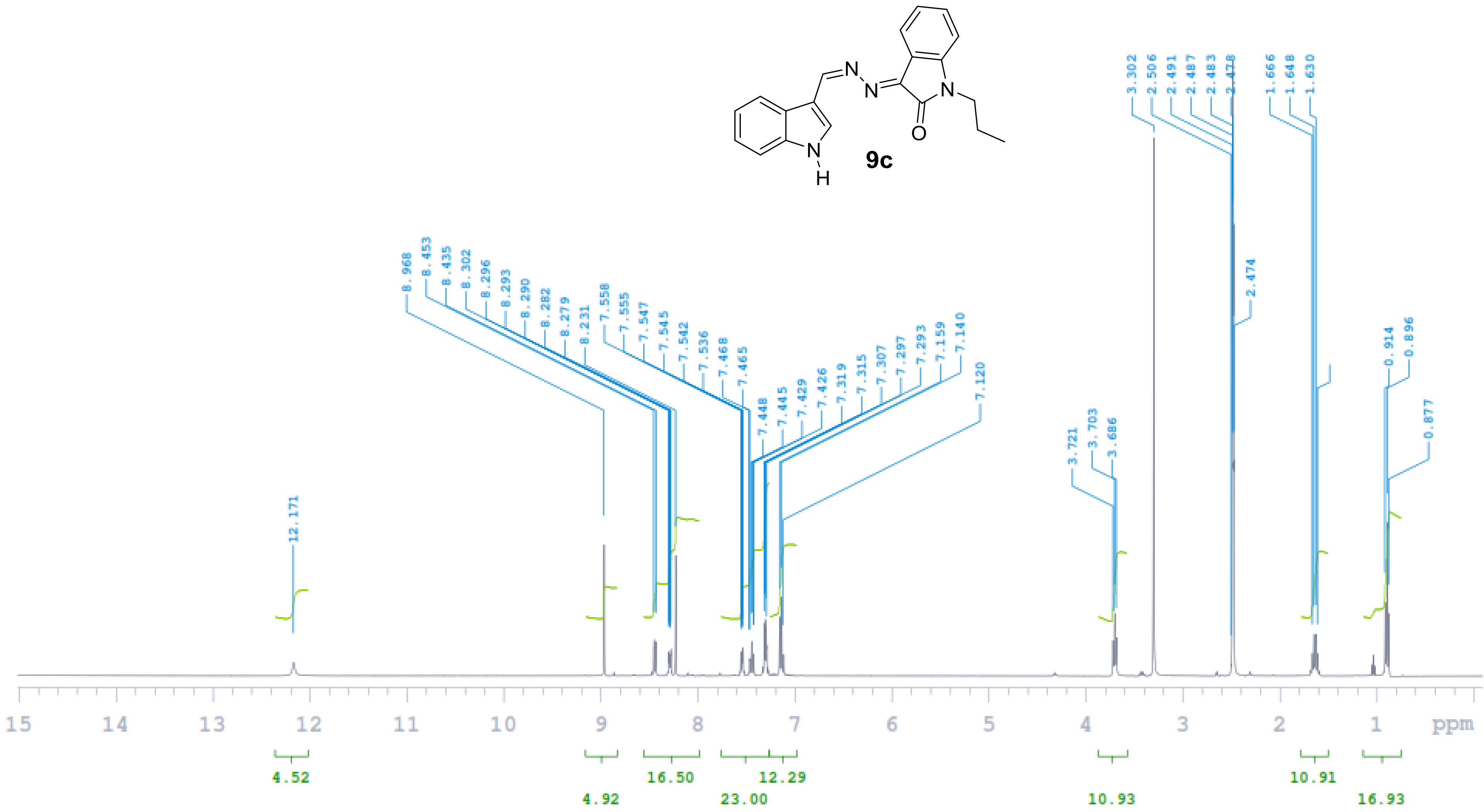

Dr_WagdyMohamed-Z9-C-D2O

Sample Name Dr_WagdyMohamed-Z9-C-D2O	Pulse sequence PROTON	Temperature 25	Study owner vnmr1
Date collected 2016-08-07	Solvent dmso	Spectrometer nmr400-mercury400	Operator vnmr1

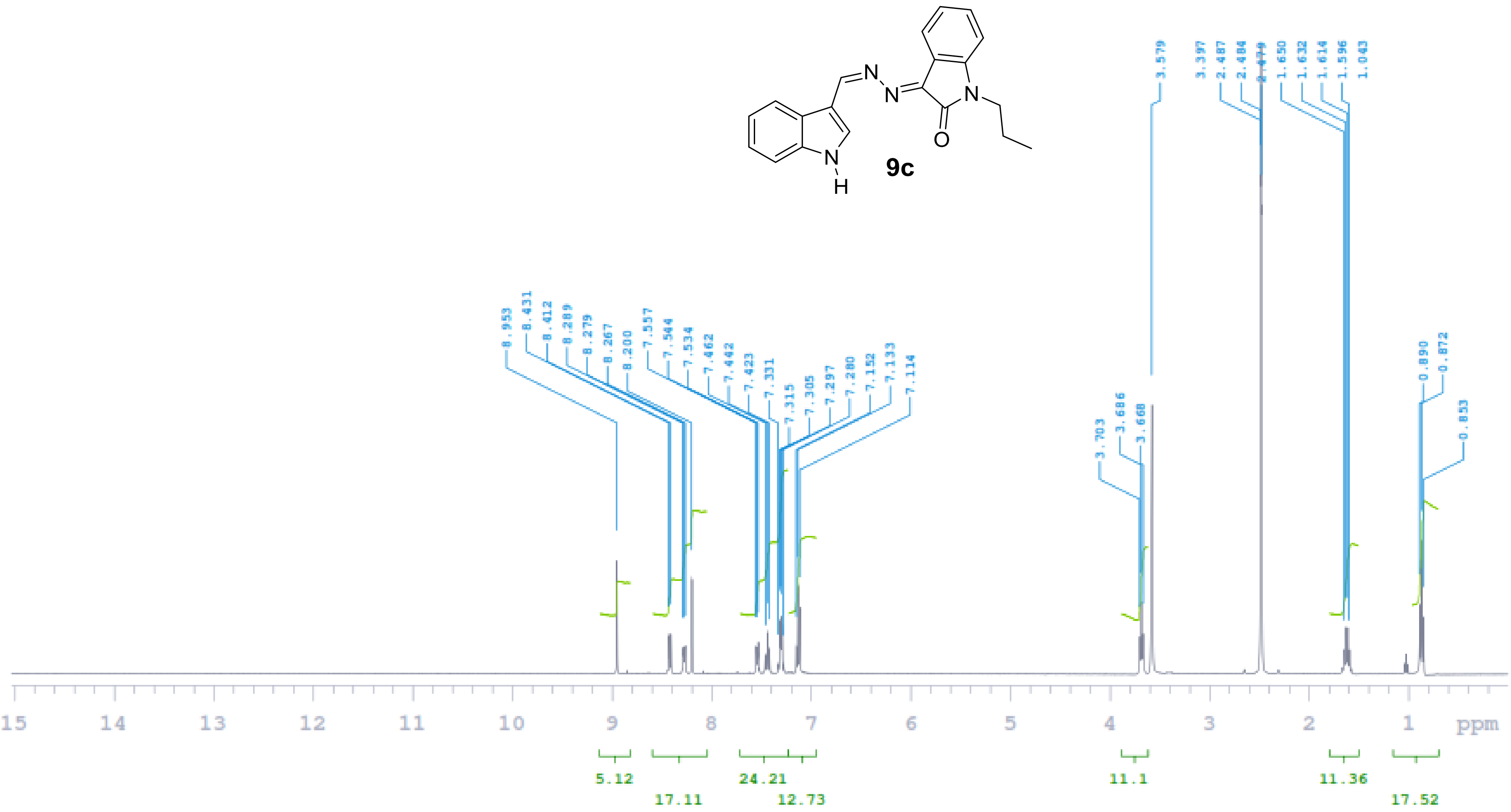

Dr_WagdyMohamed-Z9-C

Sample Name **Dr_WagdyMohamed-Z9-C** Pulse sequence **CARBON** Temperature **25** Study owner **vnmr1**
Date collected **2016-12-14** Solvent **dmso** Spectrometer **nmr400-mercury400** Operator **vnmr1**

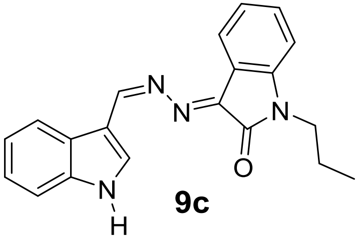

9c

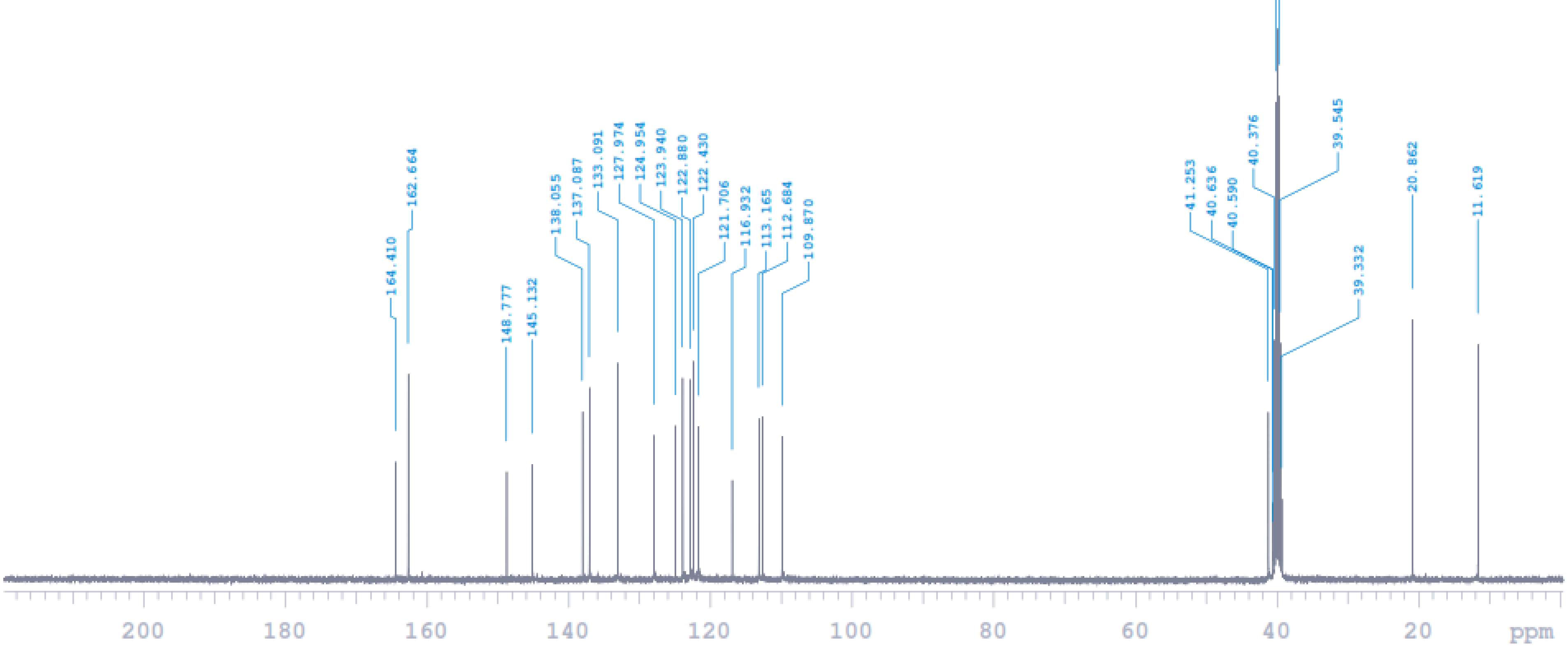

Dr_WagdyMohamed-Z9-D

Sample Name	Dr_WagdyMohamed-Z9-D	Pulse sequence	PROTON	Temperature	25	Study owner	vnmr1
Date collected	2016-08-06	Solvent	dmso	Spectrometer	nmr400-mercury400	Operator	vnmr1

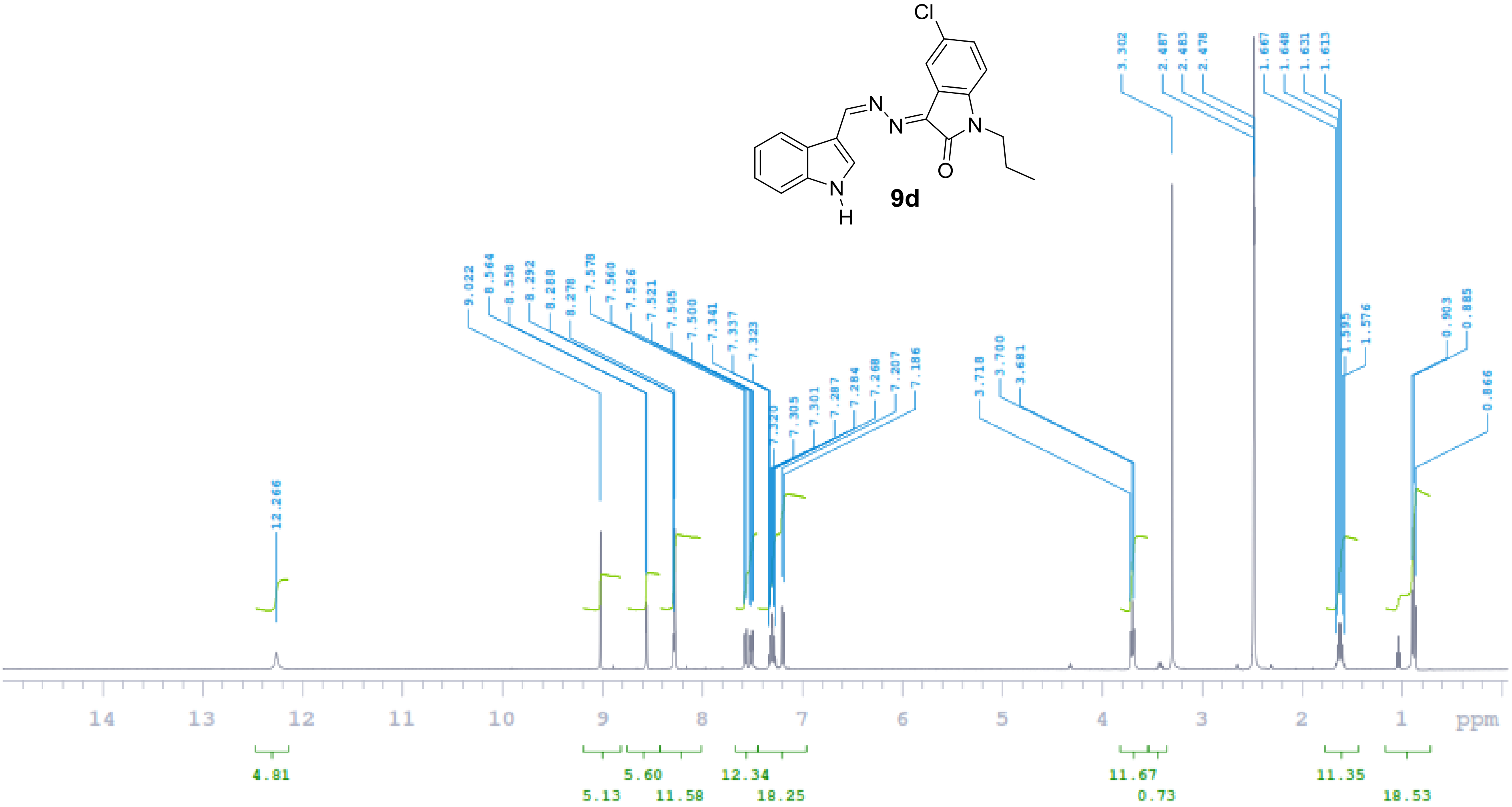

Dr_WagdyMohamed-Z9-E

Sample Name Dr_WagdyMohamed-Z9-E	Pulse sequence PROTON	Temperature 25	Study owner vnmr1
Date collected 2016-07-17	Solvent dmso	Spectrometer nmr400-mercury400	Operator vnmr1

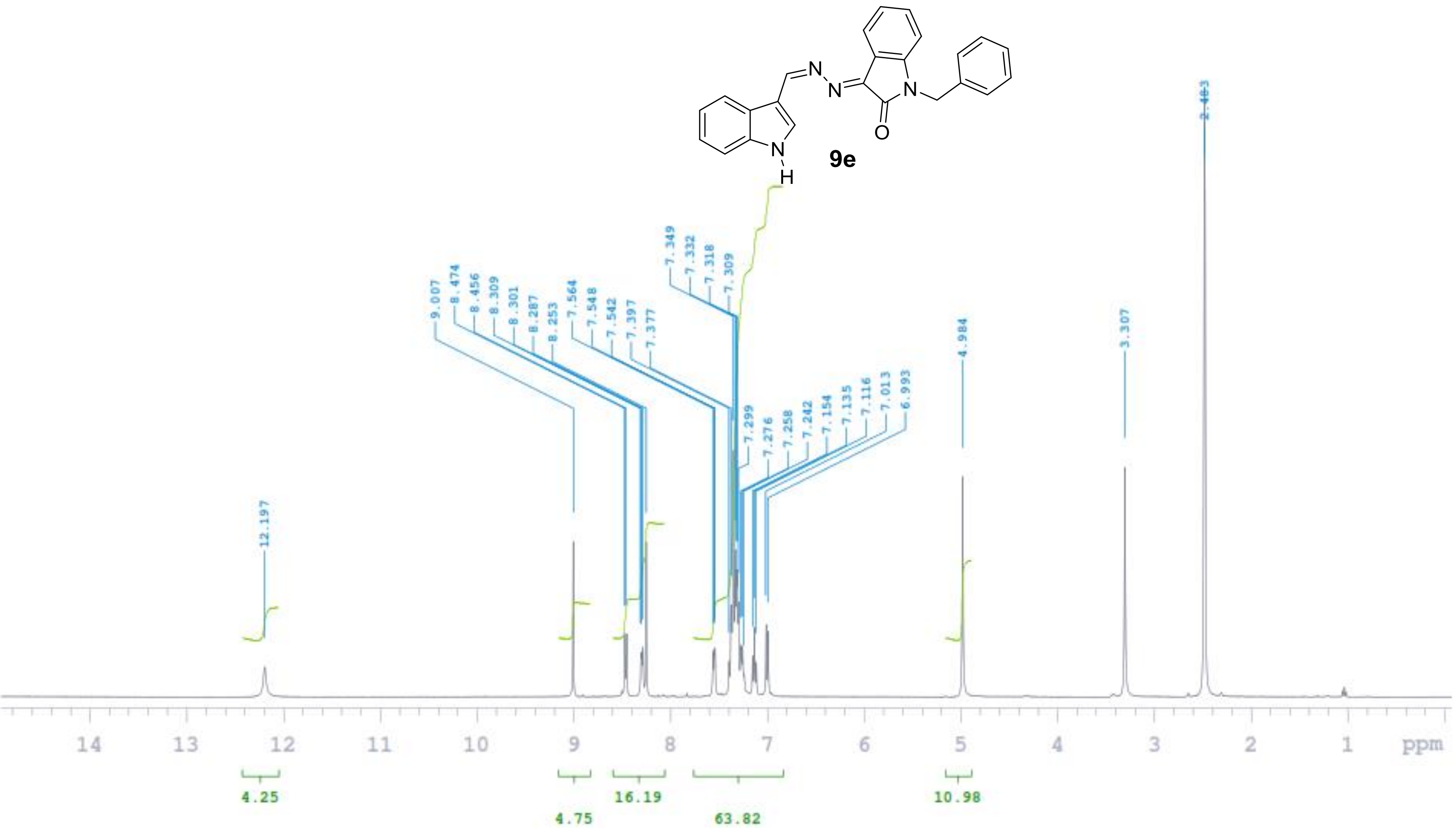

Dr_WagdyMohamed-Z9-E-D2O

Sample Name Dr_WagdyMohamed-Z9-E-D2O Pulse sequence PROTON Temperature 25 Study owner vnmr1
Date collected 2016-07-18 Solvent dmso Spectrometer nmr400-mercury400 Operator vnmr1

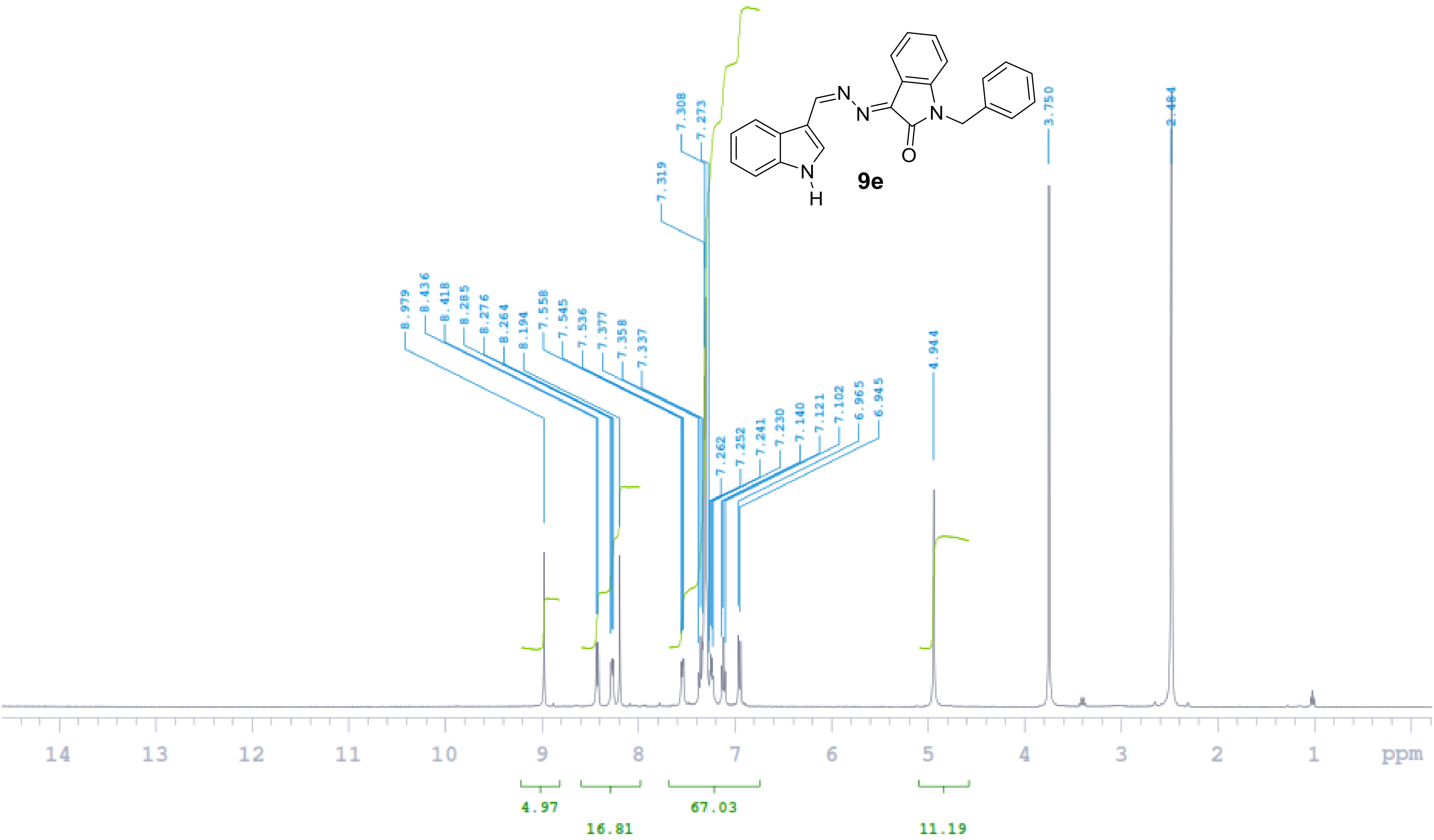

Agilent Technologies

Dr_WagdyMohamed-Z9-E

Sample Name	Dr_WagdyMohamed-Z9-E	Pulse sequence	CARBON	Temperature	25	Study owner	vnmr1
Date collected	2016-11-30	Solvent	dmso	Spectrometer	nmr400-mercury400	Operator	vnmr1

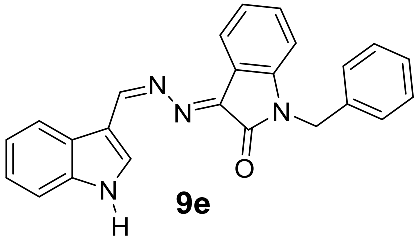

9e

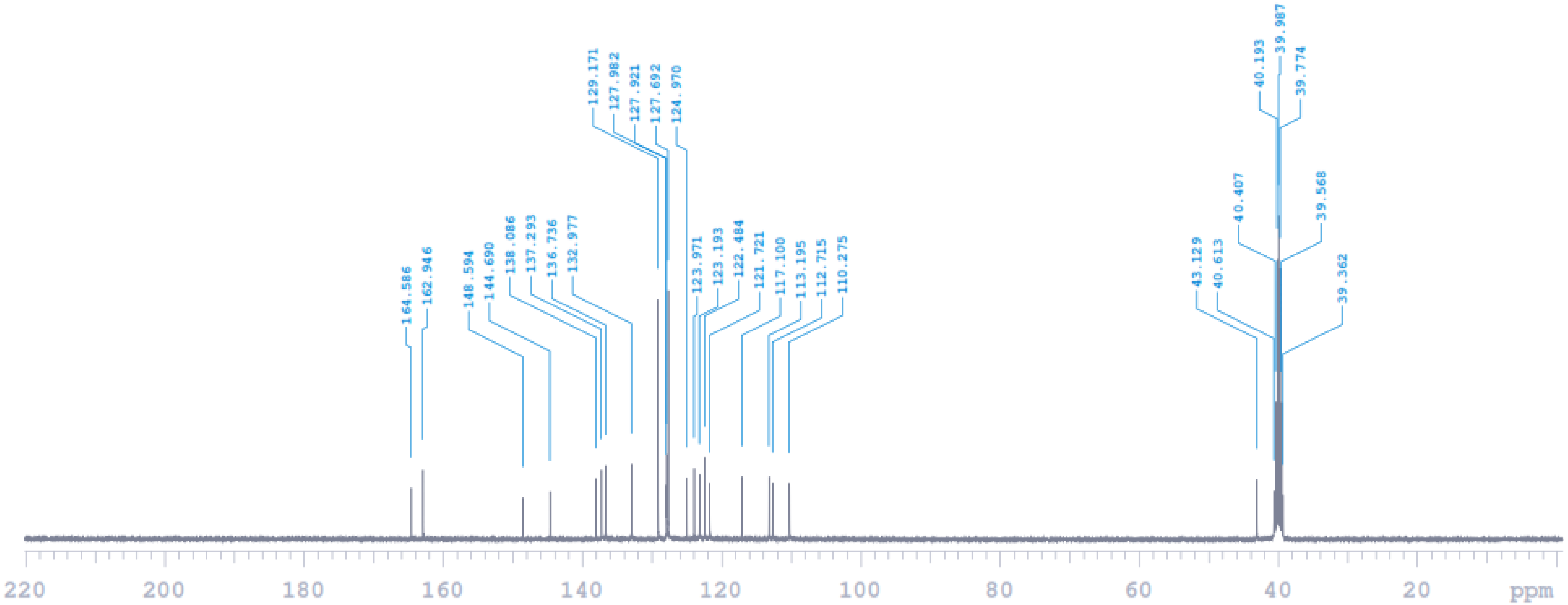

Dr_WagdyMohamed-Z9-F

Sample Name Dr_WagdyMohamed-Z9-F	Pulse sequence PROTON	Temperature 25	Study owner vnmr1
Date collected 2016-07-17	Solvent dmso	Spectrometer nmr400-mercury400	Operator vnmr1

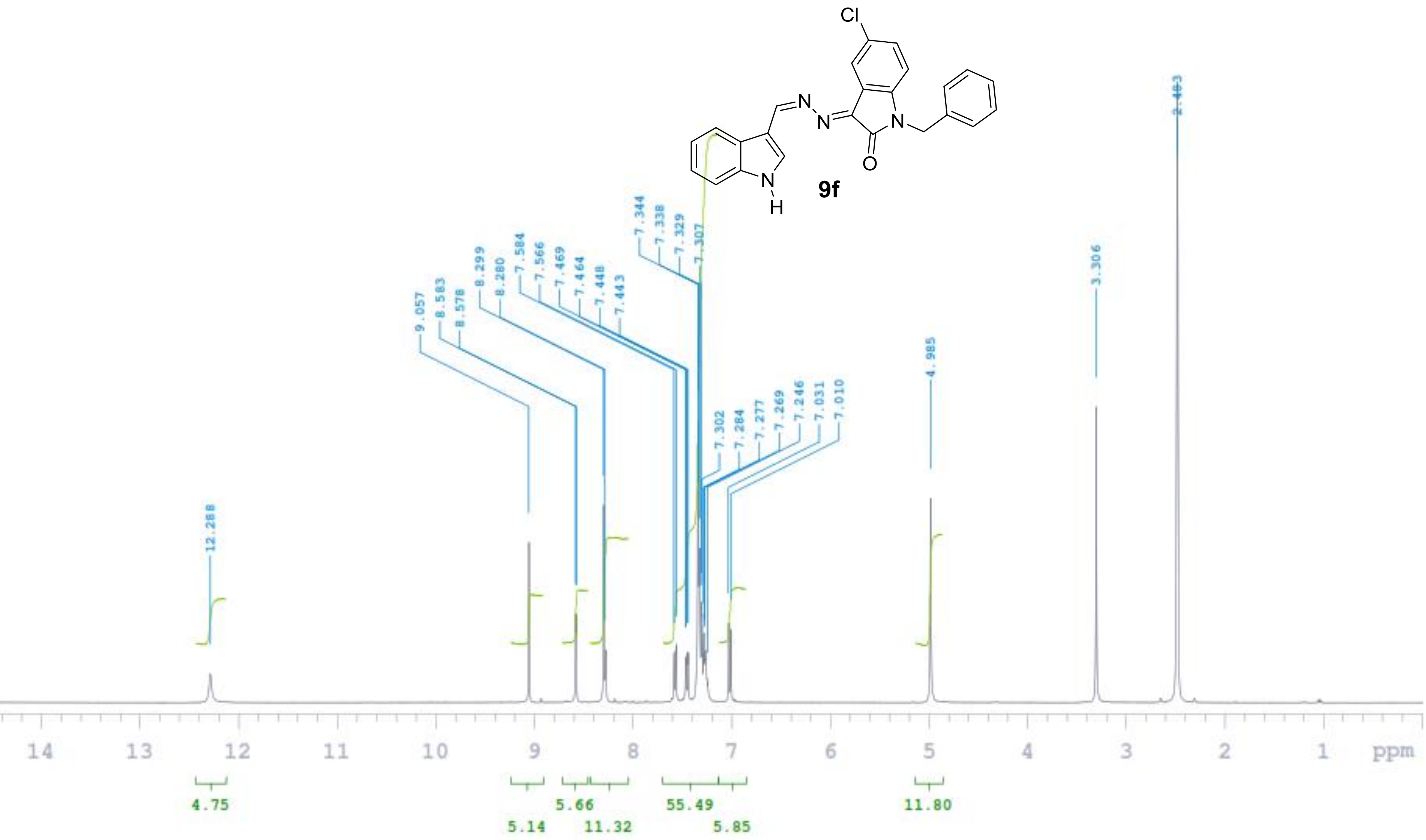

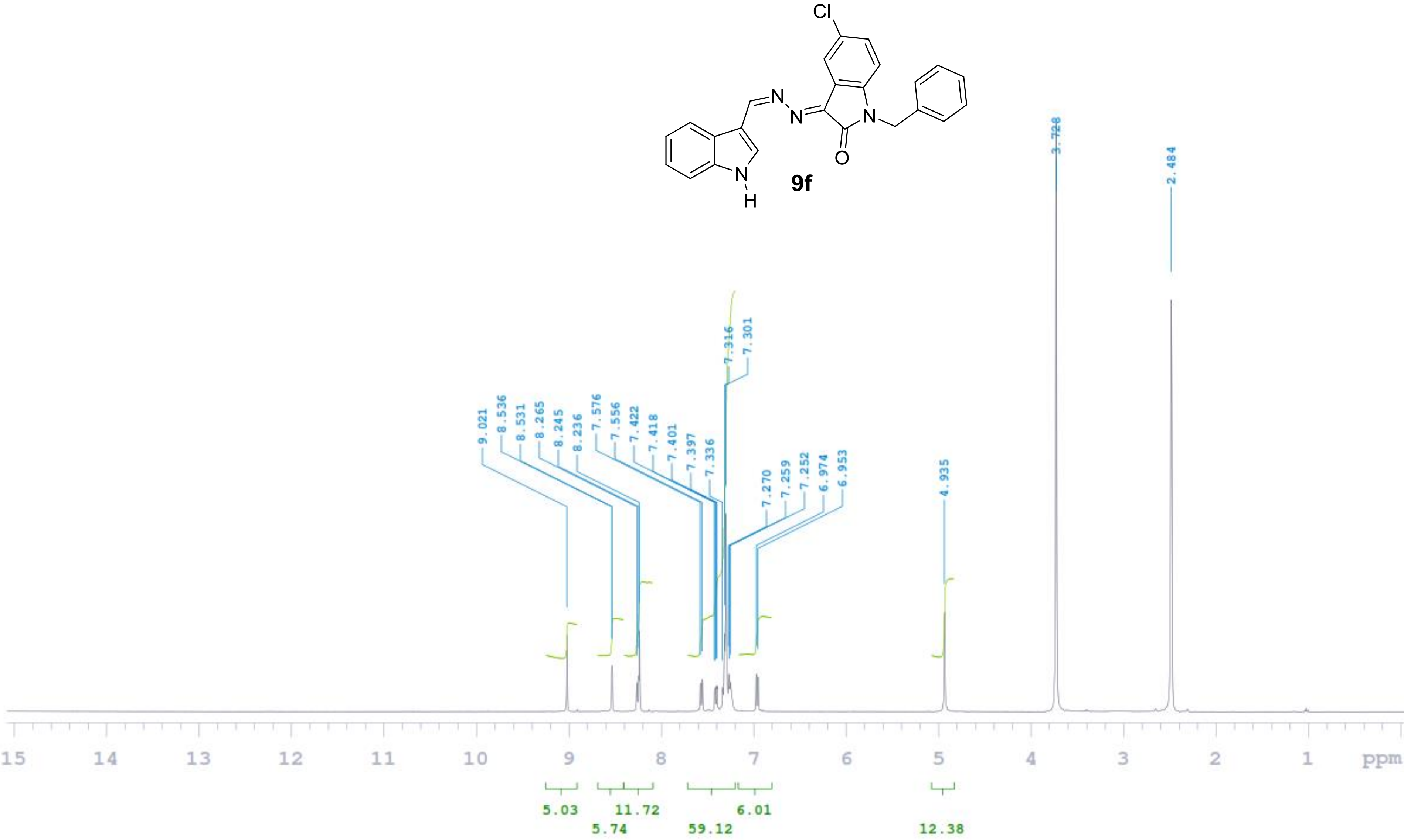

Cl
N
N
N
O
N
H
9f
9.021
8.536
8.531
8.265
8.245
8.236
7.576
7.556
7.422
7.418
7.401
7.397
7.336
7.316
7.301
7.270
7.259
7.252
6.974
6.953
4.935
3.728
2.484
15 14 13 12 11 10 9 8 7 6 5 4 3 2 1 ppm
5.03
5.74
11.72
59.12
6.01
12.38

Agilent Technologies

Sample Name	Dr_WagdyMohamed-Z9-F	Pulse sequence	CARBON	Temperature	25	Study owner	vnmr1
Date collected	2016-12-07	Solvent	dmso	Spectrometer	nmr400-mercury400	Operator	vnmr1

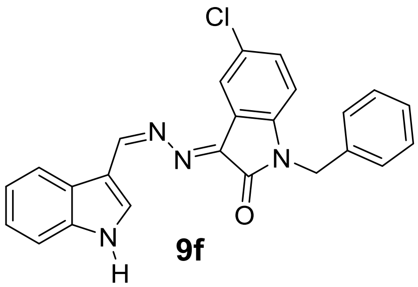

9f

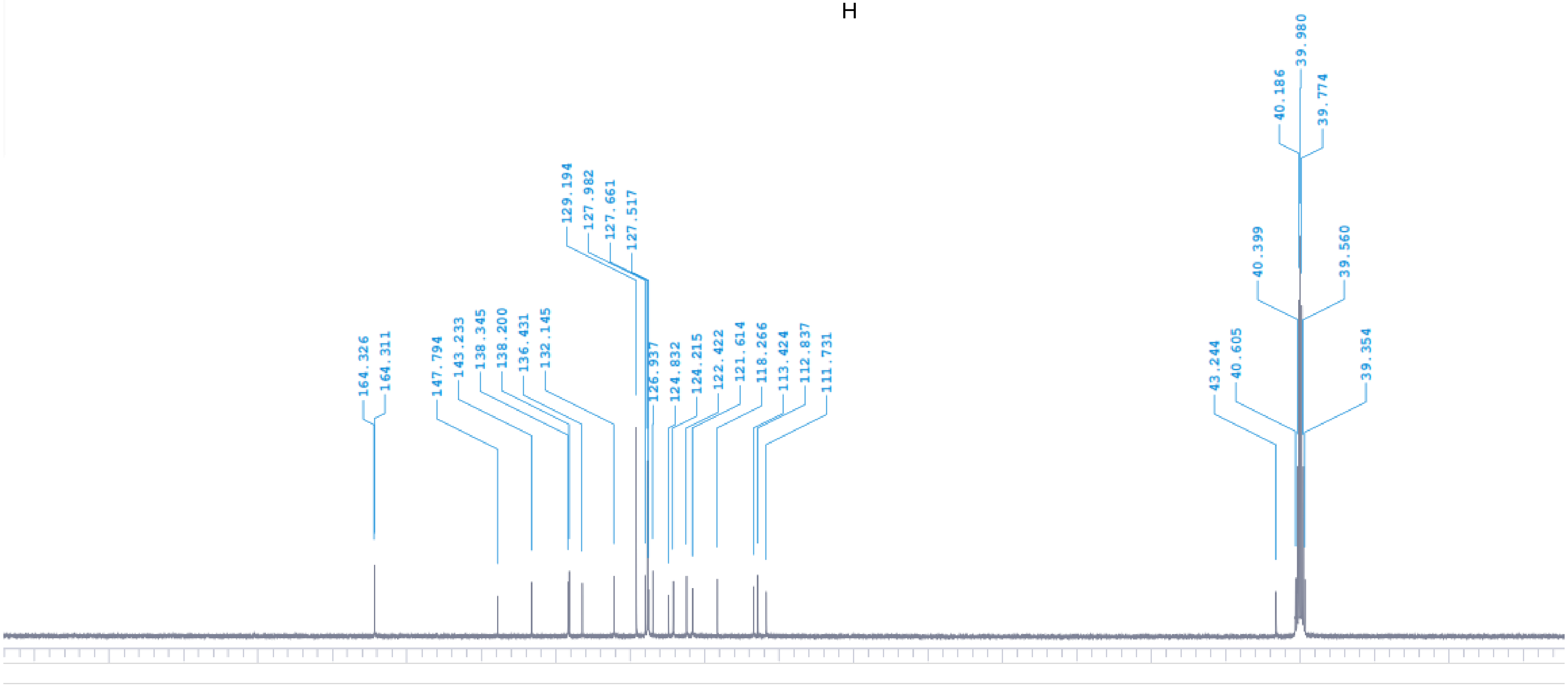

Supplement: IENZ_1421181_Supplementary_Material.pdf [file IENZ_A_1421181_SM4131.pdf]
